# Molecular Spiral Staircases: Stepwise Construction of Multilayer Phenylene Stacks

**DOI:** 10.1021/acs.orglett.6c02890

**Published:** 2026-08-25

**Authors:** Shuwei Ou, Jhih-Syong Jhang, Chih-hsiu Lin

**Affiliations:** † Department of Chemistry, 34911 National Central University, Taoyuan 320, Taiwan, Republic of China; ‡ Institute of Chemistry, 38017 Academia Sinica, Taipei 115, Taiwan, Republic of China

## Abstract

Stacked aromatic architectures represent important structural motifs in supramolecular chemistry, materials science, and molecular recognition. Herein, we report the modular synthesis of a series of covalently linked phenylene stacks containing up to six stacked phenylene units through consecutive Friedländer annulation reactions. The key structural motif is a bay-substituted 6,7-dihydrodibenzo­[*b*,*j*]­[4,7]­phenanthroline (DHDBPT) scaffold, which enforces close face-to-face arrangements between phenylene units. The resulting staircase-like architectures were characterized by spectroscopic techniques and single-crystal X-ray diffraction. Structural investigations further demonstrated distinct stereochemical behavior across the oligomer series. Odd-layer systems adopt *meso* configurations, whereas even-layer oligomers exhibit C2 chirality and can be resolved. The rotational barriers of terminal phenyl groups were shown to increase with stack thickness, suggesting increasing cooperative rigidity within the densely packed aromatic framework.

## Graphical abstract

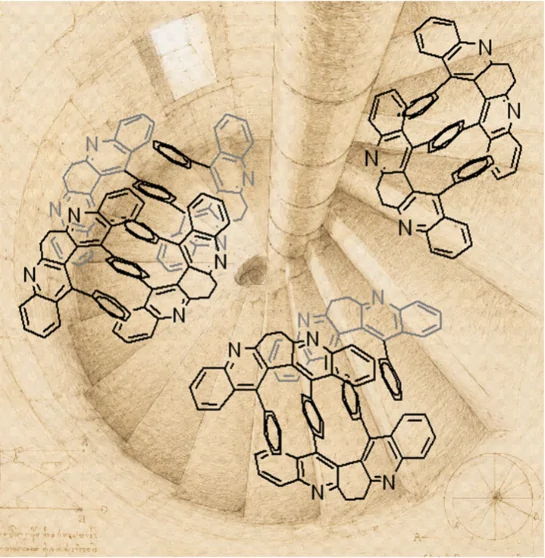

Stacked aromatic architectures are pivotal structural motifs at the cutting edges of contemporary chemistry.^1^ The columnar architectures of stacked aromatic systems provide unique opportunities to investigate interlayer charge transport, supramolecular organization, and collective conformational behavior, motivating extensive synthetic explorations.^2^ The face-to-face stacking arrangement serves as a versatile structural module that guides solid-state packing and supramolecular assembly, often giving rise to intriguing optoelectronic properties or coordinated motions relevant to molecular machines.^3^ Furthermore, π-stacking interactions are widely recognized as a fundamental driving force in biochemical processes, including protein folding, protein–protein complexation, and protein–substrate interactions.^4^ Lastly, the nature and structural dependence of π-stacking interactions continue to invite scientific inquiry.^5^ Notably, the term “π-stacking” encompasses a complex interplay of London dispersion, electrostatic, and solvophobic effects.^6^ To pursue a deeper understanding and broader applications of π-stacking interactions, considerable synthetic efforts have been devoted to constructing stacking architectures of aromatic units linked through rigid covalent scaffolds (Figure 1).

**Figure 1:**
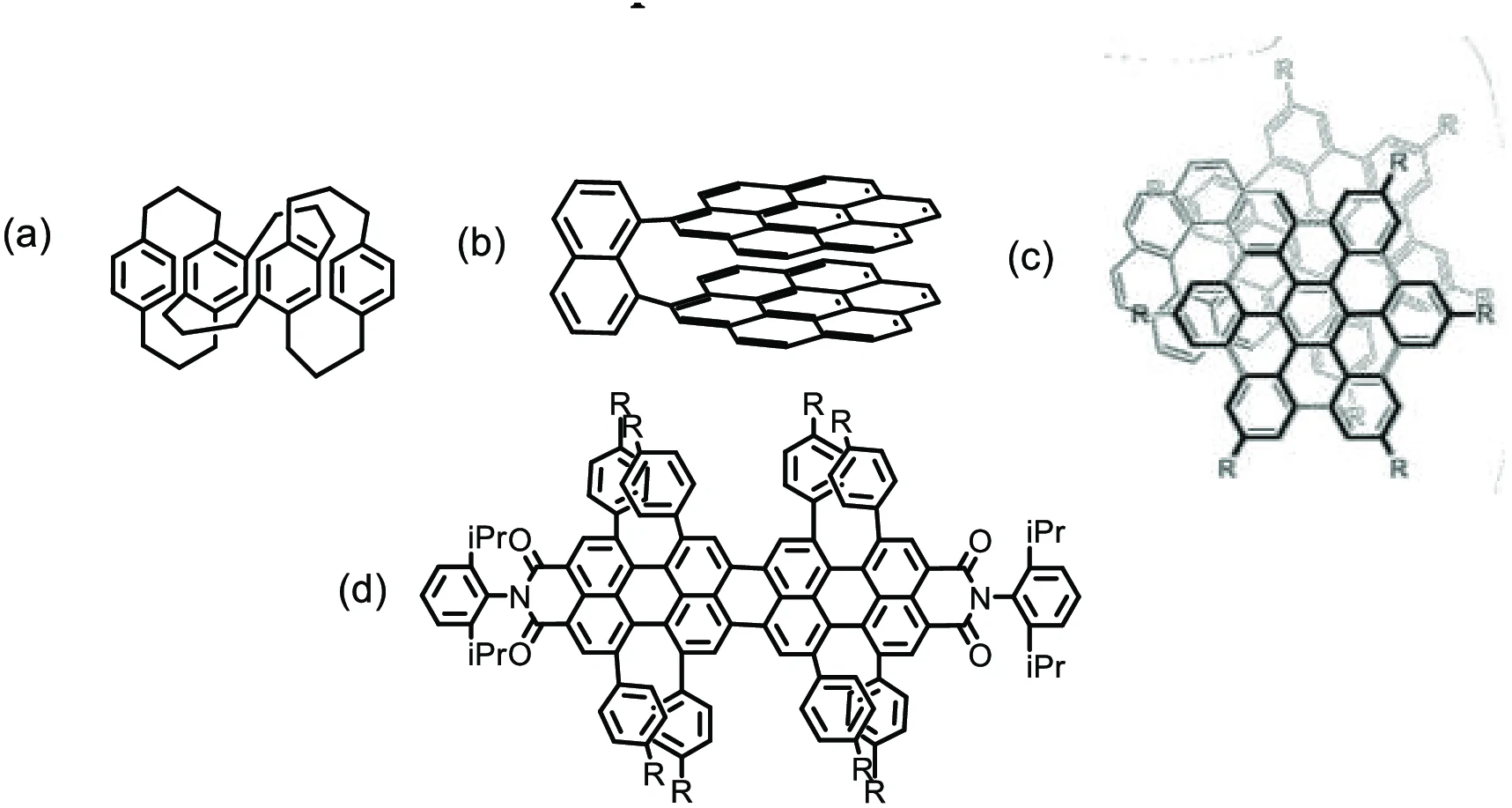
Examples of covalently linked π–π stack systems.

In the early 1970s, chemists built multilayer benzene stacks using [2,2]­cyclophane as building blocks.^7^ Later, a similar class of compounds featuring a columnar stacking architecture was synthesized by Shinmyozu’s group with [3.3]­cyclophane as the propagating units (Figure 1a).^8^ The zigzag edges of naphthalene and higher acenes represent another rigid scaffold often employed to construct face-to-face stacking dimers of polycyclic aromatic hydrocarbons (PAHs) (Figure 1b).^9^ Martin, Tan, and Feng independently achieved the synthesis of stacking dimers and trimers of hexabenzocoronene (HBC) using helicene scaffolds to direct the alignment of HBC moieties (Figure 1c).^10^ An underexplored scaffold for directing aromatic stacking is the armchair edge of PAHs, commonly denoted as the bay region. Parent compound 4,5-diphenylphenanthrene was synthesized more than five decades ago.^11^ Incorporating this stacking motif into the bay region of rylene systems to induce chirality was accomplished only recently via Suzuki cross-coupling reactions (Figure 1d).^12^ An aldehyde–alkyne annulation reaction that selectively produces bay-substituted phenanthrene derivatives was recently described by Itami.^13^ Nonetheless, the scope of reactions capable of constructing bay-substituted phenanthrene derivatives remains limited. As a result, no multilayer systems based on this scaffold have been reported.

Recently, our group developed a synthetic approach to a two-layer phenyl stack through a 2-fold Friedländer annulation reaction (Scheme 1a).^14^ Under acid-catalyzed conditions, the condensation of 2-aminobenzophenone with 1,4-cyclohexadione unexpectedly yielded the more sterically hindered product, bay-substituted 6,7-dihydrodibenzo­[*b*,*j*]­[4,7]­phenanthroline (DHDBPT). In the previous study, the π-stacking interactions between the bay region phenyl substituents were examined by single-crystal X-ray diffraction and variable-temperature NMR spectroscopy.^15^ Given the broad substrate scope and operational simplicity of this annulation, this methodology provides a modular route for constructing multilayer aromatic stack systems. The current synthetic targets are a series of oligomeric DHDBPT derivatives containing shared bay-substituted phenylene units. Retrosynthetic analysis identifies **A** (1,4-phenylenebis­((2-aminophenyl)­methanone)), which contains two 2-aminophenone moieties, as a pivotal bifunctional precursor for all subsequent synthetic elaborations (Scheme 1b).

**Scheme 1:**
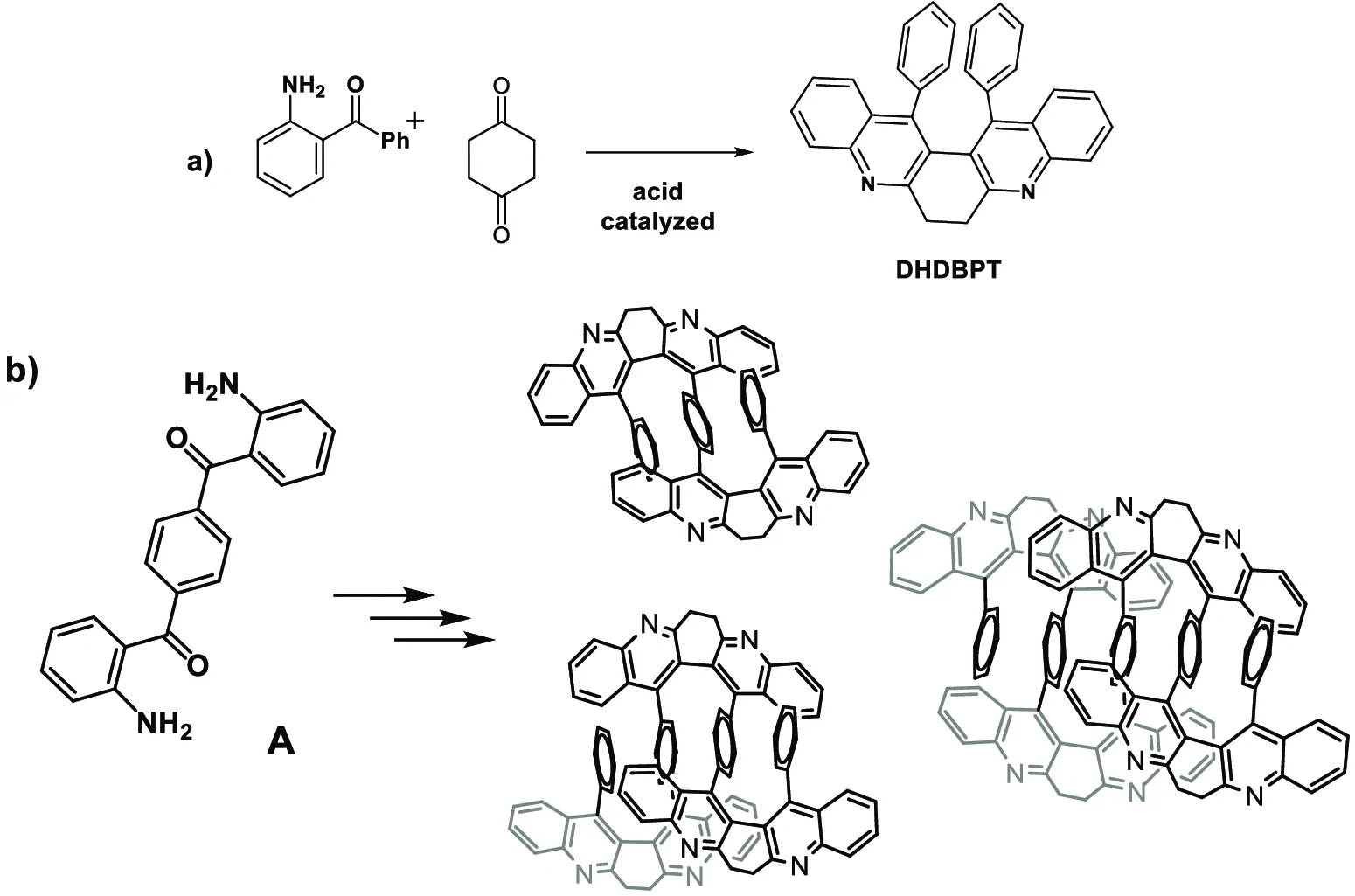
(a) Friedländer Condensation Route toward Bay-Substituted DHDBPT and (b) Retrosynthetic Analysis of Oligomeric DHDBPT Derivatives Containing Multilayer Phenylene Stacking Motifs

The synthesis of key building block **1** (Boc-protected **A**) is depicted in Scheme 2. Boc-protected aniline was first deprotonated using excess *t*-BuLi to furnish the ortholithiated intermediate.^16^ After the intermediate underwent double addition to terephthalaldehyde, a diol product was obtained. Without purification, the crude diol was oxidized by using Dess-Martin periodinane to furnish diketone **1**.

**Scheme 2:**
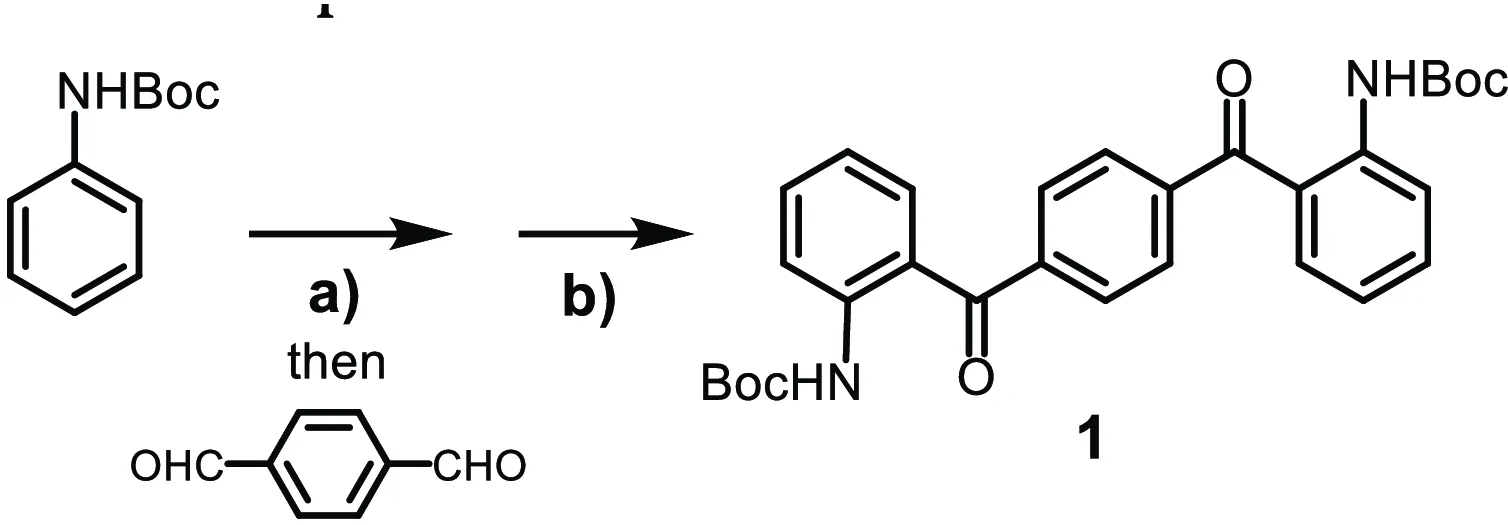
Synthesis of Building Block **1** for the Synthesis of Multilayer Phenylene Oligomers^a^

a Conditions: (a) 3 equiv of *t*-BuLi in ether; (b) Dess-Martin periodinane, CH_2_Cl_2_, 40% over two steps.

The synthesis of three-layer compound **3** is shown in Scheme 3. The 2-fold Friedländer condensation between building block **1** and terminal unit **2** was conducted in a 1:2.5 molar ratio (Scheme 3). (**2** was synthesized by the condensation of 2-aminobenzophenone and excess 1,4-hexadione as previously reported.^15^) Under the optimized condition (8% trifluoroacetic acid in refluxing dichloroethane), the desired product was obtained in 30% yield after purification by centrifugation. According to the molecular model of **3**, the middle phenylene moiety in the stack cannot freely rotate because its rotation is restricted by the two outer phenyl groups. The ^1^H NMR spectrum of **3** is consistent with this stacking arrangement. The two upshifted aromatic signals (Table S1) represent the static middle phenylene group in a rigid 2-fold symmetric environment.

**Scheme 3:**
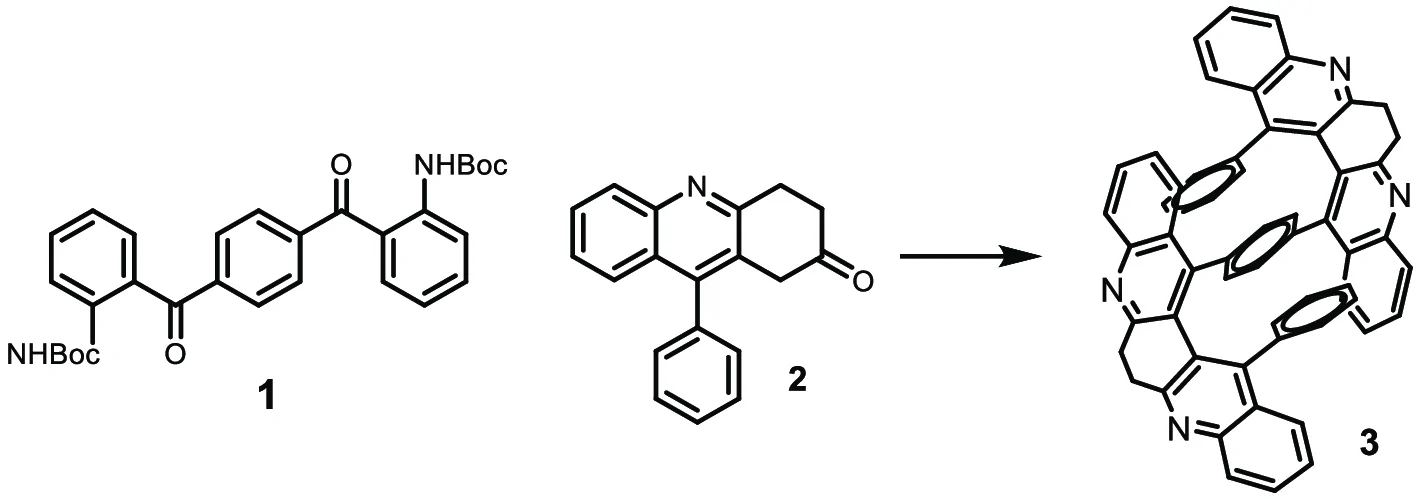
Synthesis of **3** Featuring a Three-Layer Phenylene Stack^a^

a Conditions: 1:2.5 **1**:**2**, 8% trifluoroacetic acid in refluxing dichloroethane, 4 h.

After successfully building the three-layer stacking structure in **3**, we proceeded to construct **5**, a four-layer stacking architecture. Due to the symmetry of the target structure, a [2+2] retrosynthetic approach was adopted. As shown in Scheme 4, building block **4** containing two stacked phenyl moieties was synthesized from **1** and **2** (1.3:1 molar ratio) under the standard Friedländer condensation condition. (In addition to the condensation reaction, the Boc group was also removed under the acidic condition.) The use of excess **1** ensures that desired product **4** retains a 2-aminophenyl ketone moiety as the module for further elongation. To construct the central DHDBPT unit in **5**, compound **4** then undergoes Friedländer condensation with 1,4-cyclohexadione in a 2.5:1 ratio (Scheme 4). The reaction delivers **5** in a moderate yield (25%).

**Scheme 4:**
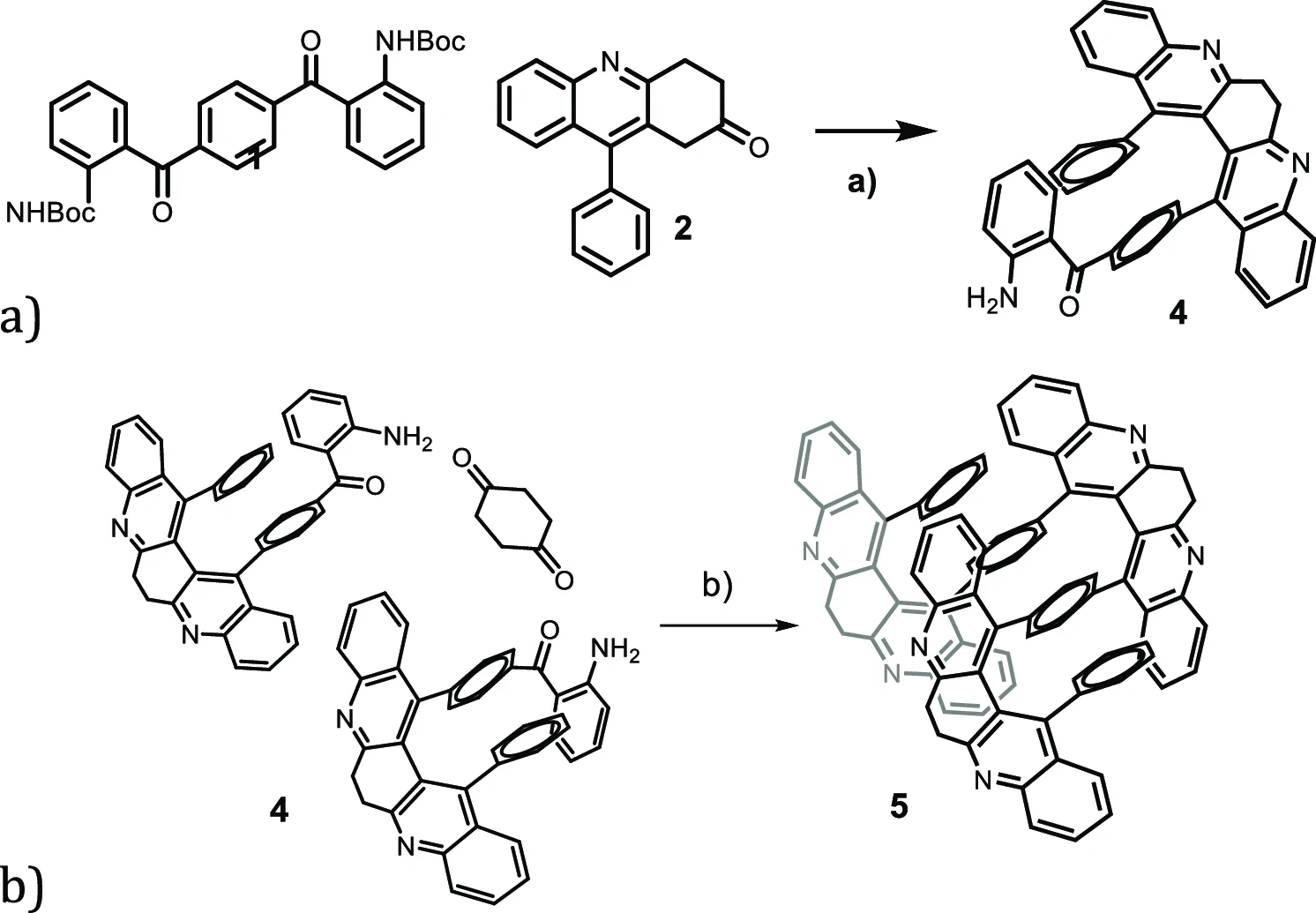
Synthesis of **5** Featuring a Four-Layer Phenylene Stack^a^

a Conditions: (a) 1.3:1 **1**:**2**, 8% trifluoroacetic acid in refluxing dichloroethane, 2.5 h, 35%; (b) 2.5:1 **4**:1,4-cyclohexadione, 8% trifluoroacetic acid in refluxing dichloroethane, 6 h, 25%.

The four-layer stacking architecture of **5** was corroborated by its ^1^H NMR spectrum. Like the central phenylene unit in **3**, the two inner phenylene units in **5** cannot freely rotate. As a result, the four protons on these phenylene units are nonequivalent and give rise to four signals (Table S1) in the upfield region. According to molecular modeling, **5** belongs to the *C*2 point group, which predicts three types of nonequivalent quinoline and methylene units in **5**. This symmetry is verified by the ^13^C NMR spectrum as three distinct signals are observed in the characteristic quinoline region and sp^3^ region (Table S1), respectively.

Accordingly, five-layer phenylene stack compound **7** should comprise four DHDBPT linkers connecting five phenylene units. A reasonable retrosynthesis for this structure is a [2+1+2] tandem condensation between appropriate building blocks **4** and **6**. Central unit **6** was synthesized via Friedländer condensation of **1** with excess 1,4-cyclohexadione (Scheme S1). The subsequent 2-fold Friedländer condensation between compounds **4** and **6** (Scheme 5) was conducted under the standard conditions. The desired product, compound **7**, featuring a five-layer phenylene stack was successfully prepared. The proposed molecular formula of **7** (C_110_H_71_N_8_) was verified by high-resolution mass spectrometry ([M + H]^+^, 1503.5802; found, 1503.5810).

**Scheme 5:**
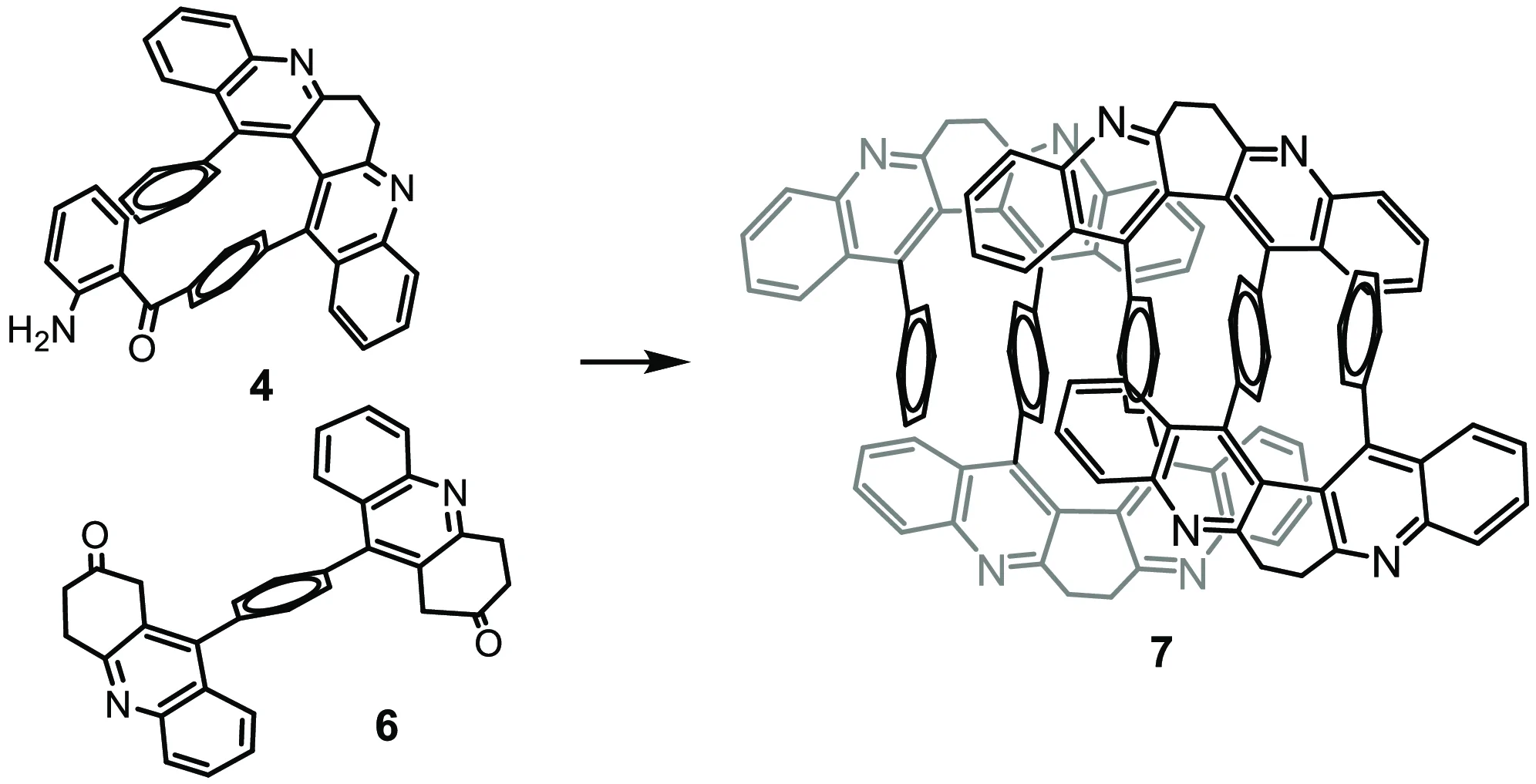
Synthesis of Oligomer **7** Featuring a Five-Layer Phenylene Stack^a^

a Conditions: 2.5:1 **4**:**6**, 8% trifluoroacetic acid in refluxing dichloroethane, 8 h, 18%.

The stacking architecture of compound **7** was supported by its ^1^H and ^13^C NMR spectra. Similar to compounds **3** and **5**, the three inner phenylene units in **7** are static on the NMR time scale. Since the center of inversion in this structure is located at the midpoint of the central phenylene unit, the four protons on this ring give rise to two distinct signals. The other two inner phenylene rings, while symmetry-related, reside in asymmetrical environments. Consequently, the eight protons on these phenylene units appear as four sets of signals. Altogether, the 5.5–6.5 ppm region of the ^1^H NMR spectrum of **7** is expected to contain six aromatic signals, which is consistent with the observed spectrum (Table S1). Furthermore, the eight quinoline and eight methylene units in compound **7** are likewise symmetry-related by its center of inversion. Accordingly, four sets of signals are anticipated for both types of groups, which is also corroborated by the experimental ^13^C spectrum (Table S1).

Finally, the six-layer phenylene stack in compound **10** was constructed by employing a [2+2+2] consecutive Friedländer condensation. Required building block ketone **8** was synthesized by Friedländer condensation of **3** with excess 1,4-cyclohexadione. Diamino-diketone central unit **9** was assembled by condensing excess **1** with 1,4-cyclohexadione (Scheme S2).

The 2-fold condensation between **8** and **9** (2.6:1 (Scheme 6)) successfully furnished target six-layer phenylene stack **10**. High-resolution mass spectrometry confirms the expected molecular formula of **10** (C_136_H_87_N_10_; calcd for [M + H]^+^, 1859.7115; found, 1859.7120). As previously described for **3**, **5**, and **7**, the six-layer stacking motif was established through the ^1^H and ^13^C NMR spectroscopic patterns. The 16 protons on the four static inner phenylene moieties are symmetrically related by a *C*2 axis and therefore give rise to eight signals in the ^1^H NMR upfield region. This is indeed substantiated experimentally (Table S1). Owing to the *C*2 symmetry element, the 10 quinoline and 10 methylene units in **10** likewise result in five corresponding signals in the expected regions of the ^13^C spectrum (Table S1, two coinciding at 34.39 ppm).

**Scheme 6:**
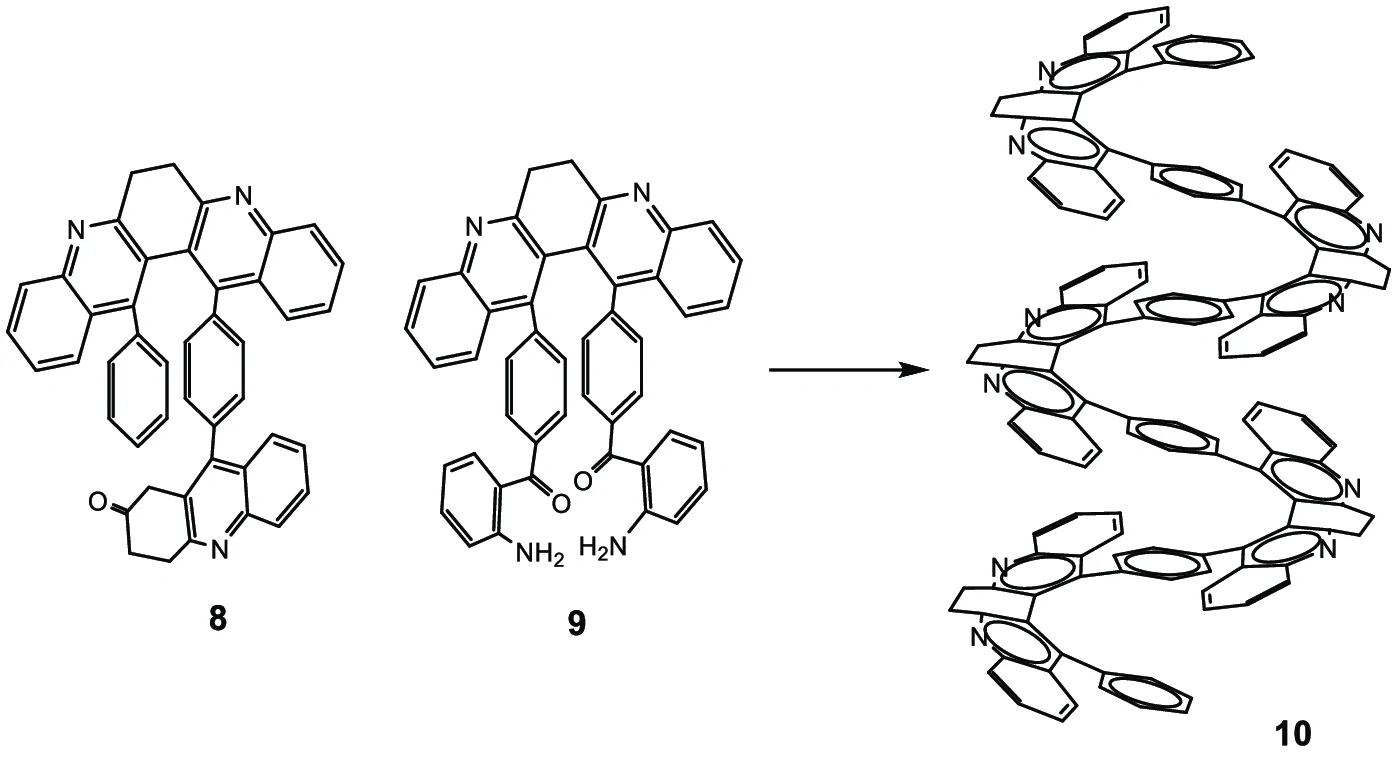
Synthesis of **10** Featuring a Six-Layer Phenylene Stack^a^

a Conditions: 2.6:1 **8**:**9**, 8% trifluoroacetic acid in refluxing dichloroethane, 10 h, 5%.

Single crystals of these staircase-like molecules cannot be obtained because these compounds form aggregates upon prolonged standing. This problem was addressed by preparing *tert*-butyl-substituted three-layer compound **11** and four-layer compound **12** from 4-*tert*-butylaniline using procedures analogous to those shown in Schemes 2–4 (Schemes S3 and S4). With the improved solubility, single crystals suitable for X-ray crystallography were obtained. The stacking motifs were unambiguously established for both compounds (Figures S5 and S6), providing strong support for the stacking arrangements assigned to compounds **3**, **5**, **7**, and **10**. The upfield chemical shifts of the protons on the inner phenylene units can be attributed to their close contact (3.02–3.04 Å) with neighboring stacking phenyl groups.

Three-layer stacking compound **13** with a shared *m*-phenylene unit was also prepared. The synthesis of this compound follows a strategy identical to that employed for **3** except for the modification that isophthalaldehyde was used in place of terephthalaldehyde (Scheme S5). Notably, unlike most *meta*-substituted benzene derivatives bearing two identical substituents, compound **13** does not possess a *C*2 axis or a reflection plane. This low molecular symmetry arises from its three-layer stacking architecture. First, the stacking arrangement restricts the rotation of the DHDBPT groups. Additionally, the two outer phenyl groups occupy distinct spatial environments relative to the central phenylene unit. Consequently, the two DHDBPT substituents, though topologically identical, are conformationally distinct. This unique situation results in the asymmetric structure of **13**, which is evident from its ^1^H and ^13^C NMR spectra. The upfield region of the ^1^H NMR spectrum (5.2–7.1 ppm) contains 14 signals, confirming the absence of symmetry elements. Similarly, four signals corresponding to four distinct quinoline units are observed in the ^13^C NMR spectrum (162.74, 162.66, 162.30, and 161.92 ppm), in agreement with the structure of *C*1 symmetry. The crystal structure of **13** (Figure S7) also confirms the unusual architecture; the two enantiomers of **13** crystallize in the centrosymmetric *P*21/*n* space group. The ^1^H NMR spectrum of **13** exhibits signals further upfield (up to 5.1 ppm) than those of other compounds discussed previously. This can likely be attributed to the closer H-π-surface contacts in **13** (2.68 Å).

Beyond demonstrating synthetic accessibility, these oligomers provide an opportunity to investigate how increasing the stack thickness influences molecular symmetry, chirality, and conformational dynamics. At first glance, because of the inherently helical stacking arrangement in the bay region of DHDBPT, all compounds appear to be optically active. However, a closer examination reveals that three-layer oligomer **3** (*M,P*) and five-layer oligomer **7** (*M,P,M,P*) both possess centers of inversion, which classifies them within the *Ci* point group. The *meso* configuration of the odd-layer oligomers is exemplified in the crystal structure of **11**, which adopts centrosymmetric space group *P*1̅. In contrast, the even-layer oligomers, namely, **5** (*M,P,M* and *P,M,P*) and **10** (*M,P,M,P,M* and *P,M,P,M,P*), belong to the *C*2 point group and are therefore chiral. This is demonstrated in the crystal structure of **12**, in which the two enantiomers pack in a centrosymmetric *P*21/*n* unit cell. These stereochemical assignments were further corroborated by chiral high-performance liquid chromatography (HPLC). As expected, no separation was achieved for compound **3** or **7** (Figures S1 and S2). Meanwhile, four-layer oligomer **5** displayed two well-resolved peaks (Figure S3). The circular dichroism spectra of the two components isolated from racemic **5** are mirror images of each other (Figure S4), confirming their enantiomeric nature. The striking feature of the DHDBPT architecture revealed by the present study is that the odd-layer oligomers possess inversion symmetry whereas the even-layer oligomers are inherently chiral.

In reported stacked aromatic architectures, such systems often serve as models to understand interchromophore interactions.^2c,17^ In the present study, we examined how the rotational dynamics of the terminal phenyl groups are geared by stacks of phenylene units with increasing thickness. When phenyl rotation is slow on the NMR time scale, the inequivalent *ortho* protons give rise to distinct signals. As rotation accelerates, the two *ortho* protons become equivalent, leading to signal coalescence. By measuring coalescence temperatures for each compound using variable-temperature NMR experiments, we estimated the corresponding rotational barriers using the Eyring equation.

For the two-layer compound, the rotational barrier was previously determined to be 13.4 kcal/mol. Compounds **3** and **5**, which contain three- and four-layer stacks, exhibit slightly higher barriers at 13.6 and 13.7 kcal/mol, respectively. The rotational barrier further increases in five-layer compound **7** to 14.1 kcal/mol (the rotational barrier for oligomer **10** cannot be reliably measured due to low solubility). Given the similar steric environments surrounding the terminal phenyl groups across the series, the increasing trend is best attributed to the growing rigidity imposed by thicker phenylene stacks. For phenyl rotations to occur, adjacent DHDBPT scaffolds must undergo distortion. In higher oligomers, such a rotation necessitates collective deformation of multiple DHDBPT units, thereby increasing the associated energetic cost. Although the increase in energy barriers is modest, the monotonic trend observed across the series suggests a genuine increase in cooperative rigidity with stack thickness.

In conclusion, multilayer phenylene stacks resembling spiral staircases were constructed through consecutive Friedländer annulation reactions. These staircase-like architectures consist of stacked phenylene units using bay-substituted DHDBPT linkers as lateral rails. The aesthetically intriguing structures were confirmed by NMR spectroscopy and X-ray crystallography. Despite the helical conformation of the DHDBPT scaffold, odd-numbered oligomers **3** and **7** adopt *meso* configurations, verified through chiral HPLC. In contrast, the even-numbered oligomers exhibit *C*2 chirality, manifested through chiral HPLC and the circular dichroism spectrum of compound **5**. The rotational barriers of the terminal phenyl groups in these stacks were investigated using variable-temperature NMR. The increasing rotational barriers suggest that the densely packed phenylene cores undergo increasingly cooperative structural deformation to accommodate phenyl rotation. This work establishes DHDBPT as a robust and synthetically accessible scaffold for positioning functional groups in well-defined spatial proximity. As such, this structural motif serves as a versatile module for investigating and engineering noncovalent interactions. The iterative nature of the synthetic strategy should enable the preparation of aromatic heterostacks as platforms for studying charge separation, exciton migration, and mechanically coupled molecular systems. Overall, the ability to construct multilayer aromatic architectures with predictable stereochemical and dynamic behavior establishes DHDBPT as a versatile platform for the design of functional π-stacked molecular systems.

## Supplementary Material



## Data Availability

The data underlying this study are available in the published article and its Supporting Information.

## Notes

Synthetic procedures, spectroscopic characterization (^1^H NMR, ^13^C NMR, and HRMS data), crystal structures of **11**–**13**, chiral HPLC chromatograms of **3**, **5**, and **7**, and circular dichroism spectra of resolved **5** (PDF)
