# Supplementary material for: Molecular Spiral Staircases: Stepwise Construction of Multilayer Phenylene Stacks
Source: Org Lett. 2026 Aug 25;28(41):12861–6. doi: 10.1021/acs.orglett.6c02890 (PMC13682200; doi:10.1021/acs.orglett.6c02890)
Supplement: Supplementary file 1 [file ol6c02890_si_001.pdf]

Title: Molecular Spiral Staircases: Stepwise Construction of Multilayer Phenylene Stacks

Authors: Shuwei Ou[a], Jhih-Syong Jhang[b], Chih-hsiu Lin[b]*

a: Department of Chemistry, National Central University

b: Institute of Chemistry, Academia Sinica, Taiwan, Taipei, Republic of China

Email: chemopera@gate.sinica.edu.tw

Materials and general synthetic procedures……………………………………………………………..p2

Synthesis procedures for compounds **1-3**………………………………………………………………p3-6

Synthesis procedures for compounds **4-5** ……………………………………………………………p7-9

Synthesis procedures for compounds **6-7** (Scheme S1)…………………………………p10-13

Synthesis procedures for compounds **8-10** (Scheme S2)………………………………p14-18

Synthesis procedures for compounds **11-12** (Schemes S3 and S4)……………………p19-23

Synthesis procedure for compound **13** (Scheme S5)…………………………………………p24-27

^{1}H and ^{13}C for compounds **1**–**3** and HRMS spectrum for **3**………………………………p28-34

^{1}H and ^{13}C for compounds **4-5** and HRMS spectrum for **5**………………………………p35-40

^{1}H and ^{13}C for compounds **6-7** and HRMS spectrum for **7**………………………………p41-46

^{1}H and ^{13}C for compounds **8-10** and HRMS spectrum for **10** …………………………p47-55

Table S1: Fine spectral features of the ^{1}H and ^{13}C NMR spectra………………………………p56

^{1}H, ^{13}C, and HRMS spectra for compounds **11**–**12**……………………………………………p57-66

^{1}H and ^{13}C for compounds **C** and **13** and HRMS spectrum for **13**………………………p67-71

Variable-temperature experiments of compounds **3, 5,** and **7**……………………………p72-76

Chiral HPLC analyses of compounds **3**, **5**, and **7**………………………………………………p77

Circular dichroism spectra of enantiomers of **5….**………………………………………………p77

Crystallography data of compound **11**, **12**, and **13**…………………………………………p78-82

Materials, instrumentations, and general procedures: All starting compounds and reagents were purchased from commercial sources and used without further purification. All anhydrous solvents were purchased from commercial sources and used without further purification. All reactions were performed under one atmosphere of dry nitrogen or argon in oven-dried reaction vessels and stirred using magnetic stir bars. All heating was conducted using oil baths. All reactions were monitored by analytical thin-layer chromatography (TLC, 250 EM silica gel 60 F_{254} plates, visualized with a 254 nm UV lamp) or ^{1}H NMR. Flash column chromatography was performed with silica gel 60 (1.11567.9025, 0.040-0.063 mm) as the stationary phase. All eluent ratios are given by volume. ^{1}H and ^{13}C NMR spectra were recorded in $CDCl_3$ solutions. Inseparable grease impurities and residual CH_2Cl_2 were visible in some of the compounds. The high-resolution mass spectra under FAB ionization mode were obtained using a double-focusing magnetic-sector mass analyzer. The spectra from MALDI ionization mode were obtained using TOF analyzer. The single-crystal X-ray diffraction data were collected at 150 K on a Bruker D8 Venture diffractometer equipped with a Cu-target (Kα = 1.54184 Å) and Mo-target (Kα = 0.71073 Å) microfocus X-ray generators and a PHOTON-III CMOS detector. The chiral HPLC analysis was performed using a CHIRALPAK IE column (packing particle size: 5 μm, column size: 4.6 nm x 250 mm). The eluent was a mixture of tetrahydrofuran and ethyl acetate (1:1) with the flow rate fixed at 1.0 mL min^{-1}.

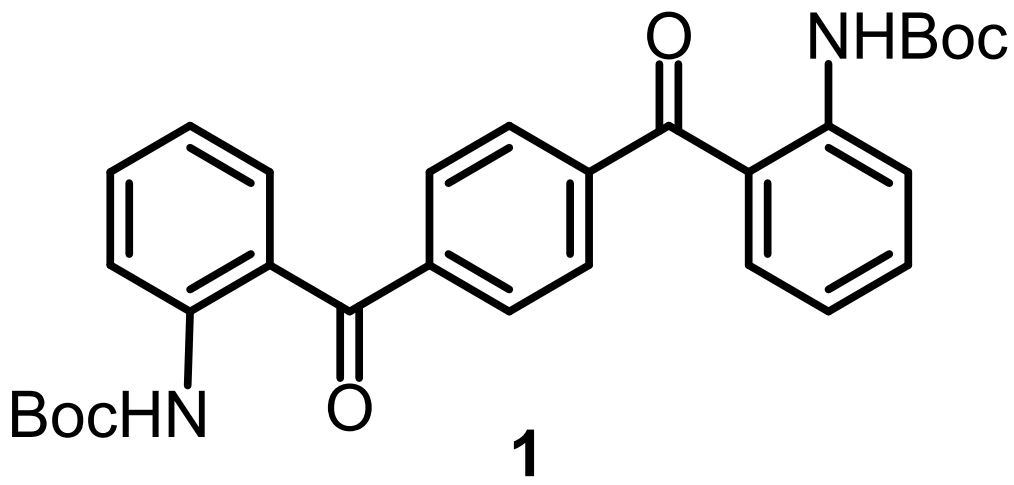

1

In a two-necked round-bottom flask, N-Boc aniline (5.6 g, 28.5 mmol, 3 equiv) was dissolved in dry diethyl ether (120 mL). The solution was cooled to -78 °C under nitrogen. t-BuLi (1.9 M in pentane, 40 mL, 76 mmol, 8 equiv) was slowly added to the solution. After the addition was completed, the reaction was kept at -78 °C for another 15 min before it was warmed to 0 °C. The deprotonation reaction was stirred at 0 °C for 90 min before a diethyl ether (140 mL) solution of terephthalaldehyde (1.27 g, 9.5 mmol, 1 equiv) was added. The reaction was stirred for another 90 min before it was quenched with saturated aqueous ammonium chloride. The solvent was removed under reduced pressure and the remaining crude diol was dissolved in anhydrous dichloromethane (5 mL/mmol). The solution was cooled to 0 °C before Dess-Martin periodinane (10.0 g, 2.5 equiv) was added. The oxidation was carried out at room temperature for 2.5 h before being quenched with saturated aqueous sodium bicarbonate. The organic layer was separated and dried over Na_2SO_4. After filtration, the solvent was removed under reduced pressure and the crude product and p-toluenesulfonyl hydrazide (880 mg, 0.5 equiv) were suspended

in ethanol (250 mL) and stirred for 1.5 h. The mixture was filtered and the soluble portion was concentrated under reduced pressure. The crude product was purified twice with flash chromatography (hexane: dichloromethane = 4:6) to give compound **1** as a white powder (1.96 g, 40%).

^{1}H NMR (500 MHz, $CDCl_3$): δ 10.17 (s, 2H), 8.48 (d, *J* = 7.0 Hz, 2H), 7.60 (t, *J* = 7.0 Hz, 2H), 7.54 (d, *J* = 7.0 Hz, 2H), 7.04 (t, *J* = 7.0 Hz, 2H), 1.56 (s, 18H); $^{13}C\{^1H\}$ NMR (125 MHz, $CDCl_3$): δ 198.6, 153.0, 141.85, 141.81, 134.8, 133.6, 129.5, 122.0, 120.9, 120.0, 80.8, 28.3. HRMS (MALDI) m/z: [M^+] calcd for $C_{30}H_{32}N_2O_6$: 516.2260; found 516.2257.

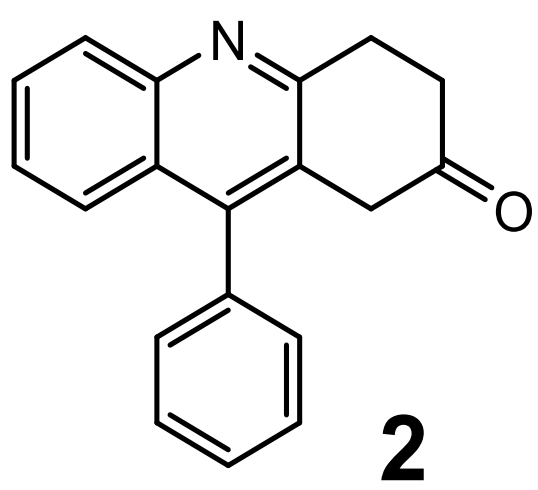

2

In a pressurized tube, 2-aminobenzophenone (500 mg, 2.54 mmol, 1 equiv) and 1,4-cyclohexadione (854 mg, 7.62 mmol, 3 equiv) were dissolved in anhydrous 1,2-dichloroethane (19.5 mL) and trifluoroacetic acid (0.5 mL). The tube was sealed with a screw-on cap (Teflon) and the reaction mixture was refluxed at 85 °C for 2.5 h. After the reaction mixture was cooled to room temperature, the mixture was diluted with dichloromethane and washed with saturated aqueous sodium bicarbonate (2 × 30 mL). The organic phase was

dried over Na_2SO_4 and concentrated under reduced pressure. The crude product was purified with flash chromatography (neutral silica gel, hexane: dichloromethane = 3:7) to give pure **2** as a light brown solid (485 mg, 70%). ^1H NMR (500 MHz, $CDCl_3$): δ 8.09 (d, *J* = 8.0 Hz, 1H), 7.69 (td, *J* = 7.5 Hz, 1.0 Hz, 1H), 7.55-7.39 (m, 5H), 7.24-7.22 (m, 2H), 3.55 (s, 2H), 3.50 (t, *J* = 7.0 Hz, 2H), 2.75 (t, *J* = 7.0 Hz, 2H); $^{13}C\{^1H\}$ NMR (125 MHz, $CDCl_3$): δ 208.8, 157.8, 146.7, 146.6, 135.4, 129.3, 129.2, 128.81, 128.77, 128.4, 126.8, 126.3, 126.2, 123.6, 42.8, 37.6, 32.4. HRMS (FAB) m/z: calcd. $C_{19}H_{16}NO$ $[M+H]^+$: 274.1232, found: 274.1235.

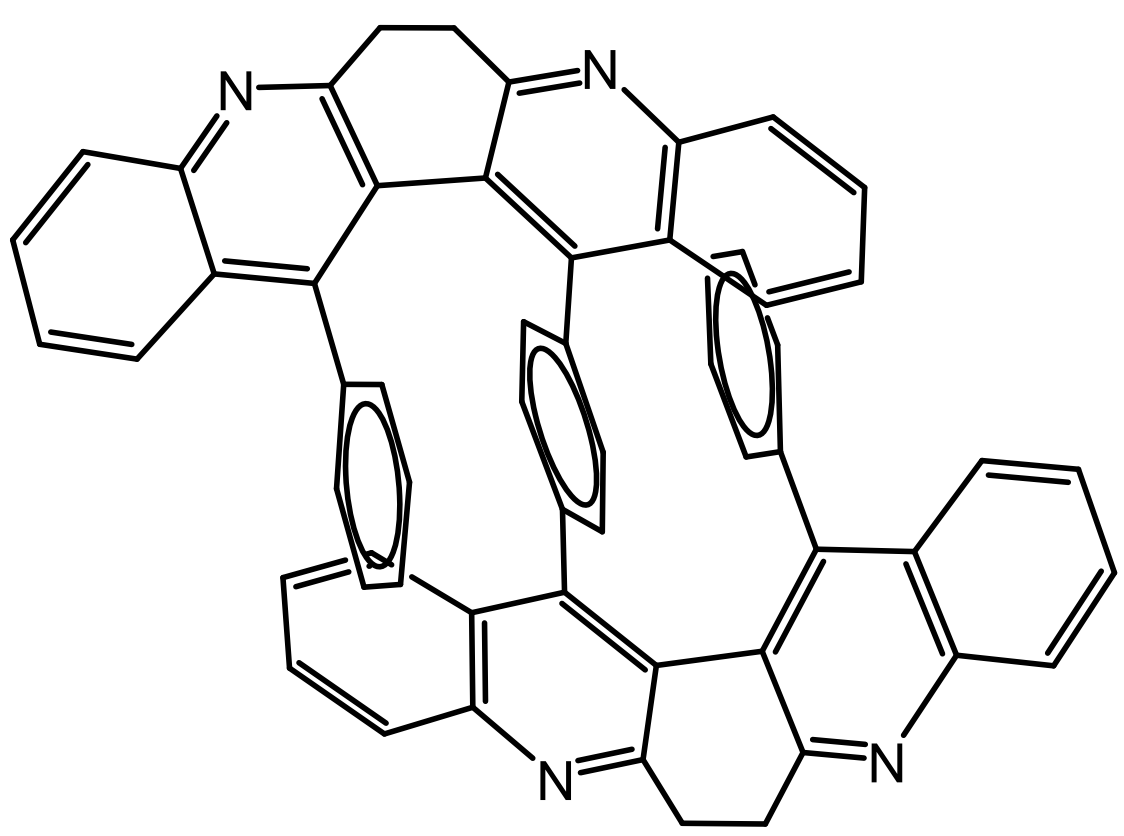

3

Compound **1** (52 mg, 0.1 mmol, 1 equiv) and **2** (68 mg, 0.25 mmol, 2.5 equiv) were placed in a pressurized tube. 1,2-Dichloroethane (2 mL) and trifluoroacetic acid (0.15 mL) were added to dissolve the solid. The tube was then sealed with a screw-on cap (Teflon). The reaction mixture was refluxed

(85 °C) for 4 h and diluted with dichloromethane (20 mL). The mixture was washed with saturated aqueous sodium bicarbonate (2 × 30 mL) and the organic layer was dried over Na_2SO_4. After the solvent was removed under reduced pressure, methanol (14 mL) was added to the remaining solid and the suspension was centrifuged for 5 min (6000 rpm). Compound **3** was obtained as a white solid (23.7 mg, 30%).

^{1}H NMR (500 MHz, $CDCl_3$): δ 8.17 (d, *J* = 8.0 Hz, 2H from quinoline units), 8.13 (d, *J* = 8.0 Hz, 2H from quinoline units), 7.78 (t, *J* = 7.0 Hz, 2H from quinoline units), 7.74 (d, *J* = 8.0 Hz, 2H from quinoline units), 7.67 (t, *J* = 7.0 Hz, 2H from quinoline units), 7.40 (t, *J* = 7.0 Hz, 2H from quinoline units), 7.35 (d, *J* = 8.0 Hz, 2H from quinoline units), 7.05-6.97 (m, 4H, two from quinoline units, two from the outer phenyl units), 6.83 (br s, 4H from the outer phenyl units), 6.60-6.28 (m, 6H from outer phenyl and inner phenylene units), 6.02 (br s, 2H from the inner phenylene), 3.58-3.37 (m, 8H); ^{13}C{^{1}H} NMR (125 MHz, $CDCl_3$): δ 162.4, 162.3, 147.2, 147.1, 145.6, 135.0, 131.8, 131.0, 129.7, 129.57, 129.5, 129.1, 128.9, 128.2, 127.7, 126.1, 125.9, 125.8, 125.7, 125.7, 125.3, 125.1, 124.9, 34.8. HRMS (MALDI) m/z: calcd for $C_{58}H_{39}N_4$ [M+H]$^+$: 791.3175, found: 791.3174

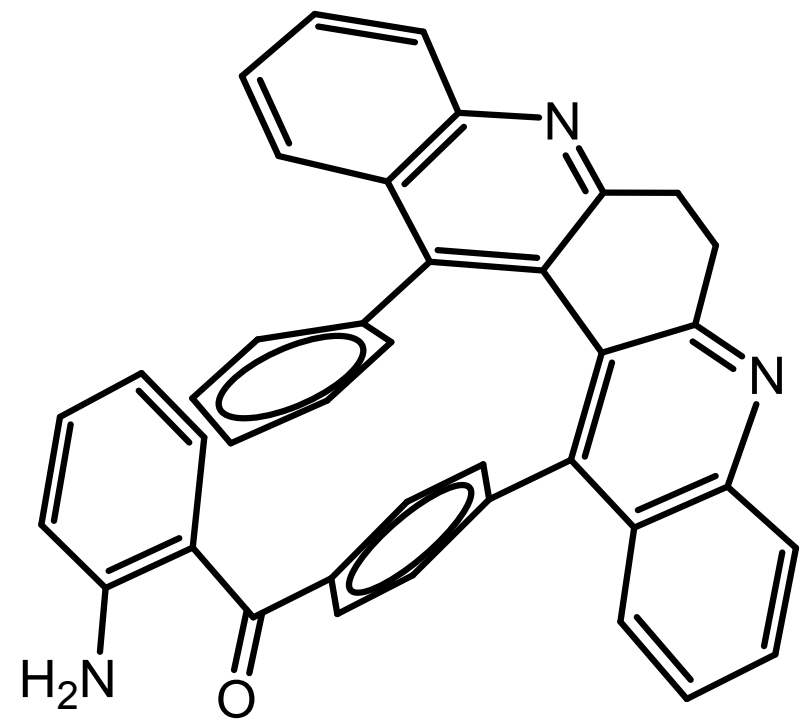

4

Compound **1** (516 mg, 1 mmol, 1.3 equiv) and compound **2** (210 mg, 0.77 mmol, 1 equiv) were dissolved in 1,2-dichloroethane (1.2 mL) and trifluoroacetic acid (0.1 mL) in a pressurized tube. After the tube was sealed with a screw-on cap (Teflon), the reaction mixture was heated to 85 °C for 2.5 h. The mixture was diluted with dichloromethane (15 mL) and washed with saturated aqueous sodium bicarbonate (2 × 30 mL). The organic layer was dried over Na_2SO_4 before the solution was concentrated under reduced pressure. The crude product was purified with flash chromatography (neutral silica gel, dichloromethane: ethyl acetate = 9:1) to give **4** as a yellow solid (149 mg, 35%). ^{1}H NMR (500 MHz, $CDCl_3$): δ 8.11 (d, *J* = 8.0 Hz, 1H), 8.08 (d, *J* = 8.0 Hz, 1H), 7.73-7.66 (m, 4H), 7.42 (d, *J* = 8.0 Hz, 1H), 7.40-7.29 (m, 4H), 7.25 (t, *J* = 7.0 Hz, 2H), 7.12 (br, 2H), 6.72 (d, *J* = 8.0 Hz, 1H), 6.70-6.36 (br m, 5H, overlapping signals), 6.12 (s, 2H), 3.49-3.30 (m, 4H); ^{13}C{^{1}H} NMR (125 MHz, CDCl3): δ 198.3, 162.5, 162.3, 151.2, 151.0, 147.2, 147.0, 146.3, 145.3, 139.0, 138.3, 135.4, 134.4, 134.2, 131.4, 129.7, 129.7, 129.0, 128.9, 128.8, 128.5, 127.8,

127.7, 127.5, 126.0, 125.8, 125.7, 125.5, 125.1, 124.6, 117.9, 117.1, 115.5, 34.9, 34.9. HRMS (MALDI) m/z: calcd for $C_{39}H_{28}N_3O$ $[M+H]^+$: 554.2232, found: 554.2233.

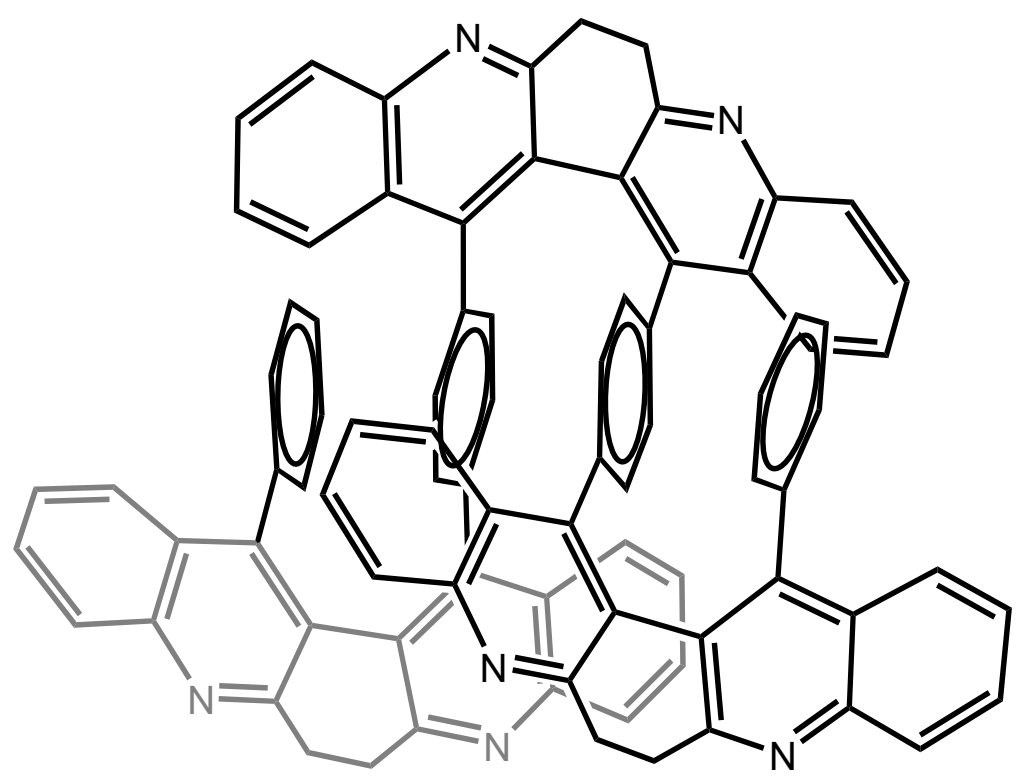

5

In a pressurized tube, compound **4** (138 mg, 0.25 mmol, 2.5 equiv) and 1,4-cyclohexadione (11.2 mg, 0.1 mmol, 1 equiv) were dissolved in 1,2-dichloroethane (1.9 mL) and trifluoroacetic acid (0.15 mL). The condensation reaction was heated at 85 °C for 6 h before the reaction mixture was diluted with dichloromethane (20 mL). The mixture was washed with saturated aqueous sodium bicarbonate (2 × 30 mL). The organic phase was dried over Na_2SO_4 before the solution was concentrated under reduced pressure. Methanol (14 mL) was added to the residual solid and the suspension was centrifuged (6000 rpm) for 5 min. Pure compound **5** (23.7 mg, 25%) was obtained as a white

solid after decanting the supernatant.

^{1}H NMR (500 MHz, $CDCl_3$) : δ 8.21 (d, *J* = 8.0 Hz, 2H from quinoline units), 8.01 (t, *J* = 7.0 Hz, 4H from quinoline units), 7.79 (t, *J* = 7.0 Hz, 2H from quinoline units), 7.65 (t, *J* = 7.0 Hz, 2H from quinoline units), 7.58 (d, *J* = 8.0 Hz, 2H from quinoline units), 7.52 (t, *J* = 7.0 Hz, 2H from quinoline units), 7.46 (d, *J* = 8.0 Hz, 2H from quinoline units), 7.28 (t, *J* = 7.0 Hz, 2H from quinoline units), 7.19 (t, *J* = 7.0 Hz, 2H from quinoline units), 6.93 (t, *J* = 7.0 Hz, 2H from quinoline units), 6.85 (d, *J* = 8.0 Hz, 2H from quinoline units), 6.74 (br s, 4H from outer phenyl units), 6.58 (t, *J* = 7.0 Hz, 2H from outer phenyl units), 6.50-6.10 (br m, 8H, overlapping signals from outer phenyl and inner phenylene units), 6.01 (d, *J* = 8.0 Hz, 2H from inner phenylene units), 5.80 (d, *J* = 8.0 Hz, 2H from inner phenylene units), 3.59-3.46 (m, 4H), 3.44-3.33 (m, 4H), 3.31-3.20 (m, 4H); ^{13}C{^{1}H} NMR (125 MHz, $CDCl_3$): δ 162.4, 162.3, 162.2, 147.1, 147.0, 146.1, 145.9, 144.7, 135.5, 135.1, 134.1, 132.4, 132.0, 131.3, 129.9, 129.6, 129.5, 129.3, 129.1, 128.8, 128.2, 127.4, 126.2, 125.83, 125.78, 125.68, 125.63, 125.2, 124.9, 124.6, 124.5, 124.3, 34.82, 34.69, 34.56. HRMS (FAB) m/z: calcd for $C_{84}H_{55}N_6$ $[M+H]^+$: 1147.4488, found: 1147.4483

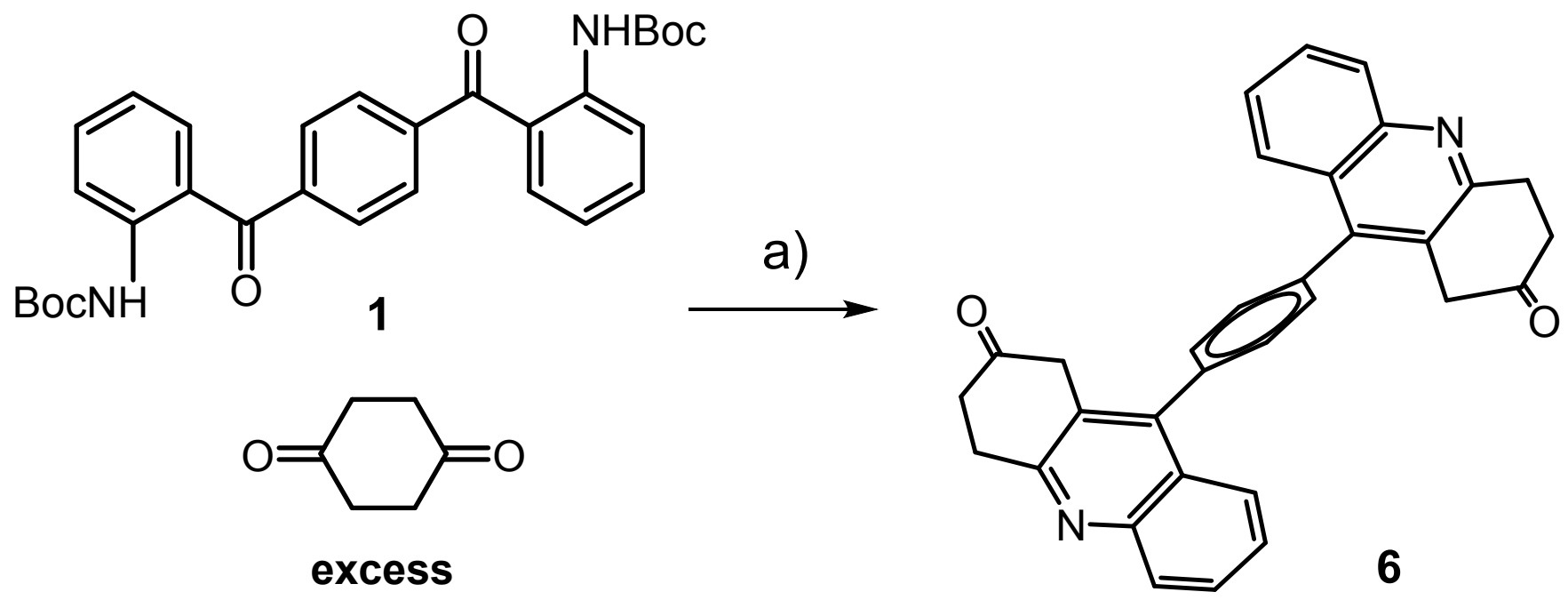

a) compound **1**:1,4-cyclohexadione = 1:3, 8% trifluoroacetic acid in refluxing 1,2-dichloroethane, 2.5 h, 10%

Scheme S1. Synthesis of central unit **6** for constructing five-layer phenylene architecture **7**

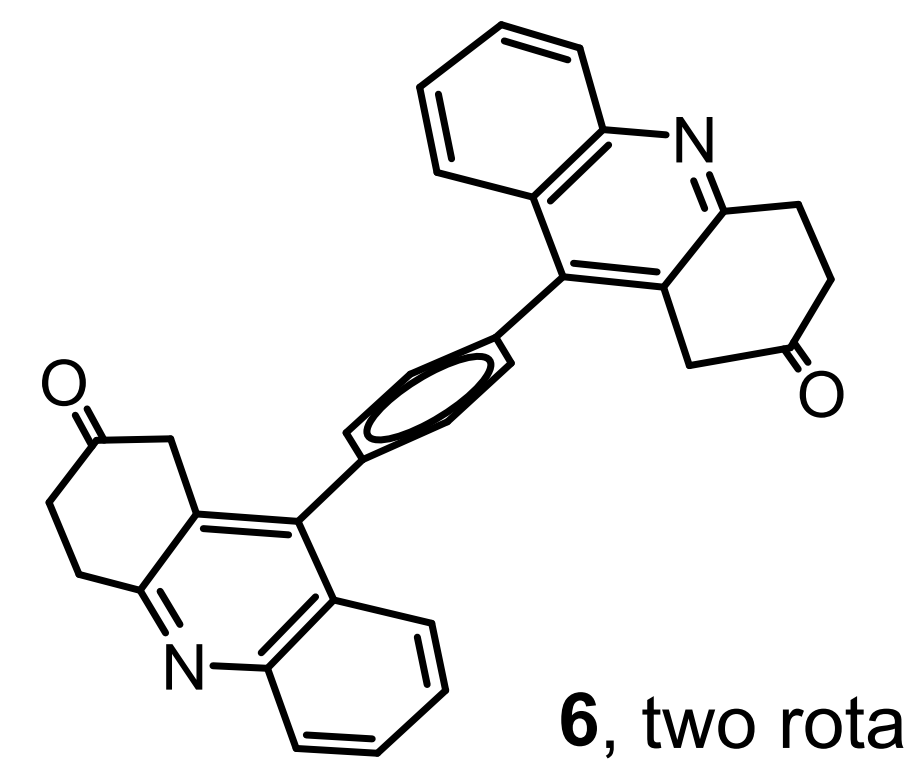

6, two rotamers

In a pressurized tube, compound **1** (516 mg, 1 mmol, 1 equiv) and 1,4-cyclohexadione (336 mg, 3 mmol, 3 equiv) were dissolved in anhydrous 1,2-dichloroethane (19.6 mL) and trifluoroacetic acid (1.5 mL). After the tube was sealed with a screw-on cap (Teflon), the reaction mixture was heated to 85 °C for 2.5 h. The mixture was diluted with dichloromethane (50 mL) and washed with saturated aqueous sodium bicarbonate (2 × 30 mL). The organic layer

was dried over Na_2SO_4 and concentrated under reduced pressure. The crude product was purified with flash chromatography (neutral silica gel, hexane: dichloromethane = 1:9) to afford **6** as a brown solid (140 mg, 20%).

^1H NMR (500 MHz, $CDCl_3$): δ 8.18-8.11 (m, 2H), 7.78-7.71 (m, 4H), 7.57-7.49 (m, 4H), 7.44 (s, 1.5H), 7.43 (s, 2.5H), 3.68 (s, 2.5H), 3.66 (s, 1.5H), 3.55 (t, 4H, *J* = 7.0 Hz), 2.89-2.74 (m, 4H) (noninteger integrations arise due to the presence of rotamers); $^{13}C\{^1H\}$ NMR (125 MHz, $CDCl_3$): δ 208.7, 208.1, 157.9, 157.6, 146.4, 146.0, 135.7, 129.9, 129.8, 129.7, 129.5, 128.9, 128.7, 126.8, 126.7, 126.6, 126.0, 125.9, 123.8, 123.6, 42.9, 37.6, 37.4, 32.2. HRMS (FAB) m/z: calcd for $C_{32}H_{25}N_2O_2$ $[M+H]^+$: 469.1916, found: 469.1912.

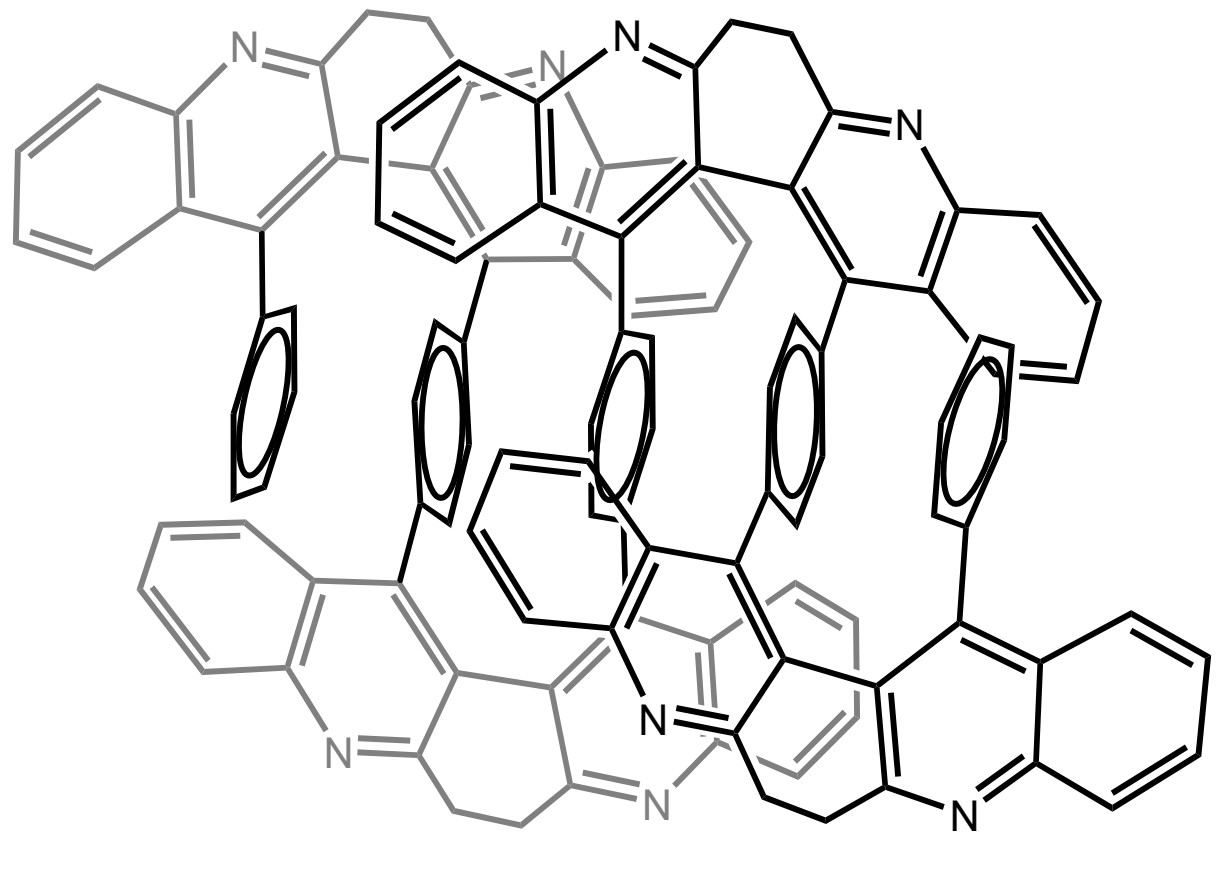

7

In a pressurized tube were placed compound **6** (93 mg, 0.2 mmol, 1 equiv) and compound **4** (276 mg, 0.5 mmol, 2.5 equiv). To the vessel were then added

anhydrous 1,2-dichloroethane (9.8 mL) and trifluoroacetic acid (0.75 mL). The tube was sealed with a screw-on cap (Teflon) and heated at 85 °C for 8 h. The reaction mixture was diluted with dichloromethane (20 mL) and washed with saturated aqueous sodium bicarbonate (2 × 30 mL). The organic layer was then dried over Na_2SO_4 and concentrated under reduced pressure. The crude solid product was suspended in ethyl acetate (14 mL) and the suspension was centrifuged (6000 rpm) for 5 min. The resulting yellow precipitate was suspended in toluene (14 mL) and the suspension was centrifuged (6000 rpm) again for another 5 min. Compound **7** was collected as a white solid (54 mg, 18%) after decanting the supernatant.

^{1}H NMR (500 MHz, $CDCl_3$): δ 8.10 (d, *J* = 8.0 Hz, 2H from quinoline units), 8.04 (d, *J* = 8.0 Hz, 2H from quinoline units), 8.01 (d, *J* = 8.0 Hz, 2H from quinoline units), 8.00 (d, *J* = 8.0 Hz, 2H from quinoline units), 7.70-7.62 (m, 6H from quinoline units), 7.56 (d, *J* = 8.0 Hz, 2H from quinoline units), 7.50 (t, *J* = 7.0 Hz, 2H from quinoline units), 7.24-7.35 (m, 4H from quinoline units), 7.06 (t, *J* = 7.0 Hz, 2H from quinoline units), 6.98 (d, *J* = 8.0 Hz, 2H from quinoline units), 6.89 (t, *J* = 7.0 Hz, 2H from quinoline units), 6.82-6.75 (m, 4H from quinoline units), 6.69 (br m, 4H from outer phenyl units), 6.53 (t, *J* = 7.0 Hz, 2H from outer phenyl units), 6.33 (br m, 4H from outer phenyl units), 6.15 (d, *J* = 8.0 Hz, 2H

from inner phenylene units), 6.04 (m, 4H from inner phenylene units), 5.85-5.78 (m, 4H from inner phenylene units), 5.75 (d, *J* = 8.0 Hz, 2H from inner phenylene units), 3.49-3.15 (m, 16H), Due to the low solubility of **7,** some minor signals from unidentified impurity were observed in 2-2.5 ppm and 4-4.5 ppm range. $^{13}C\{^{1}H\}$ NMR (125 MHz, $CDCl_3$): δ 162.36, 162.25, 162.12, 161.99, 147.11, 147.08, 146.98, 146.2, 145.8, 144.7, 135.3, 134.7, 134.3, 132.8, 132.1, 131.8, 131.5, 131.2, 129.9, 129.6, 129.5, 129.4, 129.2, 129.1, 128.7, 128.1, 127.4, 126.1, 126.0, 125.9, 125.8, 125.71, 125.67, 125.59, 125.49, 125.3, 125.2, 125.1, 124.9, 124.6, 124.5, 124.2, 124.1, 34.85, 34.68, 34.52, 34.42. HRMS (MALDI) m/z: calcd for $C_{110}H_{71}N_8$ $[M+H]^+$: 1503.5802, found: 1503.5810. Additional signals corresponding to $[M-1]^+$ and $[M-2]^+$ are also observed. These signals arise from dehydrogenated compound **7** product and its protonated form. The dehydrogenation likely occurred during the ionization process on one of the ethylene bridges.

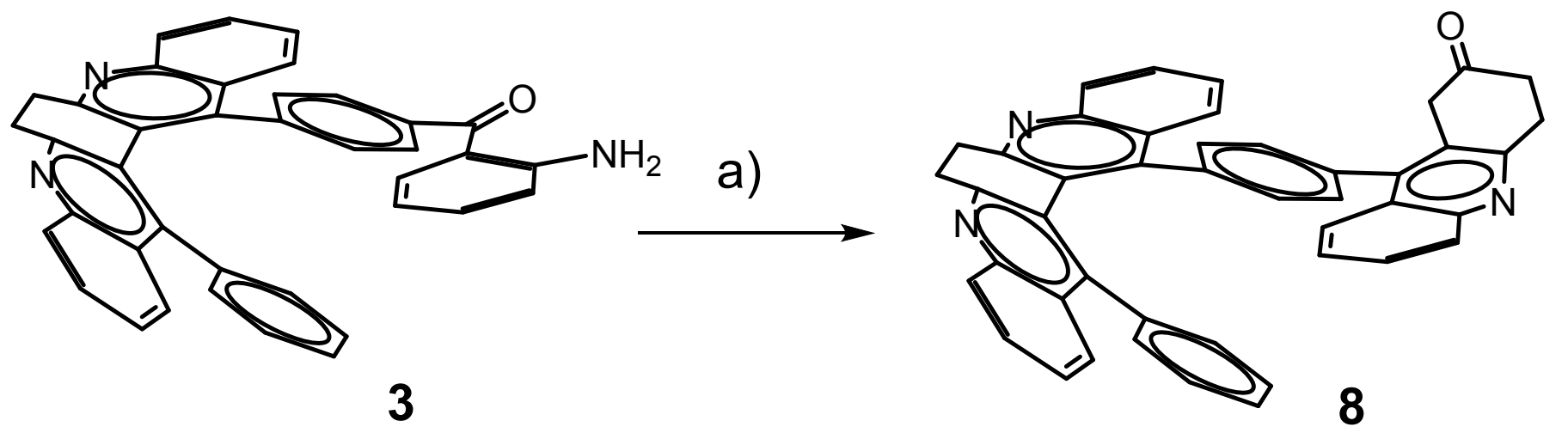

a) compound **3:**1,4**-**cyclohexadione = 1:3, 8% trifluoroacetic acid in refluxing 1,2-dichloroethane, 2.5 h

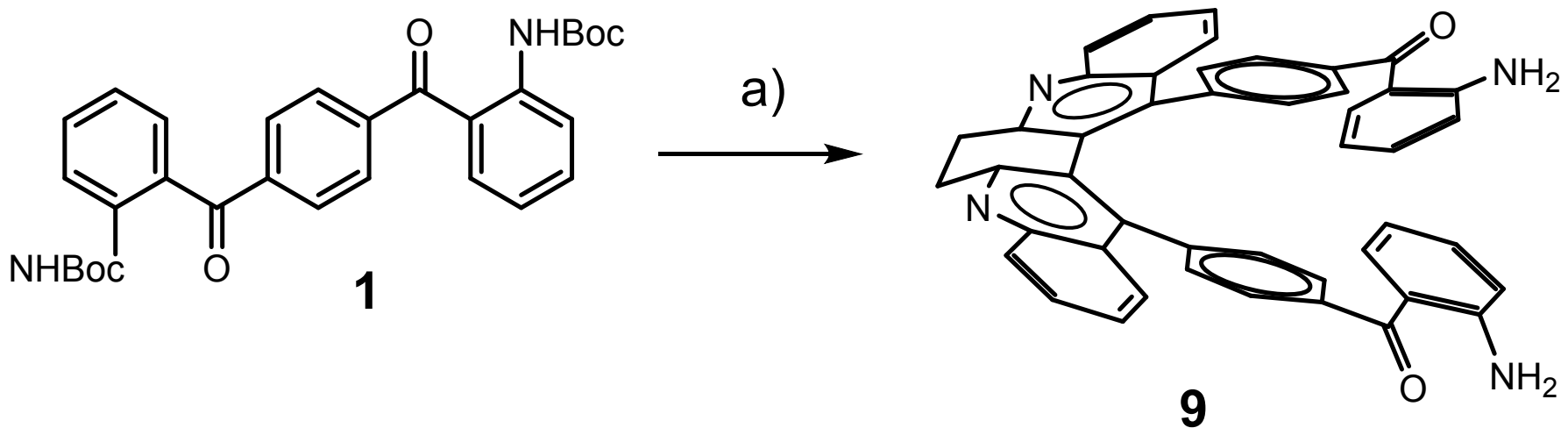

a) compound **1**:1,4-cyclohexadione = 2.5:1, 8% trifluoroacetic acid in refluxing 1,2-dichloroethane, 2.5 h

Scheme S2. Synthesis of compounds **8** and **9**, key intermediates en route to a six-layer phenylene stack

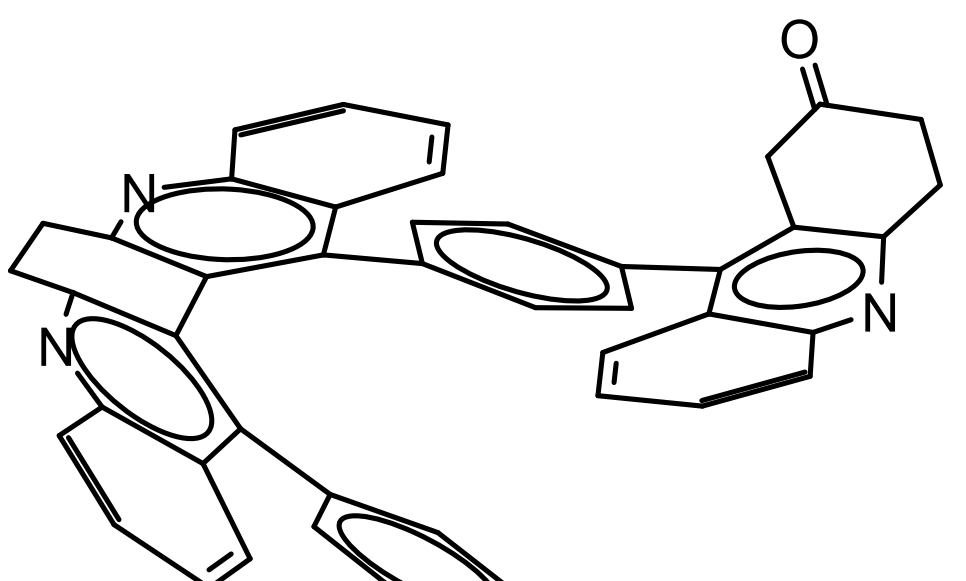

8

In a pressurized tube, compound **4** (276 mg, 0.5 mmol, 1 equiv) and 1,4-cyclohexadione (168 mg, 1.5 mmol, 3 equiv) were dissolved in anhydrous 1,2-dichloroethane (9.8 mL) and trifluoroacetic acid (0.75 mL). The vessel was sealed with a screw-on cap (Teflon) and the reaction mixture was heated at 85

°C for 2.5 h. The reaction mixture was diluted with dichloromethane (20 mL) and washed with saturated aqueous sodium bicarbonate (2 × 30 mL). The organic layer was dried over Na_2SO_4 before the solution was concentrated under reduced pressure. The crude product was purified with flash chromatography (neutral silica gel, dichloromethane: ethyl acetate = 4:6) to give condensation product **8** as a brown solid (63 mg, 20%).

^{1}H NMR (500 MHz, $CDCl_3$): δ 8.13 (dd, *J* = 8.0 Hz, 1H), 8.10 (m, 2H), 7.86 (dd, *J* = 8.0 Hz, 1H), 7.73 (t, *J* = 7.0 Hz, 2H), 7.70 (t, *J* = 7.0 Hz, 2H), 7.41 (m, 4H), 7.24-7.16 (br, 2H), 7.00 (br, 2H), 6.83-6.45 (br, 4H), 3.61 (br, 1H), 3.50 (t, *J* = 7.0 Hz, 2H), 3.43 (m, 4H), 2.75 (t, *J* = 7.0 Hz, 2H); ^{13}C{^{1}H} NMR (125 MHz, $CDCl_3$): δ 208.4, 162.5, 157.9, 147.3, 147.11, 146.7, 146.2, 145.7, 145.6, 135.9, 135.5, 134.7, 131.8, 129.8, 129.3, 129.1, 129.0, 128.4, 126.6, 126.4, 126.2, 125.9, 125.8, 125.7, 125.2, 124.9, 123.5, 43.0, 37.4, 35.0 , 34.9, 32.4. HRMS (MALDI) m/z: calcd for $C_{45}H_{32}N_3O$ [M^+]: 630.2545, found: 630.2523.

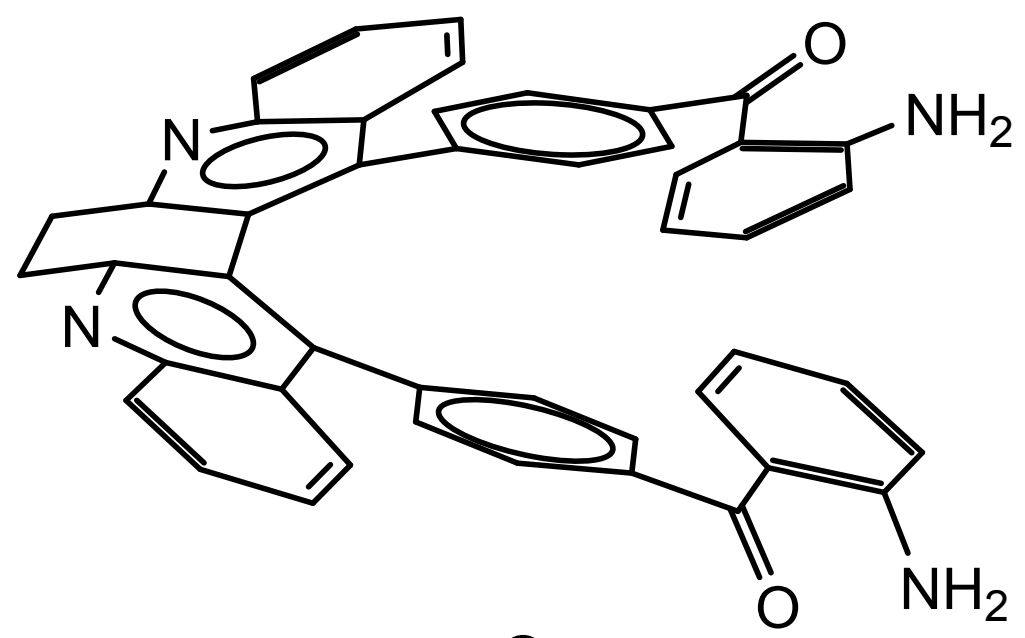

9

Compound **1** (645 mg, 1.25 mmol, 2.5 equiv) and 1,4-cyclohexadione (56 mg, 0.5 mmol, 1 equiv) were dissolved in 1,2-dichloroethane (9.8 mL) and trifluoroacetic acid (0.75 mL) in a pressurized tube. The reaction vessel was sealed with a screw-on cap (Teflon) and heated for 2.5 h at 85 °C. The reaction mixture was diluted with dichloromethane (20 mL) and washed with saturated aqueous sodium bicarbonate (2 × 30 mL). The organic layer was dried over Na_2SO_4 and concentrated under reduced pressure. The crude product was purified with flash chromatography (neutral silica gel, dichloromethane: ethyl acetate = 3:1) to give diamine **9** as a yellow solid (218 mg, 33%).

^{1}H NMR (500 MHz, $CDCl_3$): δ 8.11 (d, *J* = 8.0 Hz, 2H), 7.75-7.69 (m, 4H), 7.56-7.26 (br m, 10H), 6.80-6.52 (br m, 8H), 6.12 (s, 4H), 3.42 (m, 4H); ^{13}C{^{1}H} NMR (125 MHz, $CDCl_3$): δ 198.1, 162.3, 151.1, 147.1, 145.4, 139.4, 138.1, 134.5, 134.2, 131.1, 130.0, 129.2, 128.9, 126.4, 125.8, 125.5, 124.8, 117.7, 117.2,

115.6, 34.8. HRMS (MALDI) m/z: calcd for $C_{46}H_{33}N_4O_2$ $[M+H]^+$: 673.2604, found: 673.2595.

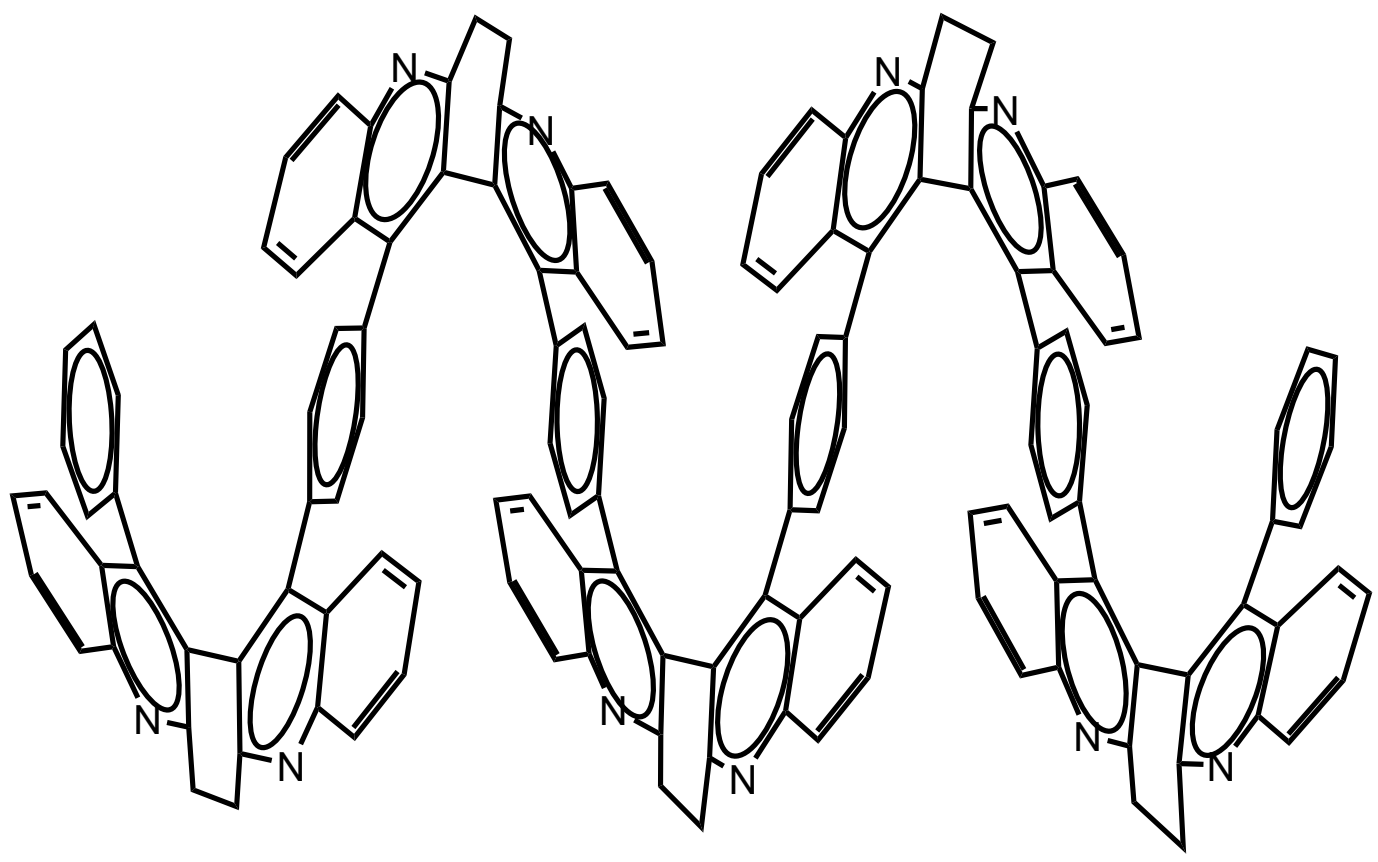

10

In a pressurized tube, compound **9** (27 mg, 0.04 mmol, 1 equiv) and **8** (64 mg, 0.1 mmol, 2.6 equiv) were dissolved in 1,2-dichloroethane (0.76 mL) and trifluoroacetic acid (0.1 mL). The tube was then sealed with a screw-on cap (Teflon) and heated at 85 °C for 10 h. After completion of the reaction, the mixture was diluted with dichloromethane (20 mL) and washed with saturated aqueous sodium bicarbonate (2 × 30 mL). The organic layer was dried over Na_2SO_4 before the solution was concentrated under reduced pressure. To the remaining crude product was added ethyl acetate (14 mL) and the suspension was centrifuged (6000 rpm) for 5 min. The yellow precipitate was collected and resuspended in toluene (14 mL). The suspension was again centrifuged (6000 rpm) for 5 min. Product **10** was obtained as a white solid (2 mg, 5%).

^{1}H NMR (500 MHz, $CDCl_3$): δ 8.11 (d, *J* = 7.0 Hz, 2H from quinoline units), 8.04 (d, *J* = 7.0 Hz, 2H from quinoline units), 8.00-7.93 (m, 6H from quinoline units), 7.70-7.44 (m, 14H from quinoline units), 7.06 (t, *J* = 7.0 Hz, 2H from quinoline units), 6.93 (d, *J* = 7.0 Hz, 2H from quinoline units), 6.87 (t, *J* = 7.0 Hz, 2H from quinoline units), 6.83-6.76 (m, 4H from quinoline units), 6.74-6.62 (m, 6H from quinoline units), 6.49 (t, *J* = 7.0 Hz, 2H from outer phentyl units), 6.44-6.15 (br m, 6H from outer phenyl units), 6.13 (d, J = 7 Hz, 2H from outer phenyl units), 6.00-5.51 (m, 16H from inner phenylene units), 3.43-3.13 (m, 20H); $^{13}C\{^{1}H\}$ NMR (125 MHz, $CDCl_3$): δ 162.37, 162.24, 162.11, 162.02, 161.94, 147.12, 147.07, 147.0, 146.1, 145.76 (×2), 145.1, 145.0, 144.7, 135.3, 135.0, 134.9, 134.4, 134.2, 132.8, 132.1, 132.0, 131.8, 131.6, 131.2, 129.9, 129.5, 129.3, 129.2, 129.1, 128.8, 128.1, 127.4, 126.1, 125.9, 125.76, 125.68, 125.56, 125.3, 125.2, 125.1, 124.9, 124.6, 124.5, 124.21, 124.16, 124.0, 34.84, 34.67, 34.54, 34.39. HRMS (FAB) m/z: calcd for $C_{136}H_{87}N_{10}$ $[M+H]^+$: 1859.7115, found: 1859.7120

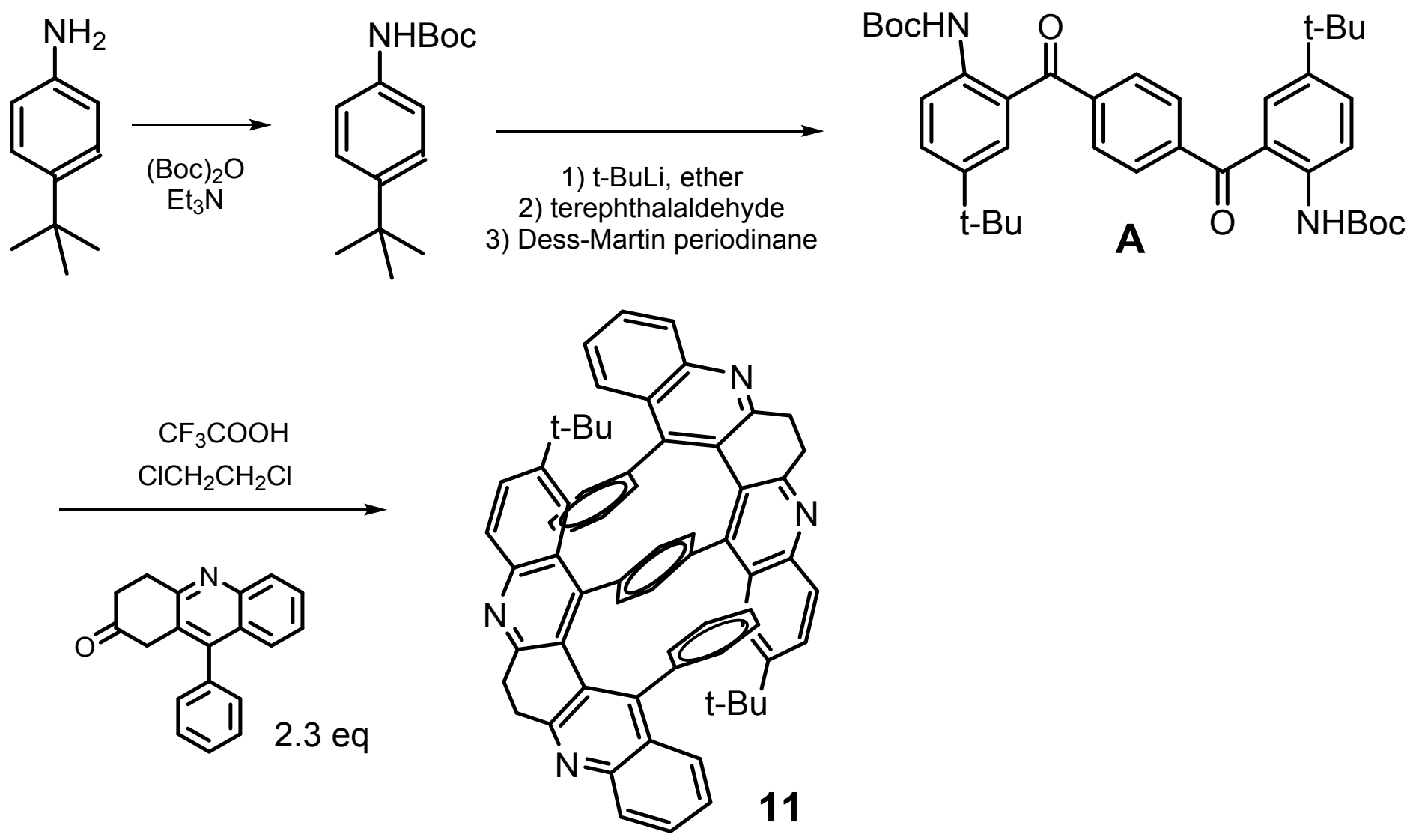

Scheme S3. Synthesis of non-aggregating three-layer compound **11**

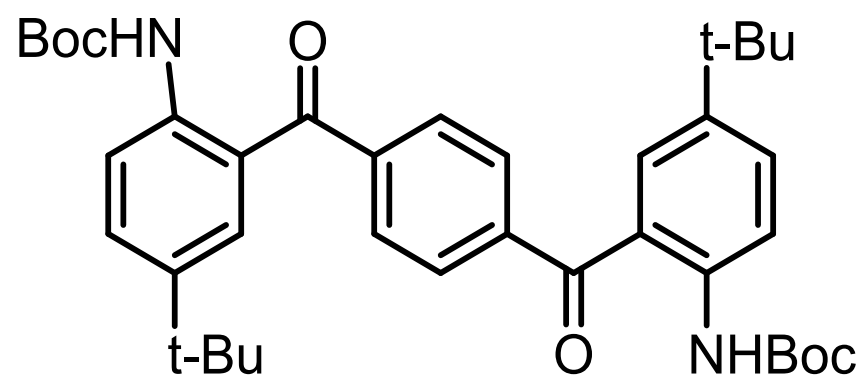

A

Compound **A** (di-t-butyl (terephthaloylbis(4-(t-butyl)-2,1-phenylene)) dicarbamate) was synthesized from t-butyl (4-(t-butyl)phenyl)carbamate and terephthalaldehyde using the same procedure as that used for **1.** The product was isolated as a light-yellow solid (32%).

^{1}H NMR (400 MHz, $CDCl_3$): δ 10.00 (s, 2H), 8.36 (d, *J* = 7.6 Hz, 2H), 7.83 (s, 4H), 7.62 (dd, *J* = 7.6 Hz, 2H), 7.52 (d, *J* = 7.6 Hz, 2H), 1.54 (s, 18H), 1.26 (s, 18H); ^{13}C{^{1}H} NMR (125 MHz, $CDCl_3$): δ 198.6, 153.1, 143.7, 142.0, 139.1, 131.9, 130.2, 129.6, 121.9, 119.9, 80.6, 34.1, 31.1, 28.3. HRMS (MALDI) m/z:

calcd for $C_{38}H_{48}N_2O_6$ [M$^+$]: 628.3512; found: 628.3509.

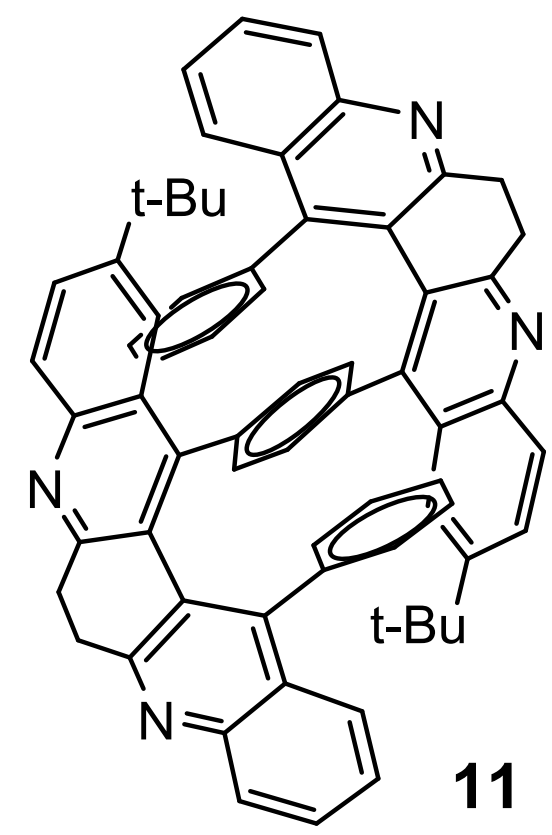

11

The compound was synthesized from **A** and **2** using an identical procedure to that for **3.** The compound was isolated as a white solid after centrifugation (55%). Single crystals of **11** were obtained from a saturated chloroform solution by slow evaporation of the solvent.

^{1}H NMR (400 MHz, $CDCl_3$): δ 8.13 (d, *J* = 8.1 Hz, 2H), 8.04 (d, *J* = 8.7 Hz, 2H), 7.74 (d, *J* = 8.7 Hz, 2H), 7.69 (t, *J* = 7.2 Hz, 2H), 7.51 (t, *J* = 7.2 Hz, 2H), 7.47 (d, *J* = 1.6 Hz, 2H), 7.27 (d, *J* = 7.2 Hz, 2H), 7.07 (t, *J* = 7.2 Hz, 2H), 6.91 (t, *J* = 7.6 Hz, 2H), 6.84 (t, *J* = 7.6 Hz, 2H), 6.57 (br s, 2H), 6.46 (d, *J* = 7.6 Hz, 2H), 6.32 (d, *J* = 7.6 Hz, 2H), 6.05 (br s, 2H), 3.59-3.21 (m, 8H), 0.80 (s, 18H). $^{13}C\{^1H\}$ NMR (125 MHz, $CDCl_3$): δ 162.4, 161.8, 149.0, 147.33, 147.31, 145.6, 145.4, 135.4, 135.2, 132.0, 131.1, 131.0, 130.5, 129.7, 129.1, 128.5, 128.4, 128.3, 127.6, 126.4, 126.3, 126.28, 125.4, 124.9, 124.7, 120.1, 35.3, 34.8, 34.6, 30.5. HRMS (MALDI) m/z: calcd for $C_{66}H_{54}N_4$ [M+H]$^+$: 903.4427; found: 903.4431.

Additional signals corresponding to [M-1]+ and [M-2]+ are also observed. These signals arise from dehydrogenated compound **11** product and its protonated form. The dehydrogenation likely occurred during the ionization process on one of the ethylene bridges.

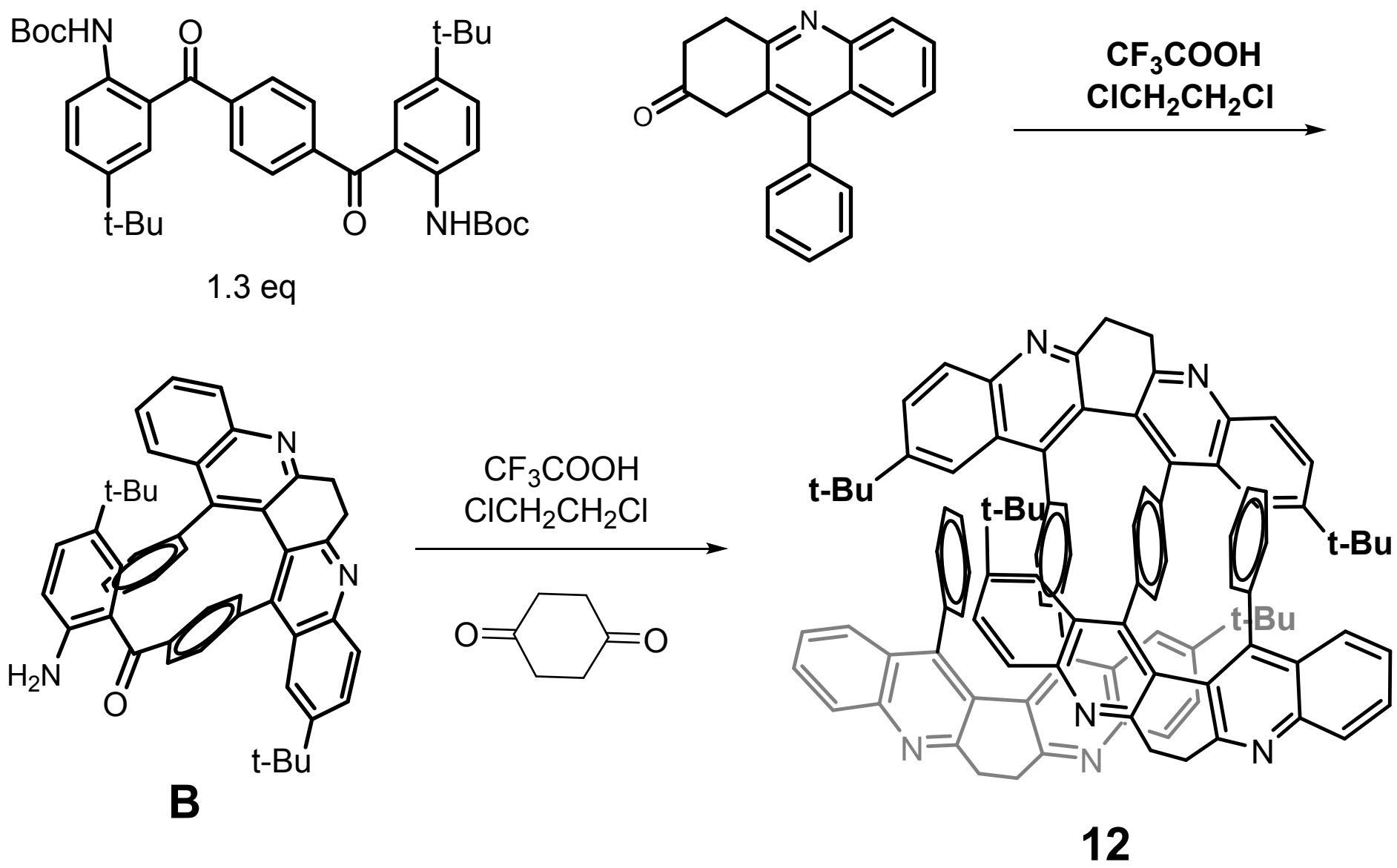

Scheme S4. Synthesis of non-aggregating four-layer compound **12**

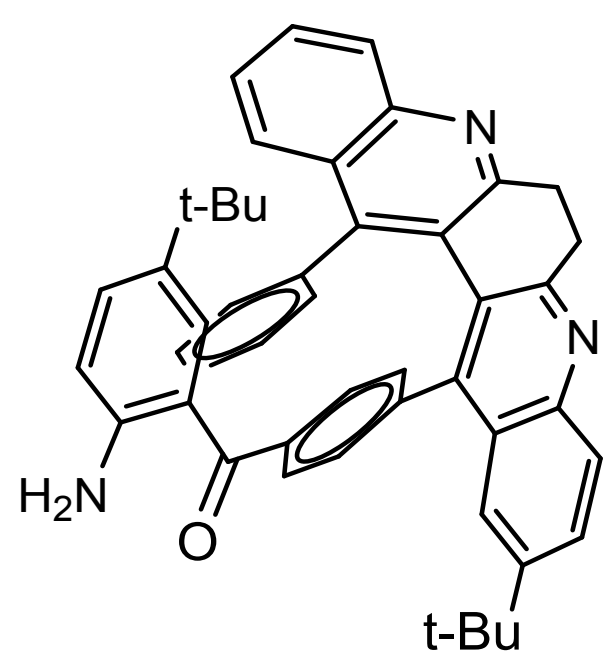

B

Compound **B** was synthesized by Friedländer condensation reaction of compound **A** (1.3 equiv) and compound **2** under conditions identical to those

used for the synthesis of **4.** After flash chromatography on (neutral silica gel, dichloromethane: ethyl acetate = 9:1), the product was isolated as a light yellow solid (45%).

^{1}H NMR (400 MHz, $CDCl_3$): δ 8.01 (m, 2H), 7.81 (dd, J = 8.0 Hz, 1H), 7.73-7.68 (m, 3H), 7.50 (br s, 1H), 7.43 (d, *J* = 7.6 Hz, 1H), 7.38-7.32 (m, 3H), 7.24 (t, *J* = 7.0 Hz, 1H), 7.13 (br, 2H), 6.85-6.41 (br m, 5H, overlapping signals), 6.00 (s, 2H), 3.57-3.26 (m, 4H), 1.29 (s, 9H), 1.09 (s, 9H); $^{13}C\{^1H\}$ NMR (125 MHz, $CDCl_3$): δ 198.2, 162.4, 161.8, 148.8, 148.7, 147.2, 146.3, 145.6, 145.1, 139.0, 138.7, 138.2, 135.5, 131.9, 131.4, 130.2, 129.7, 129.0, 128.8, 128.6, 127.7, 126.2, 125.9, 125.1, 120.9, 117.5, 117.0, 35.1, 34.9, 31.2. HRMS (MALDI) m/z: calcd for $C_{47}H_{44}N_3O$ $[M+H]^+$: 666.3484, found: 666.3488.

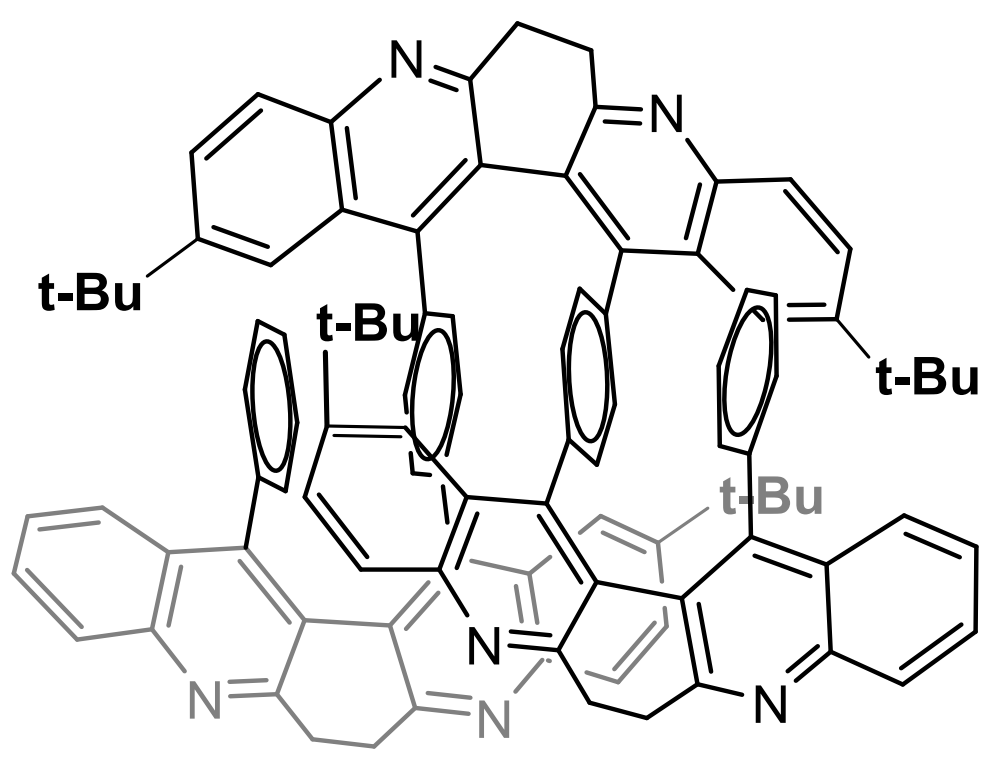

12

The four-layer compound **12** was synthesized from compound **B** and 1,4-cyclohexadione by employing the general Friedländer condensation procedure

for preparing **5**. Compound **12** was isolated as a colorless powder after centrifugation. Single crystals of **12** were obtained from a saturated 1,2-dichloroethane solution after slow evaporation of the solvent.

^{1}H NMR (400 MHz, $CDCl_3$): δ 8.06 (d, *J* = 7.6 Hz, 2H), 8.00 (d, *J* = 7.6 Hz, 2H), 7.98 (d, *J* = 7.6 Hz, 2H), 7.75 (d, *J* = 7.6 Hz, 2H), 7.62 (t, *J* = 7.0 Hz, 4H), 7.39 (d, *J* = 7.6 Hz, 2H), 7.37-7.26 (m, 6H, overlapping broad signals), 7.21 (t, *J* = 7.0 Hz, 2H), 6.80 (t, *J* = 7.0 Hz, 2H), 6.75 (t, *J* = 7.0 Hz, 2H), 6.55 (d, *J* = 7.6 Hz, 2H), 6.40 (d, *J* = 7.6 Hz, 2H), 6.29 (d, *J* = 7.6 Hz, 2H), 6.27 (d, *J* = 7.6 Hz, 2H), 6.05 (d, *J* = 7.6 Hz, 2H), 6.01 (d, *J* = 7.6 Hz, 2H), 3.57-3.10 (m, 12H), 0.69 (s, 18H), 0.66 (s, 18H); ^{13}C{^{1}H} NMR (125 MHz, $CDCl_3$): δ 162.3, 161.9, 161.6, 149.4, 148.5, 147.3, 146.2, 145.9, 145.5, 145.4, 144.9, 135.5, 135.49, 135.3, 131.9, 131.6, 131.3, 130.7, 130.2, 129.4, 128.9, 128.9, 128.5, 128.3, 128.1, 127.3, 126.5, 126.4, 125.8, 125.7, 125.6, 124.5, 124.3, 120.9, 119.2, 35.3, 35.2, 34.5, 34.4, 30.7, 30.6. HRMS (MALDI) m/z: calcd for $C_{100}H_{87}N_6$ $[M+H]^+$: 1371.6992, found: 1371.6996

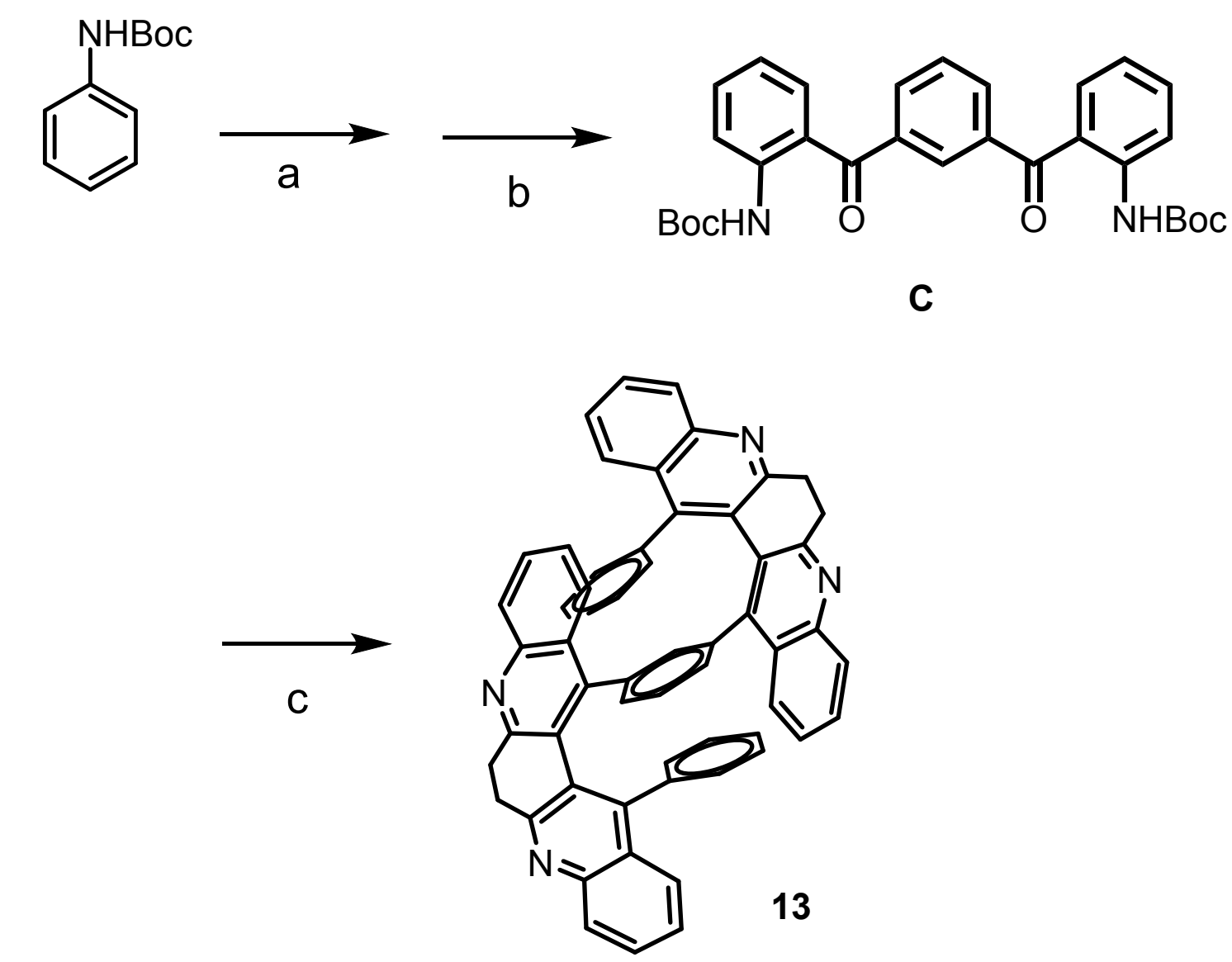

a) t-BuLi, ether, -78 to 0 ˚C; then isophthalaldehyde.

b) Dess-Martin periodinane, CH_2Cl_2, 31% over two steps

Scheme S5. Synthesis of m-phenylene-linked three-layer stack **13**

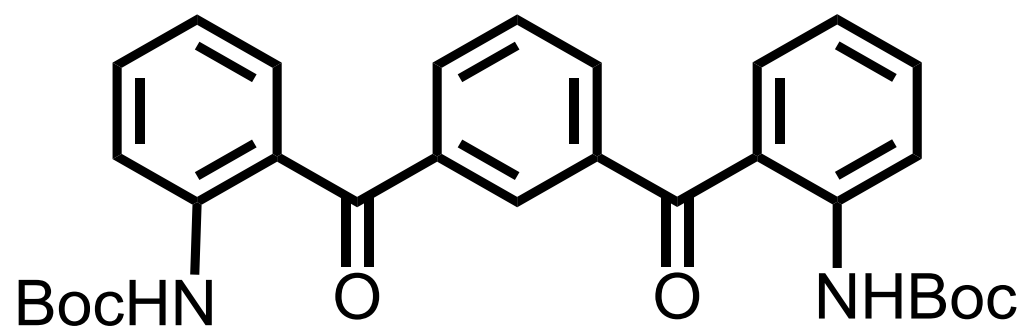

C

N-Boc-aniline (2.8 g, 14.3 mmol, 3 equiv) was dissolved in diethyl ether (60 mL) in a two-necked round-bottom flask. The solution was cooled to -78 ˚C under nitrogen. t-BuLi solution (20 mL, 1.9 M, 38 mmol, 8 equiv) was slowly added to the cold solution. After completion of the addition, the solution was warmed to 0 ˚C and stirred for 1.5 h. Isophthalaldehyde (0.64 g, 4.8 mmol in 60 mL of diethyl ether, 1 equiv) was added to the lithiated intermediate. The reaction was quenched with saturated aqueous NH_4Cl after 1.5 h. The organic layer

was dried over Na_2SO_4 and concentrated under reduced pressure. The crude diol product and Dess-Martin periodinane (10 g, 23.7 mmol, 2.5 equiv) were dissolved in dichloromethane (120 mL) at 0 °C. The reaction mixture was stirred at room temperature for 2.5 h before being quenched with saturated aqueous Na_2CO_3. The organic layer was dried over Na_2SO_4 and the solvent was removed under reduced pressure. The crude ketone was purified with silica gel flash chromatography (hexane: dichloromethane = 6:4). Compound **C** was isolated as a white solid (160 mg, 31%).

^{1}H NMR (500 MHz, $CDCl_3$) : δ 10.06 (s, 2H), 8.42 (d, *J* = 7.0 Hz, 2H), 7.96 (s, 1H), 7.88 (d, *J* = 7.0 Hz, 2H), 7.60 (t, *J* = 7.0 Hz, 1H), 7.55 (d, *J* = 7.0 Hz, 2H), 7.52 (d, *J* = 7.0 Hz, 2H), 7.00 (t, *J* = 7.0 Hz, 2H); ^{13}C{^{1}H} NMR (125 MHz, $CDCl_3$): δ 198.3, 152.9, 141.7, 139.1, 134.6, 133.4, 133.0, 130.8, 128.4, 122.1, 120.9, 120.1, 80.8, 28.3. HRMS (FAB) m/z: calcd for $C_{30}H_{33}N_2O_6$ $[M+H]^+$: 517.2339, found: 517.2334.

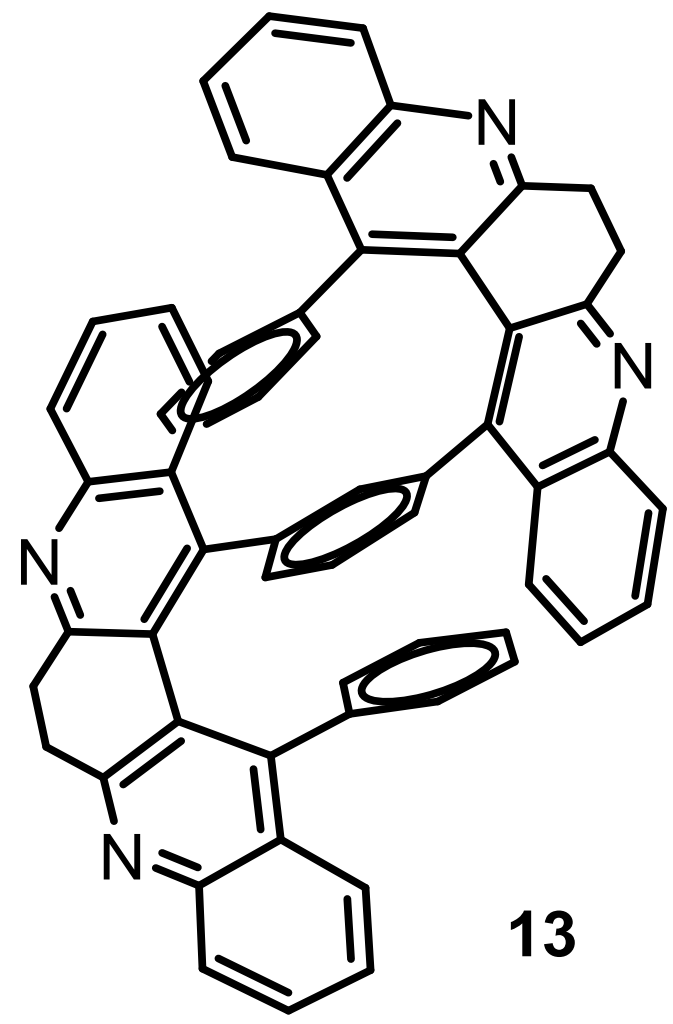

13

Compound **C** (108 mg, 0.21 mmol, 1 equiv), **2** (136 mg, 0.5 mmol, 2.4 equiv), 1,2-dichloroethane (3.8 mL), and trifluoroacetic acid (0.3 mL) were placed in a pressurized tube. The tube was sealed with a screw cap (Teflon) and the reaction mixture was refluxed for 4 h. After the reaction mixture was cooled to room temperature and diluted with CH_2Cl_2, the mixture was washed with saturated aqueous sodium bicarbonate (2 × 30 mL).

The organic layer was dried over Na_2SO_4 and concentrated under reduced pressure. The crude mixture was suspended in methanol (14 mL) and centrifuged at 6000 rpm for 5 min. Pure **13** was obtained as a white solid (49 mg, 31%). Single crystals of **13** were obtained from a saturated 1,2-dichloroethane solution by slow evaporation.

^{1}H NMR (500 MHz, $CDCl_3$): δ 8.27 (d, *J* = 8.0 Hz, 1H), 8.24 (d, *J* = 7.0 Hz, 1H), 8.08 (d, *J* = 7.0 Hz, 1H), 7.89 (d, *J* = 7.0 Hz, 1H), 7.87-7.79 (m, 3H), 7.76-7.73

(m, 2H), 7.54-7.49 (m, 2H), 7.41-7.22 (m, 3H), 7.11-7.00 (m, 2H), 6.82 (t, *J* = 7.0 Hz, 1H), 6.76-6.45 (m, 6H), 6.33 (d, *J* = 7.0 Hz, 2H), 6.18 (t, *J* = 7.0 Hz, 2H), 5.57 (s, 1H), 5.50-5.14 (m, 2H), 3.64-3.16 (m, 8H); $^{13}C\{^{1}H\}$ NMR (125 MHz, $CDCl_3$): δ 162.74, 162.66, 162.3, 161.9, 147.5, 147.18, 147.11, 146.57, 146.47, 146.3, 146.1, 136.2, 135.5, 135.0, 134.8, 133.3, 131.8, 131.6, 131.4, 130.4, 129.9, 129.8, 129.4, 129.3, 128.94, 128.78, 128.6, 128.4, 128.3, 127.8, 127.5, 127.2, 126.6, 126.6, 126.8, 126.3, 125.9, 125.8, 125.6, 125.5, 125.3, 125.1, 124.8, 123.7, 35.5, 35.1, 34.7. HRMS (MALDI) m/z: calcd for $C_{58}H_{39}N_4$ $[M+H]^+$: 791.3175, found: 791.3182

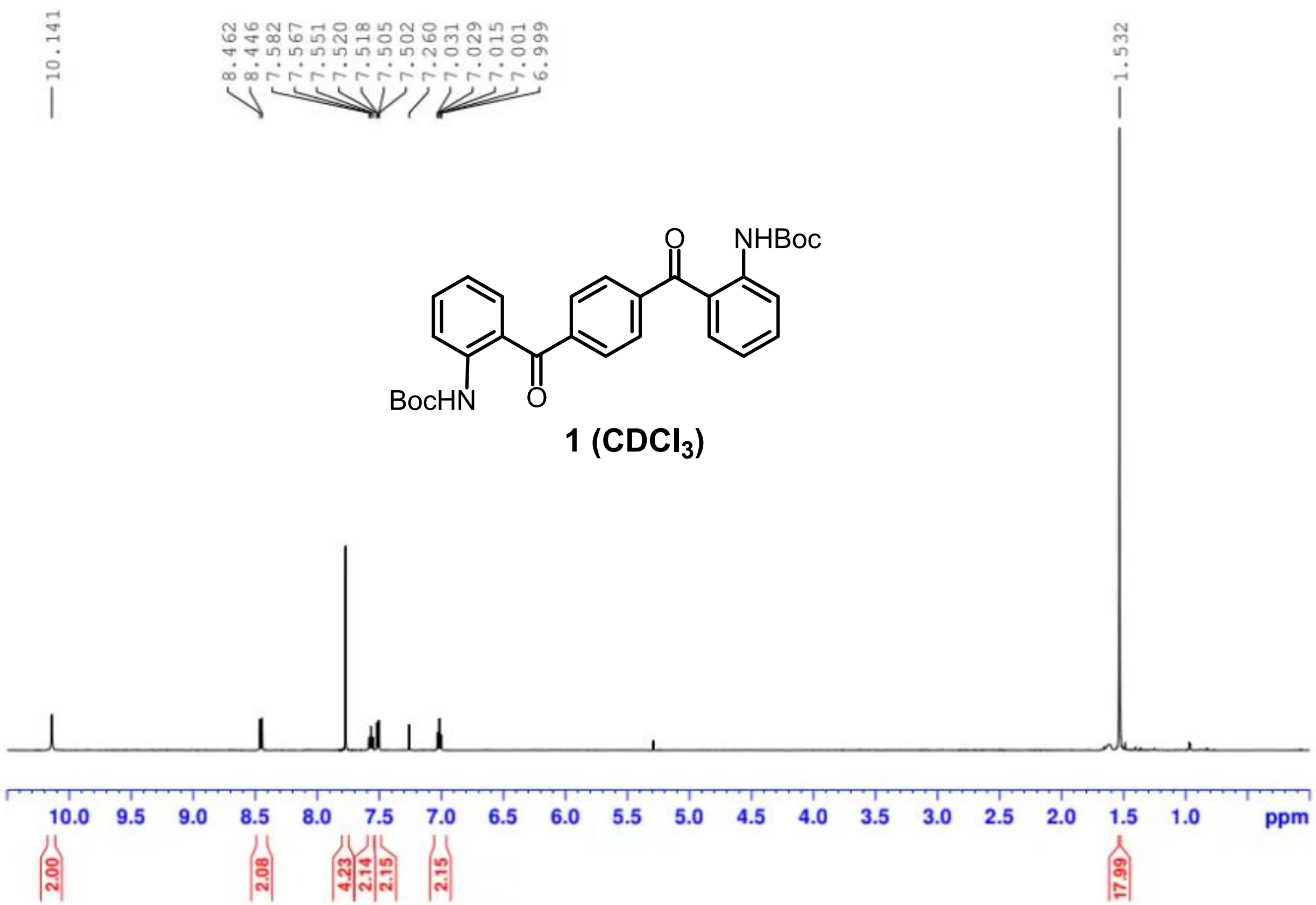

^{1}H NMR spectrum (500 MHz) of compound **1**

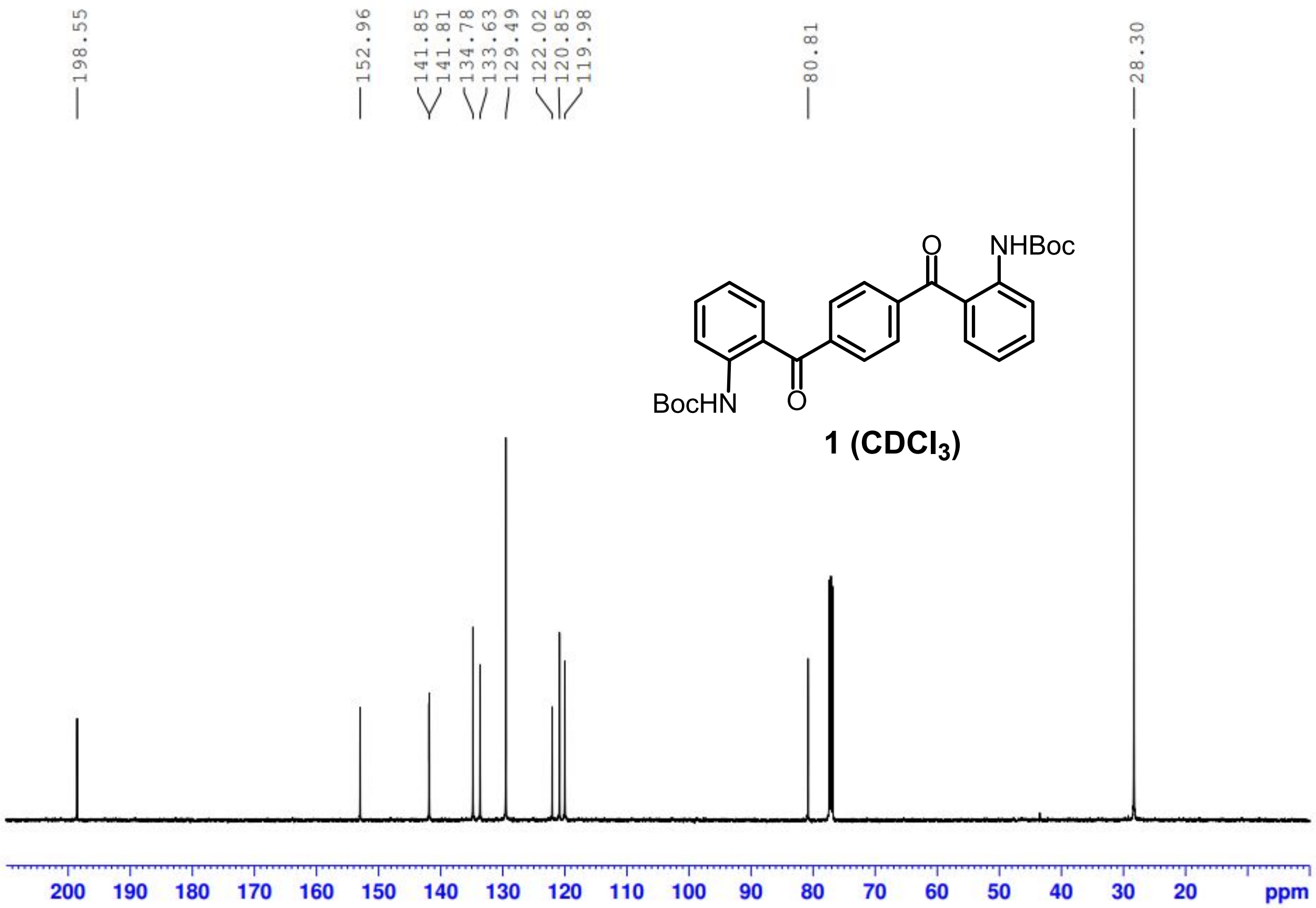

^{13}C NMR spectrum (125 MHz) of compound **1**

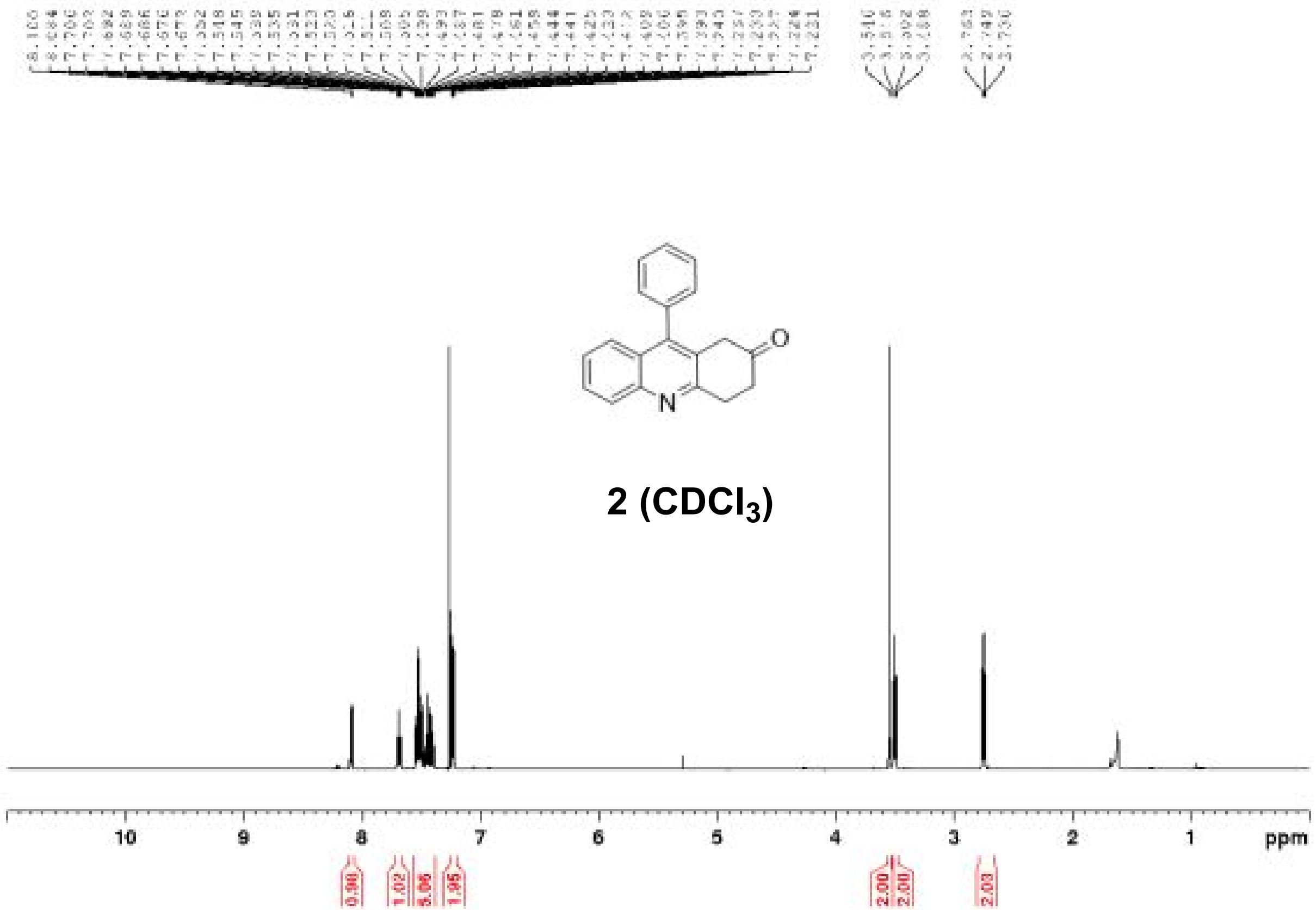

^{1}H NMR spectrum (500 MHz) of compound **2**

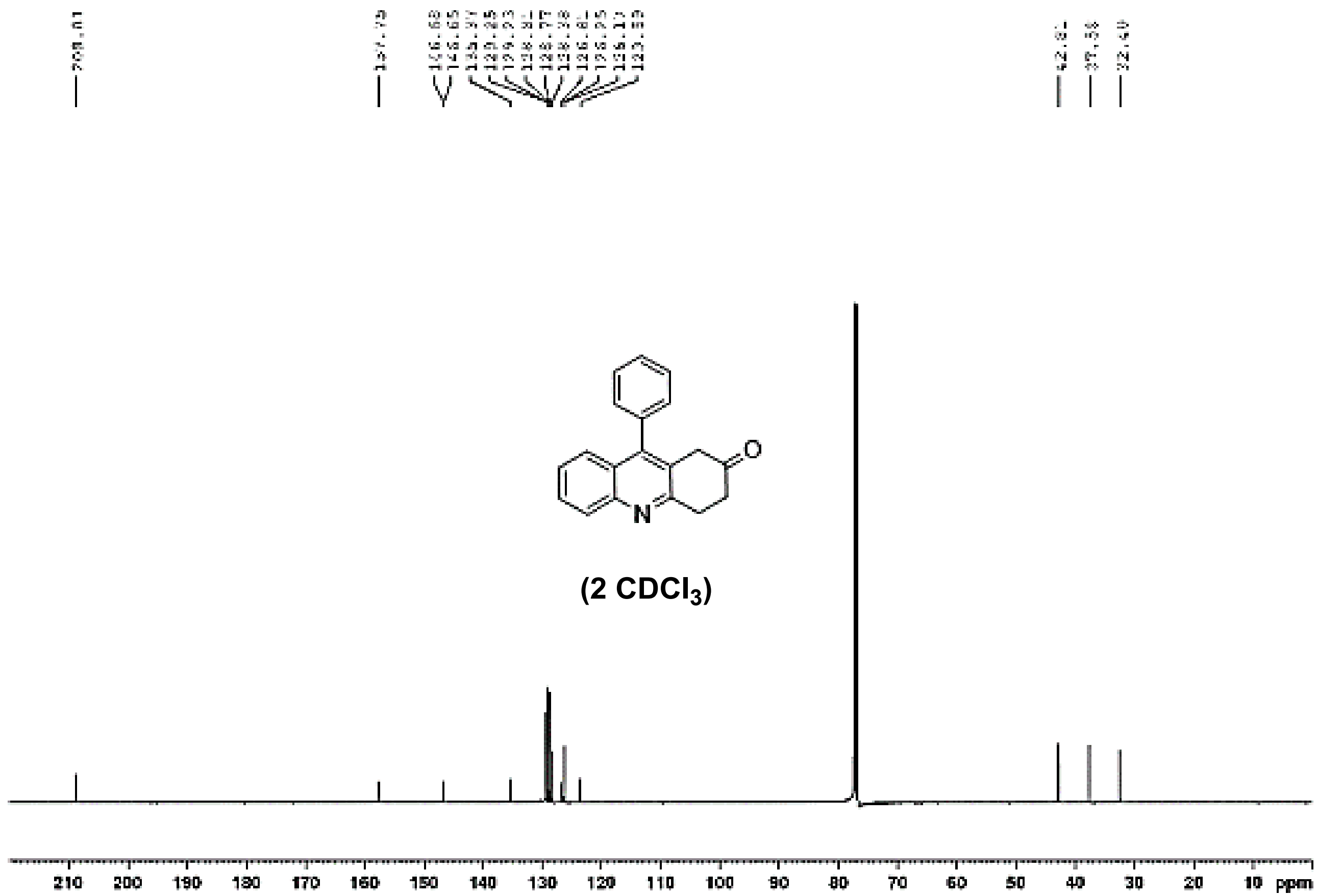

¹³C NMR spectrum (125 MHz) of compound **2**

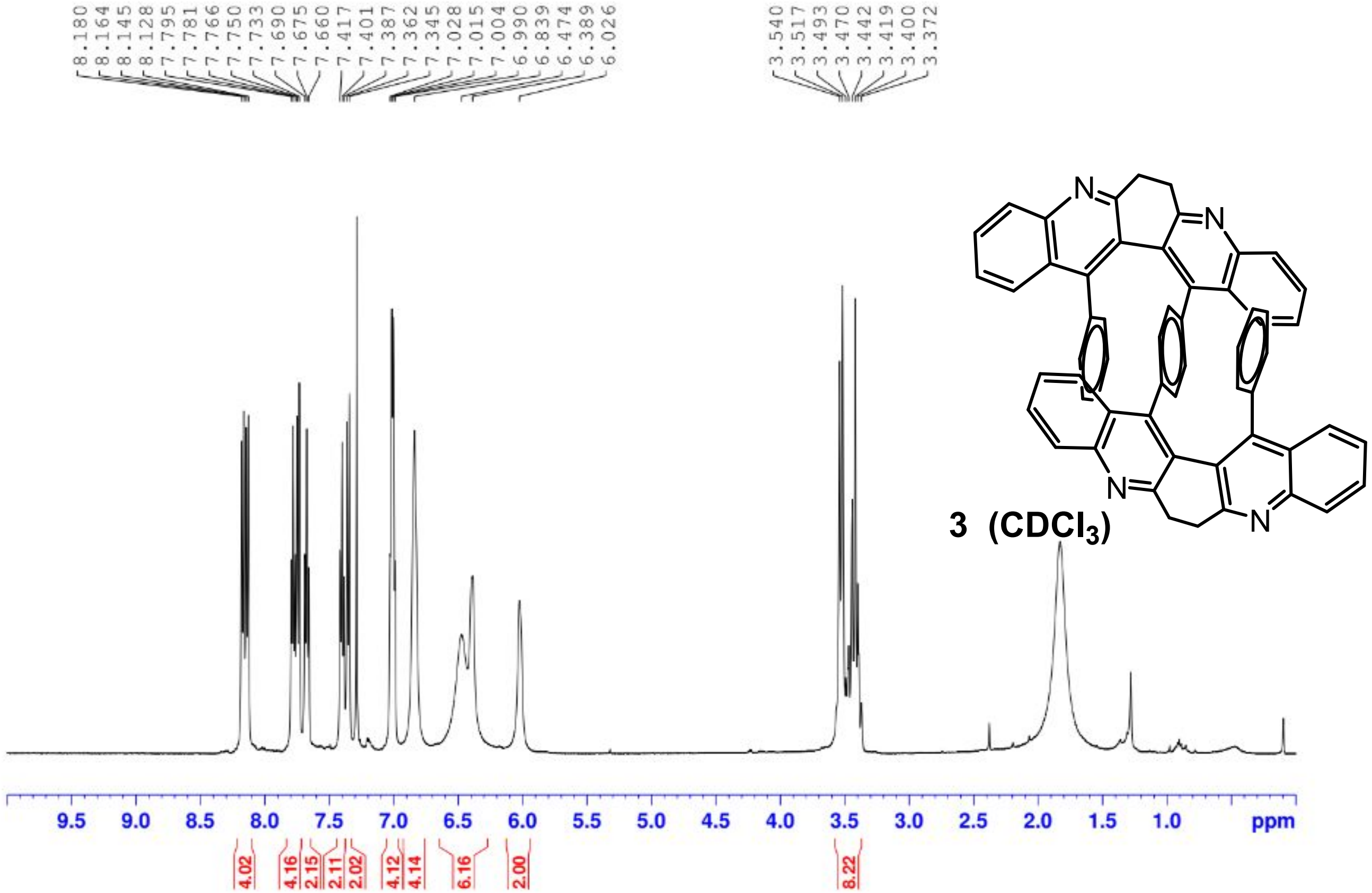

^{1}H NMR spectrum (500 MHz) of compound **3**

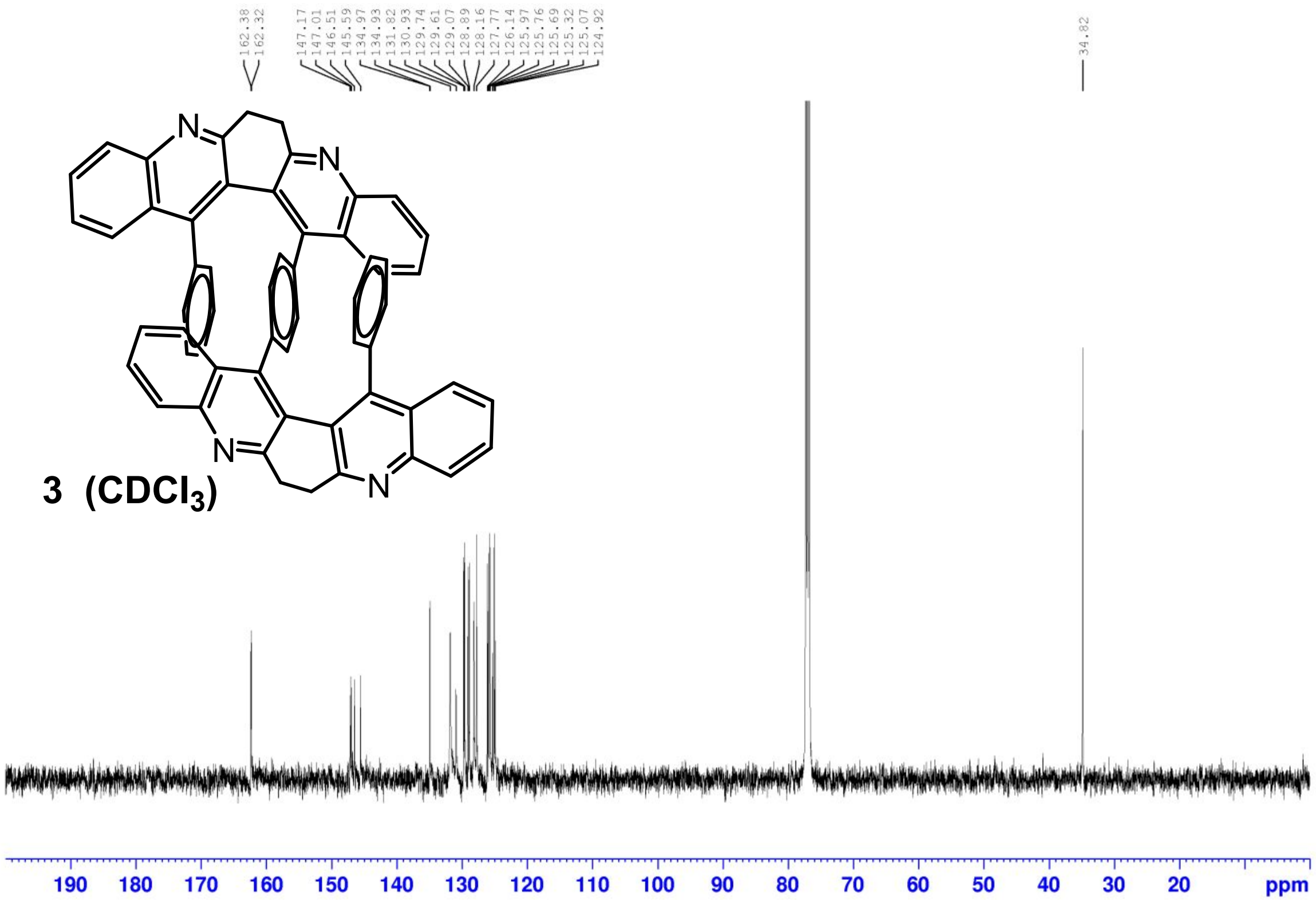

^{13}C NMR spectrum (125 MHz) of compound **3**

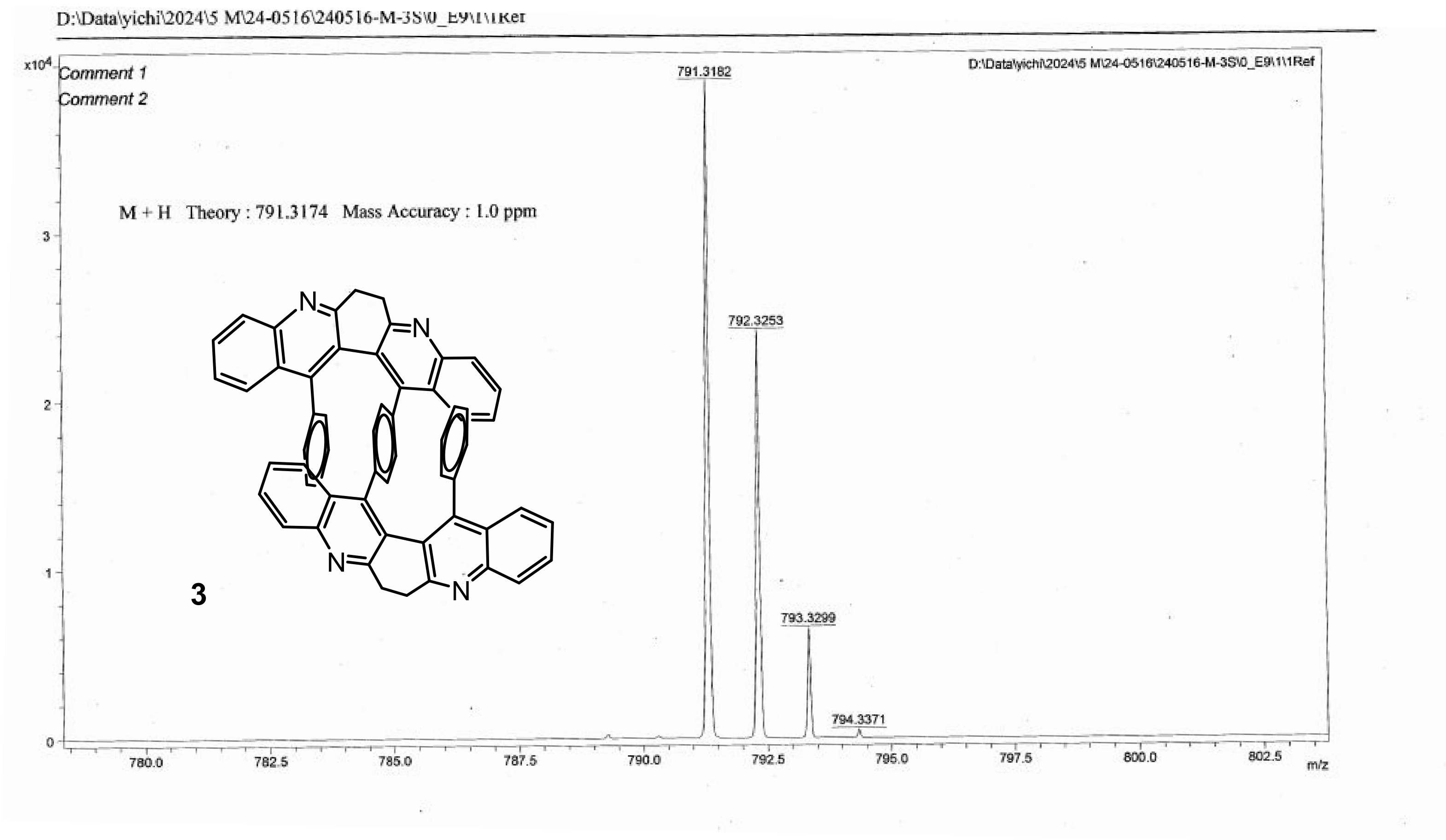

HRMS data of compound **3**

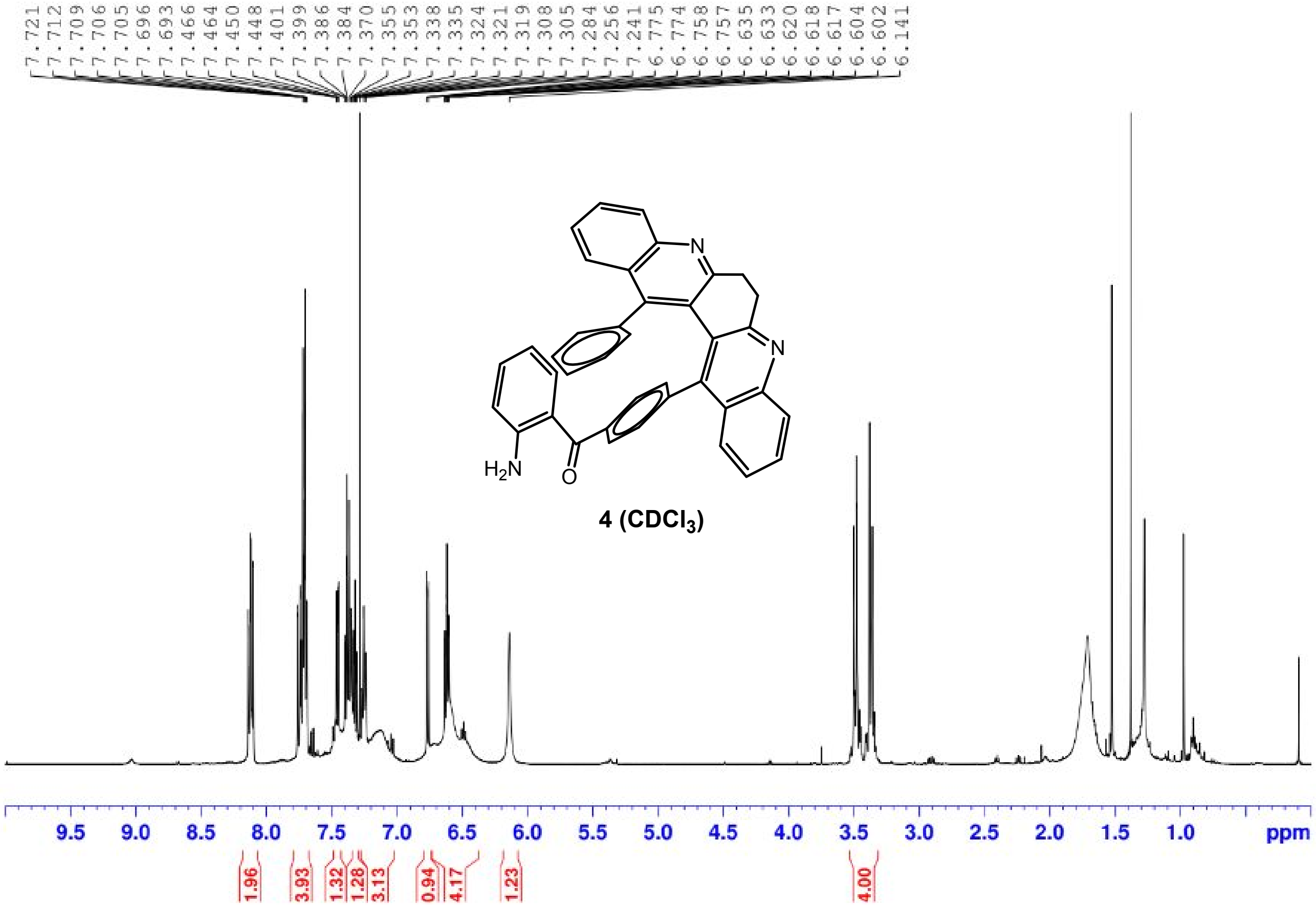

^{1}H NMR spectrum (500 MHz) of compound **4** (contains grease-like impurities)

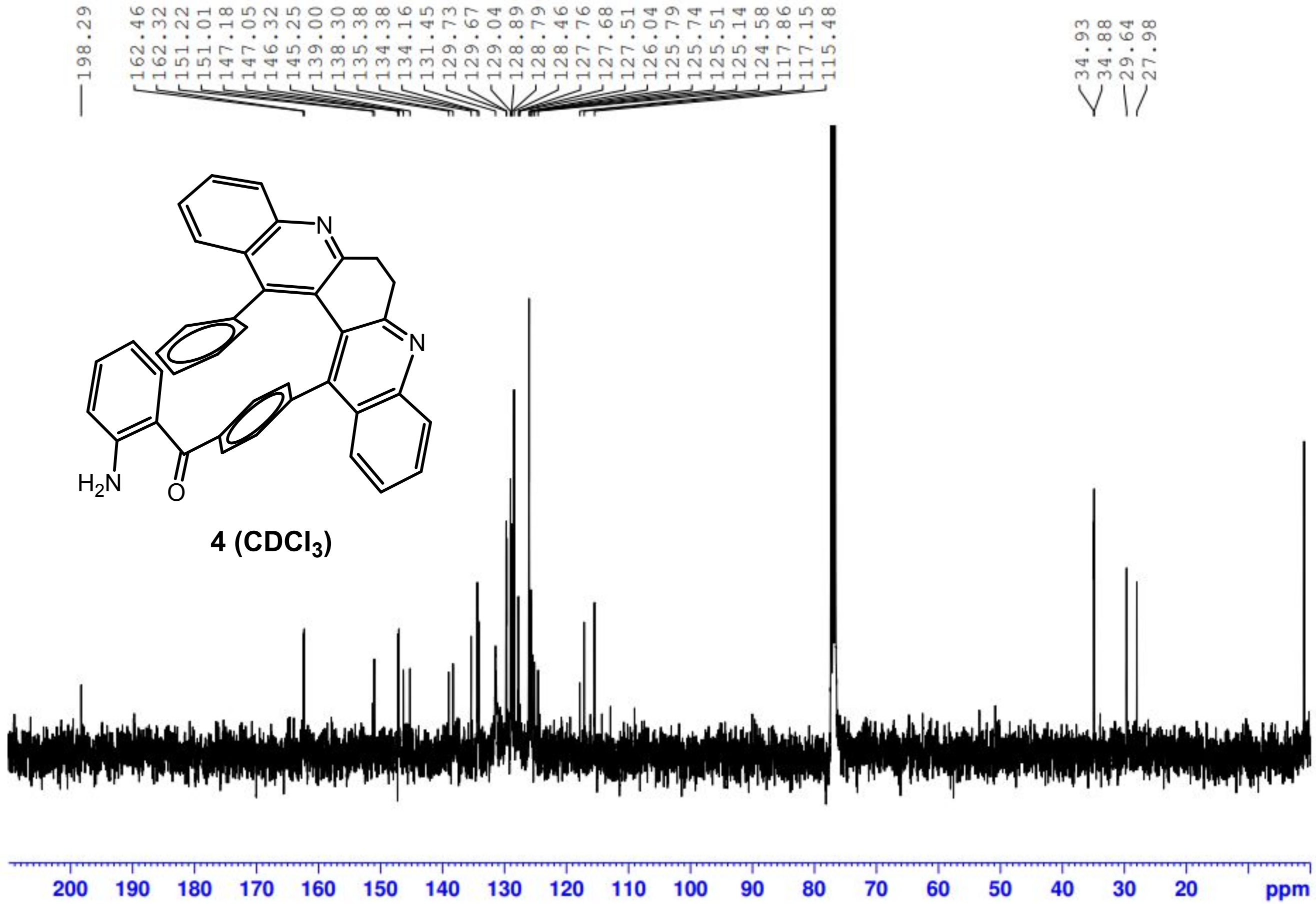

^{13}C NMR spectrum (125 MHz) of compound **4** (contains grease-like impurities)

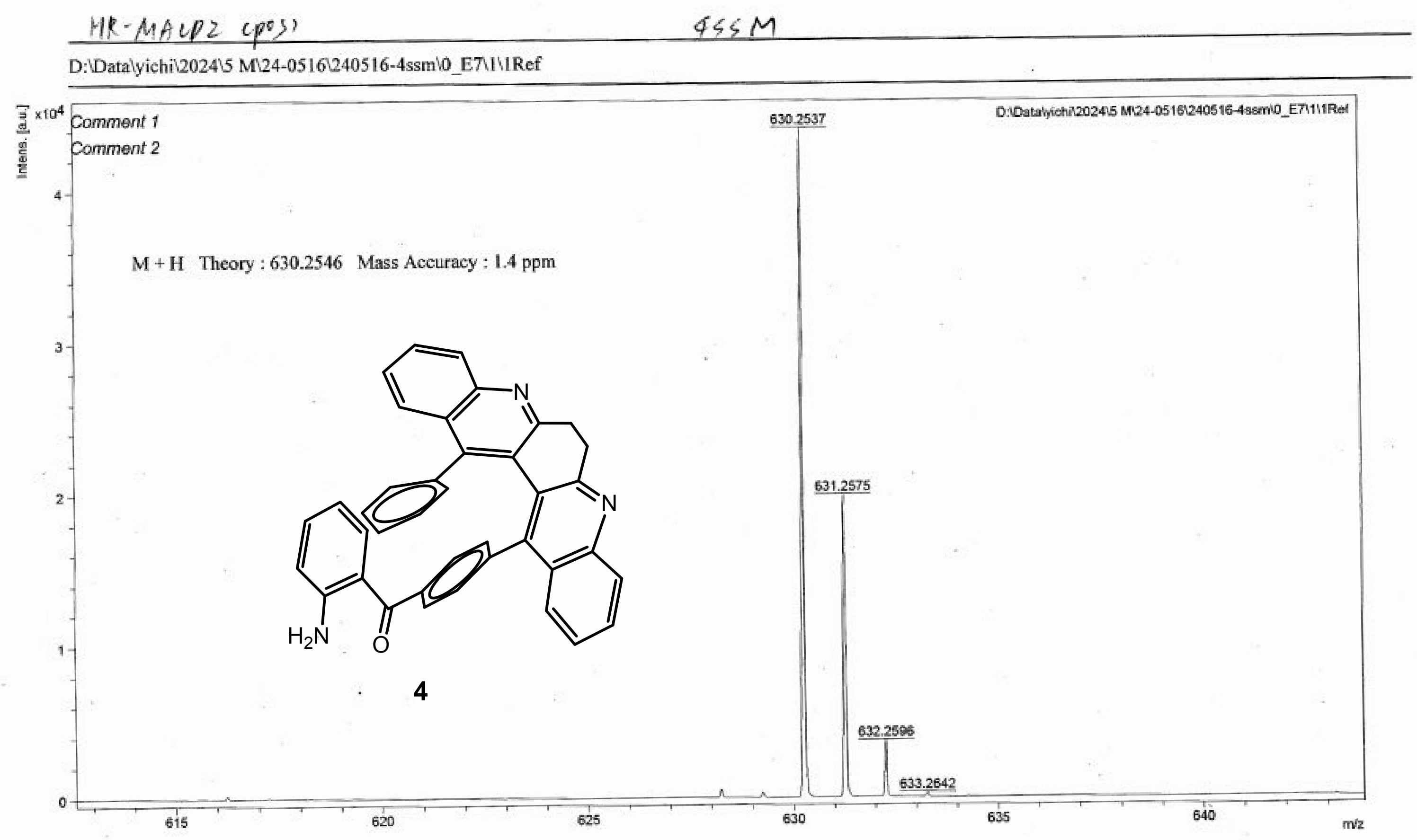

HRMS data of compound **4**

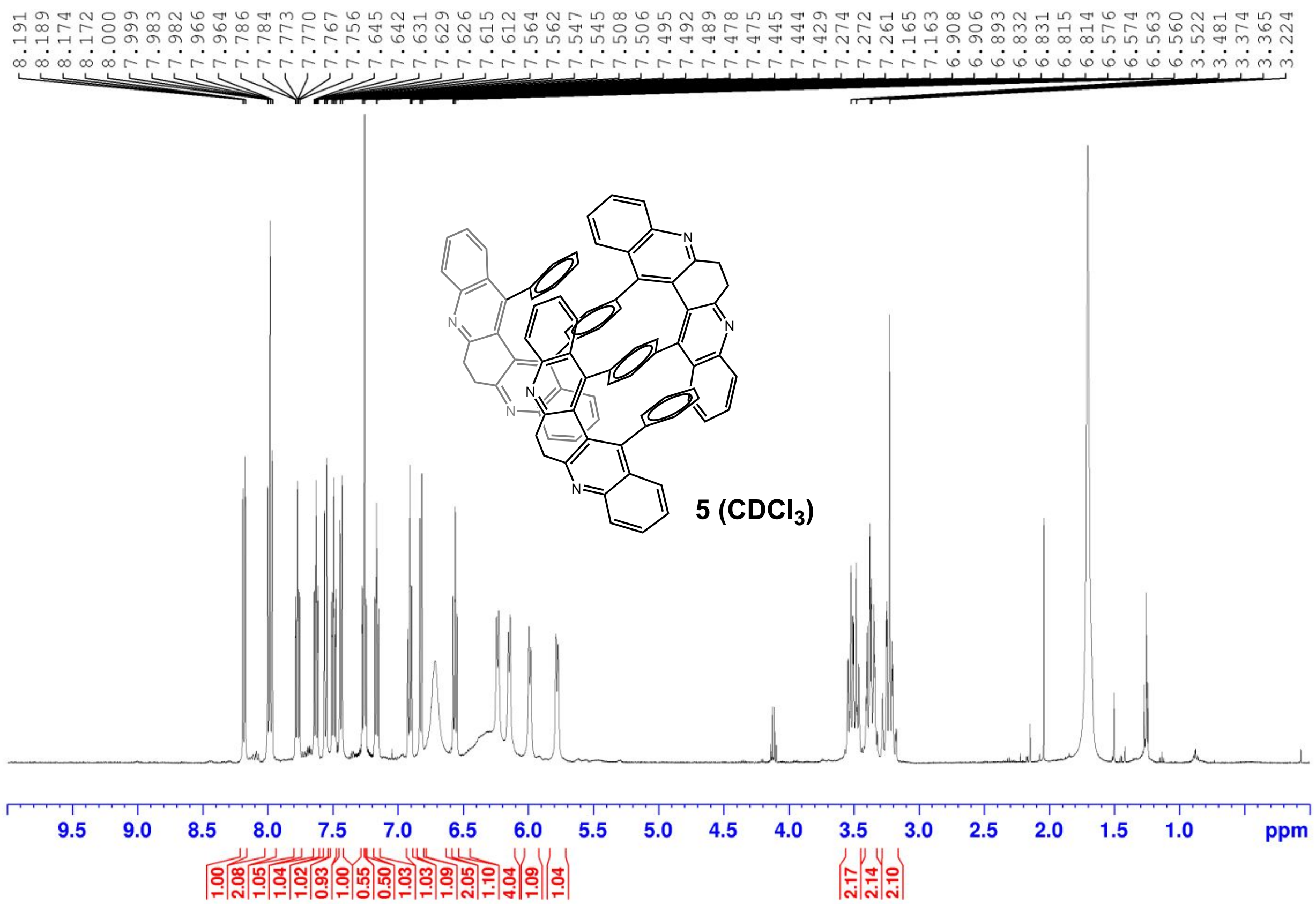

^{1}H NMR spectrum (500 MHz) of compound **5**

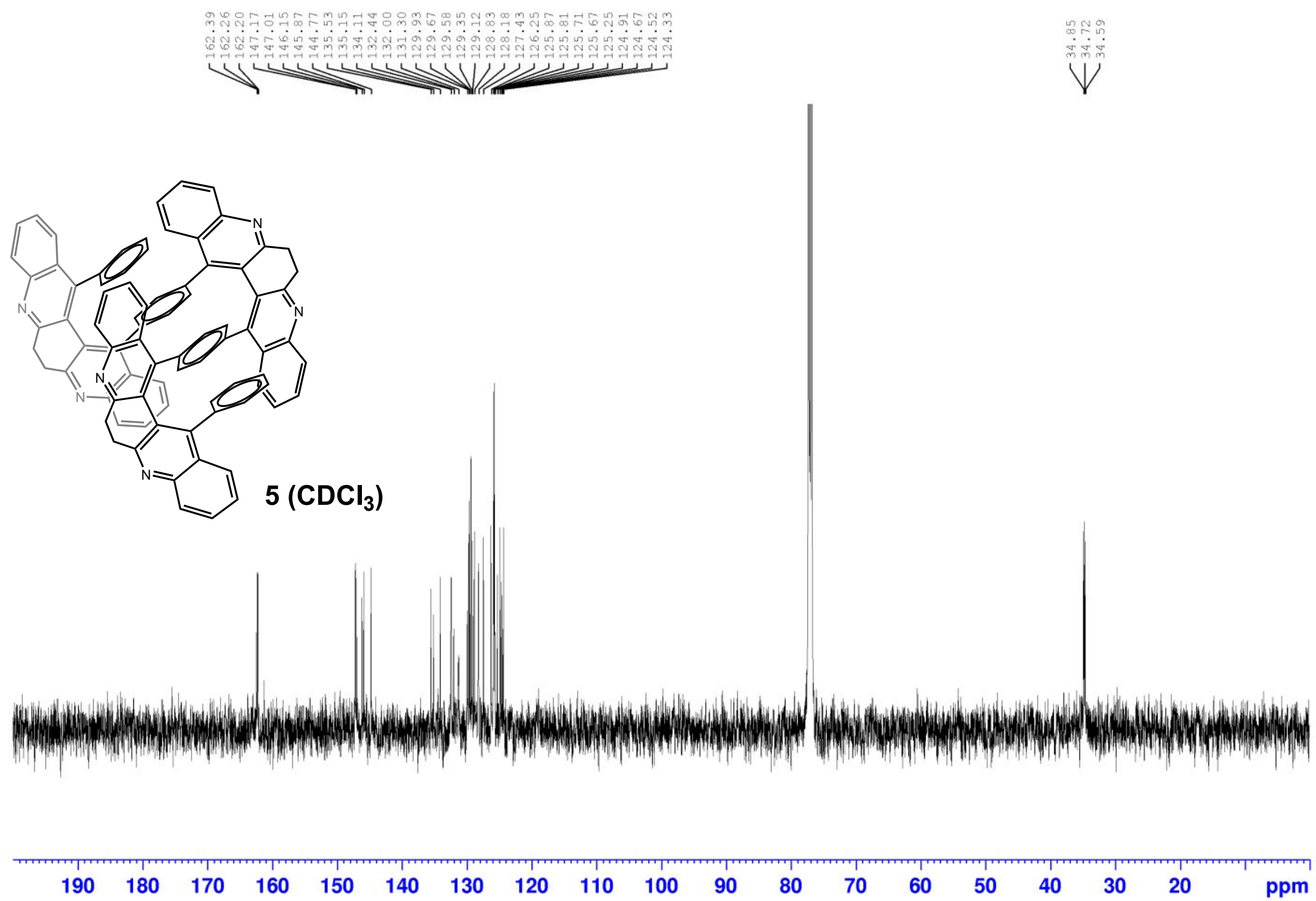

^{13}C NMR spectrum (125 MHz) of compound **5**

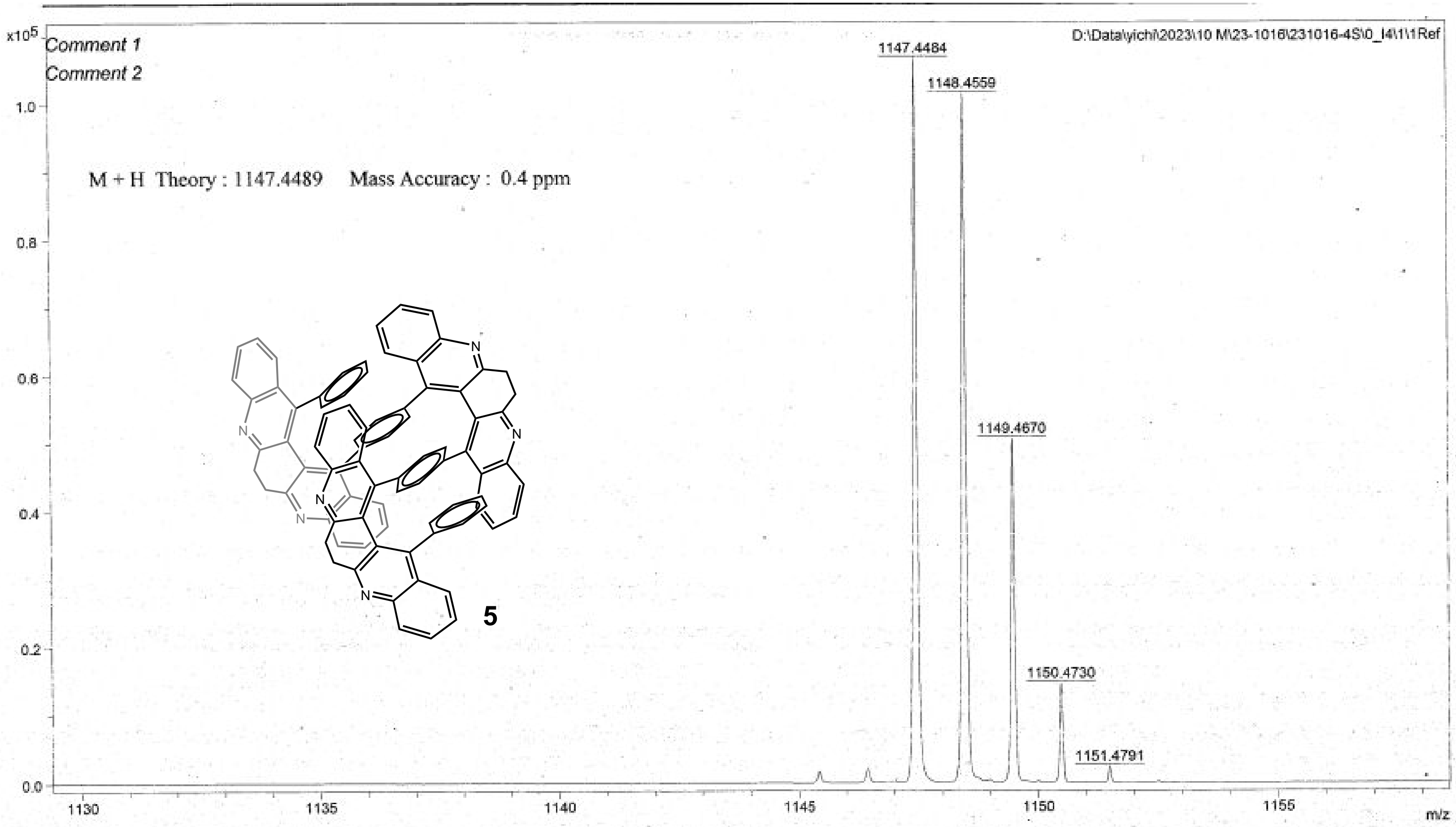

HRMS data of compound **5**

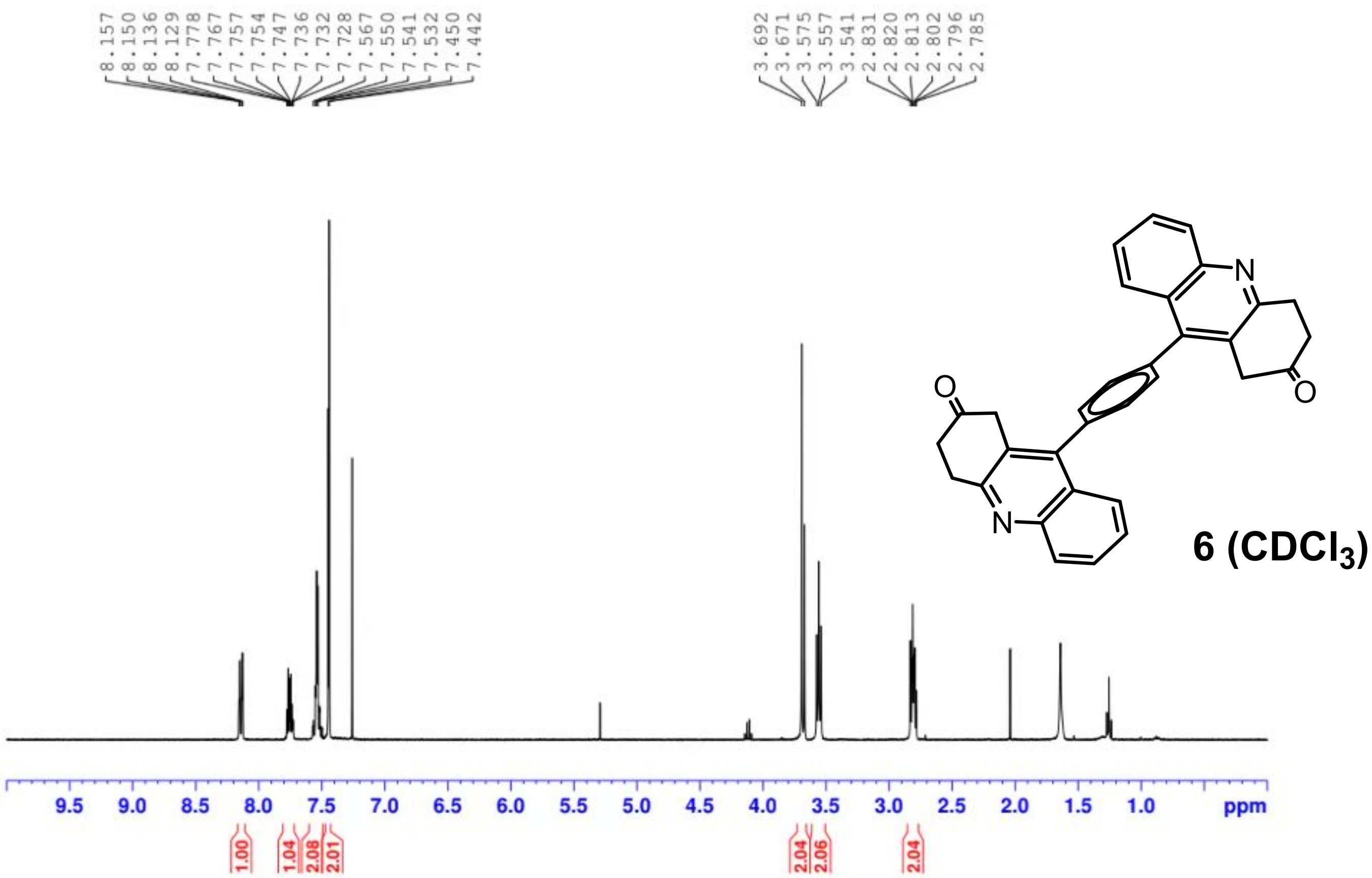

6 ($CDCl_3$)

^{1}H NMR spectrum (500 MHz) of compound **6**

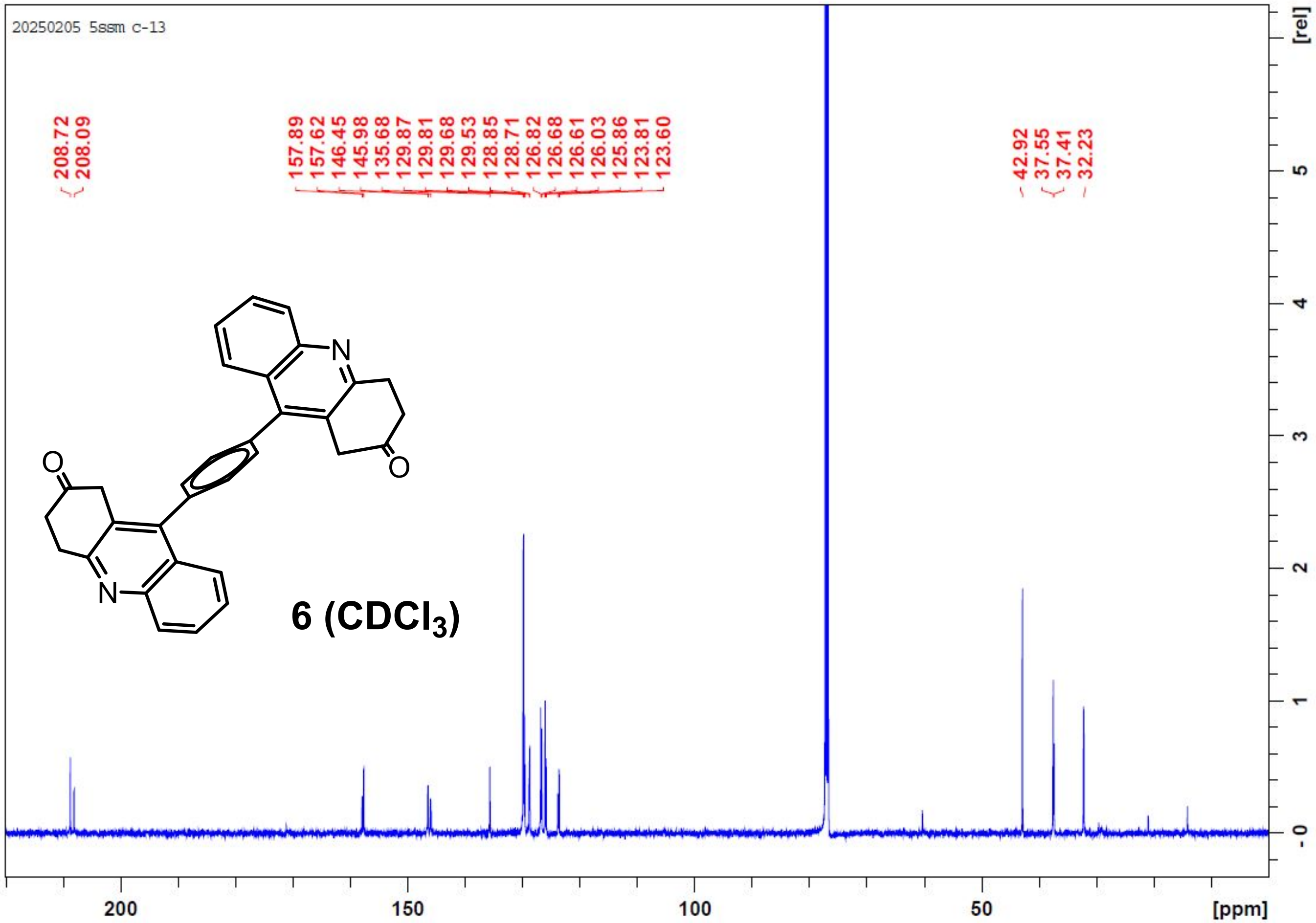

^{13}C NMR spectrum (125 MHz) of compound **6**

5SSM (HR-FAB)

[Mass Spectrum]
Data : 20230816_5SSM-HR-002   Date : 16-Aug-2023 15:29
Sample : 5SSM
Note : NBA
Ion Mode : FAB+
RT : 0.20 min   Scan# : 4
Elements : C 1000/0, H 1000/0, N 2/2, O 2/2
Mass Tolerance   : 50mmu
Unsaturation (U.S.) : -0.5 - 1000.0

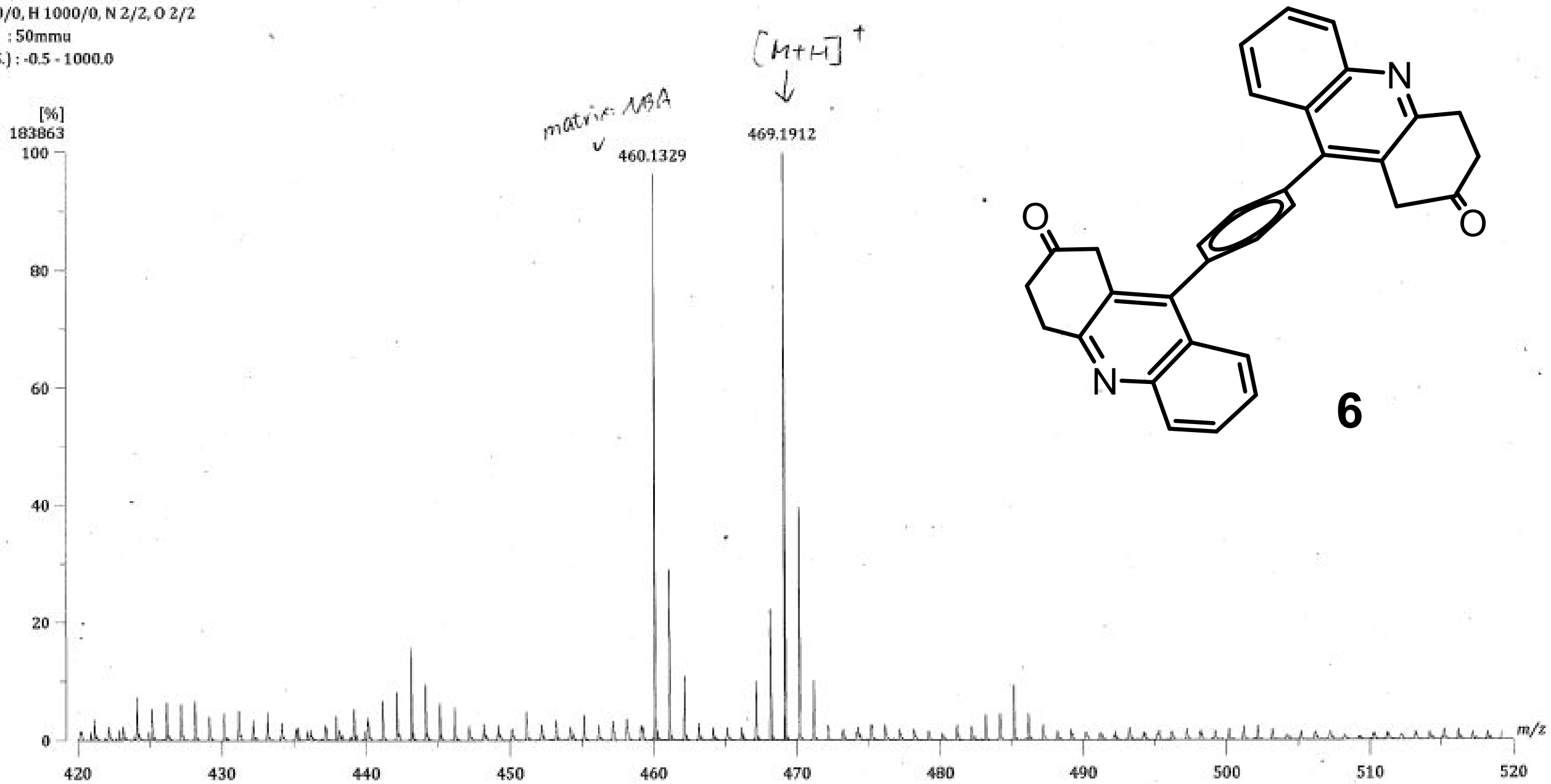

6

Observed m/z	Int%					
469.1912	100.00					

	Estimated m/z	Err[ppm / mmu]	U.S.	C	H	N	O
1	469.1916	-0.9 / -0.4	21.5	32	25	2	2

HRMS data of compound **6**

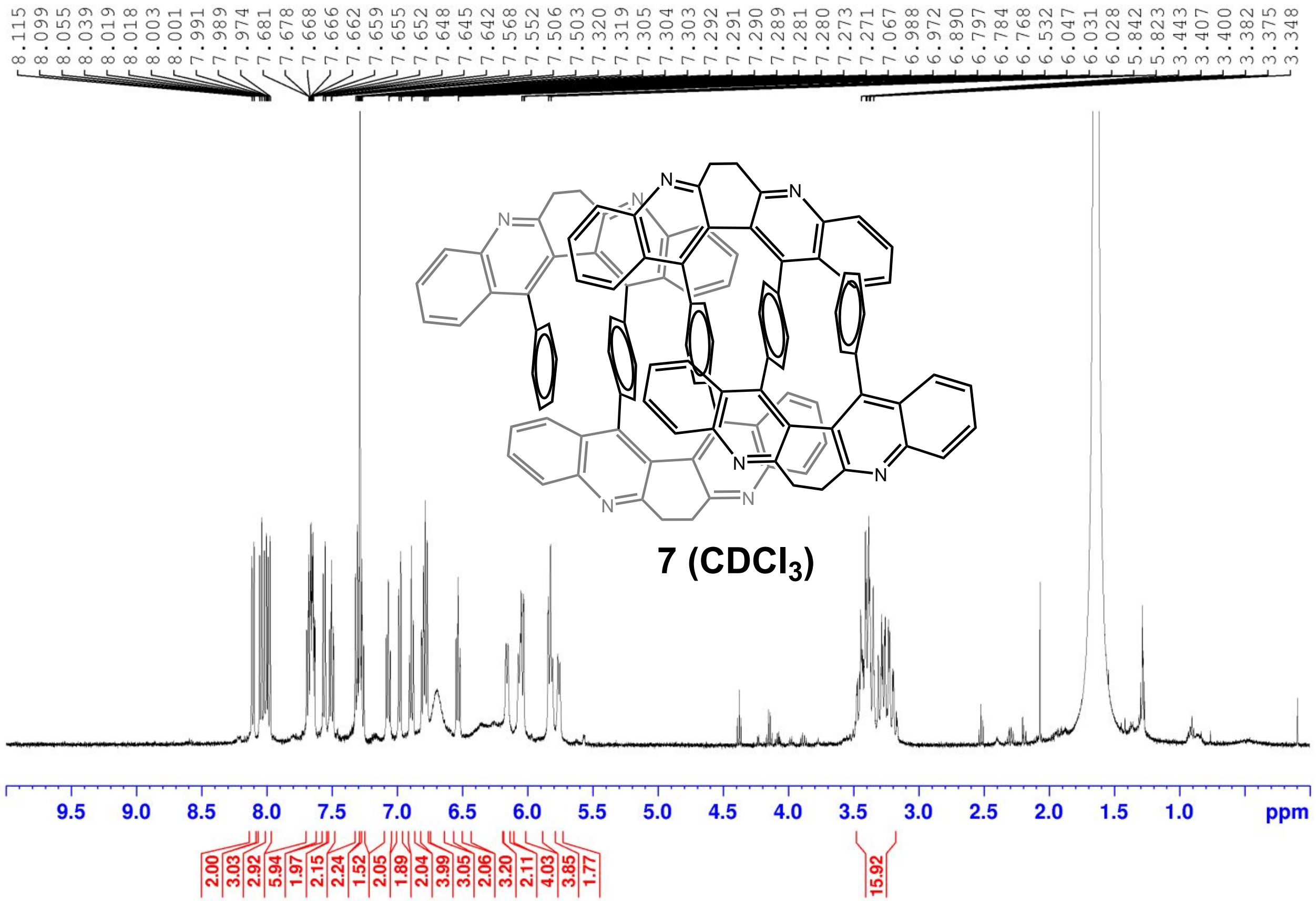

^{1}H NMR spectrum (500 MHz) of compound **7**

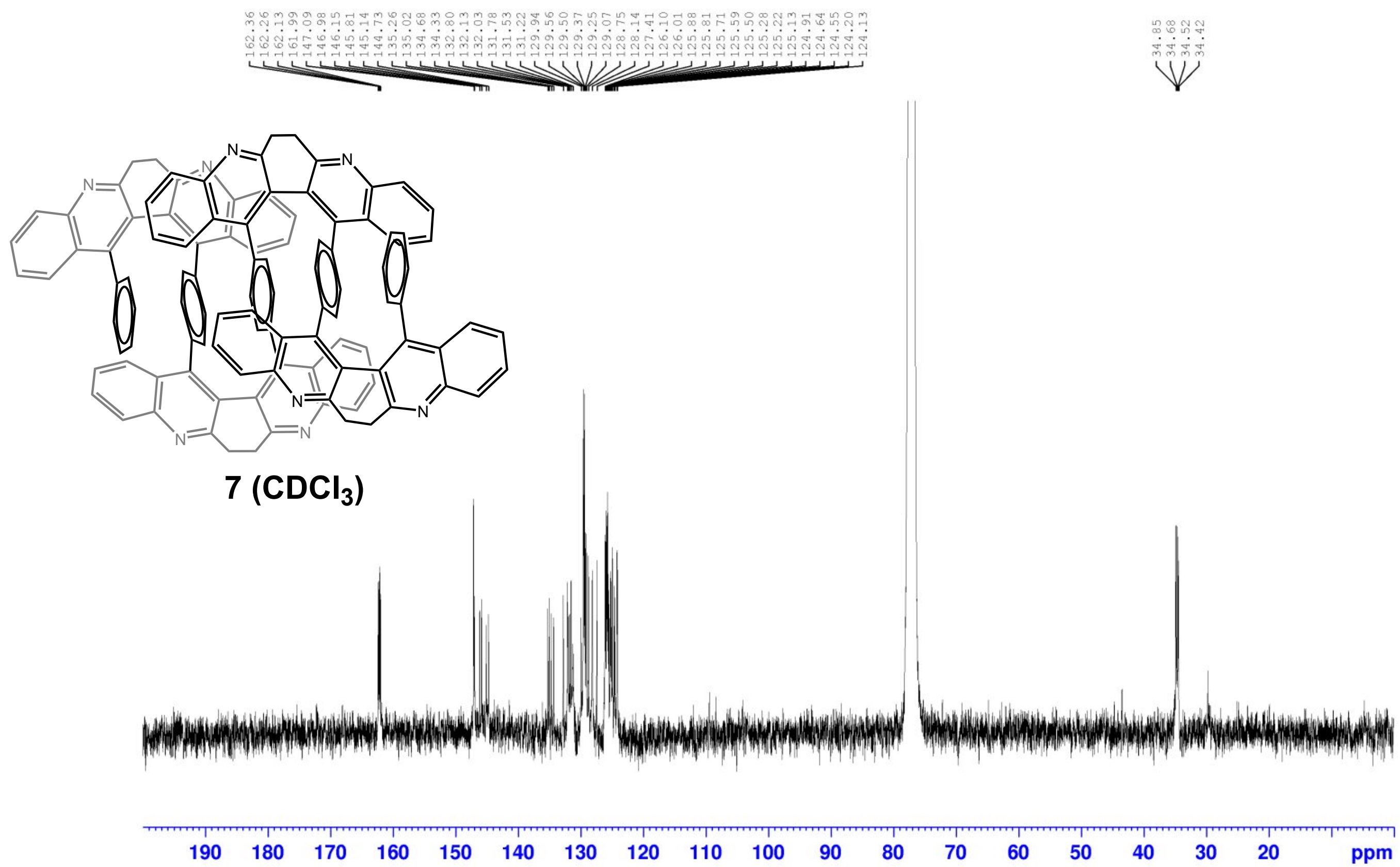

^{13}C NMR spectrum (125 MHz) of compound **7**

[Mass Spectrum]
Data : 20230816_5s unsolu swear-HR-002   Date : 16-Aug-2023 15:41
Sample : 5s unsolu swear
Note : NBA
Ion Mode : FAB+
RT : 0.00 min   Scan# : (1,10)
Elements : C 1000/0, H 1000/0, N 8/8
Mass Tolerance   : 5mmu
Unsaturation (U.S.) : -0.5 - 1000.0

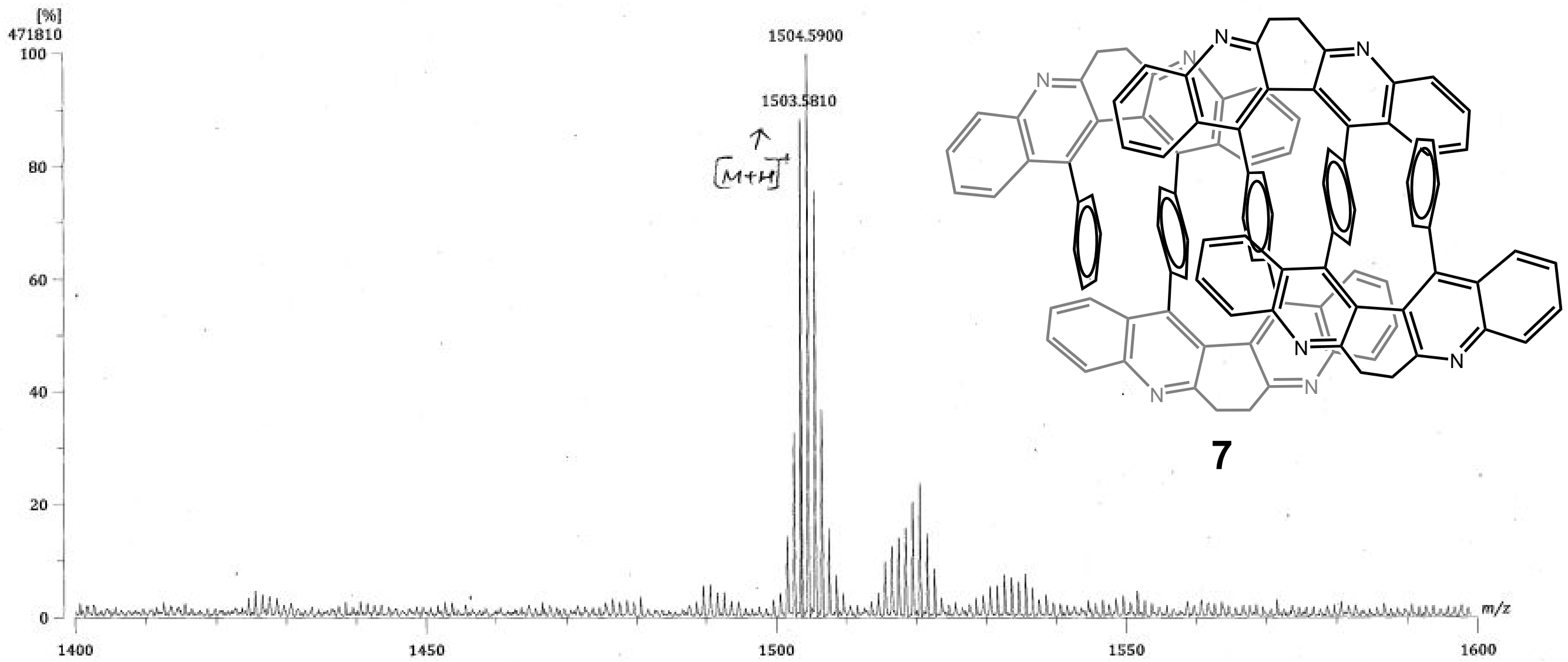

Observed m/z	Int%					
1503.5810	88.53					

	Estimated m/z	Err[ppm / mmu]	U.S.	C	H	N
1	1503.5802	+0.6 / +0.8	79.5	110	71	8

HRMS data of compound **7**

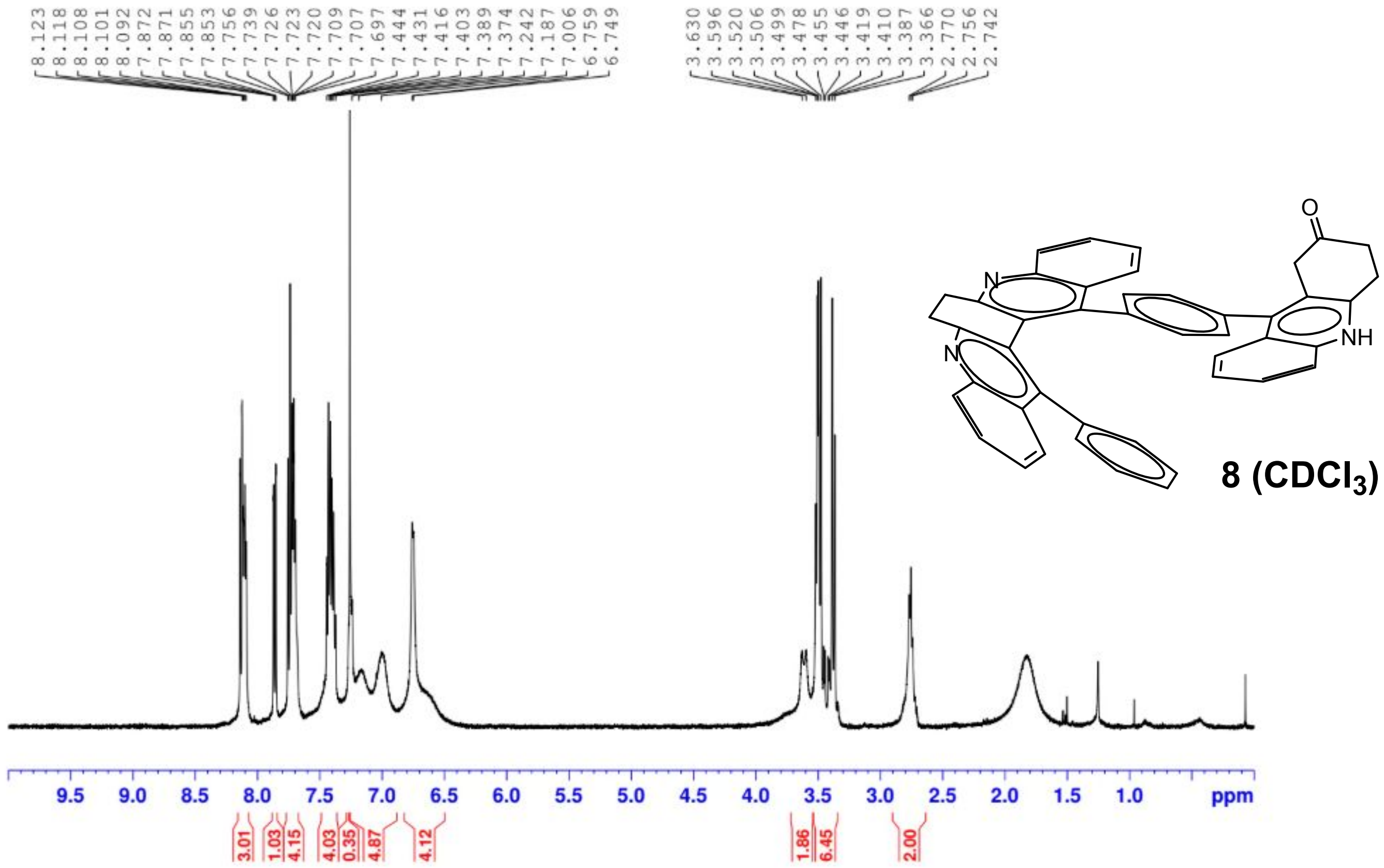

8 ($CDCl_3$)

^{1}H NMR spectrum (500 MHz) of compound **8**

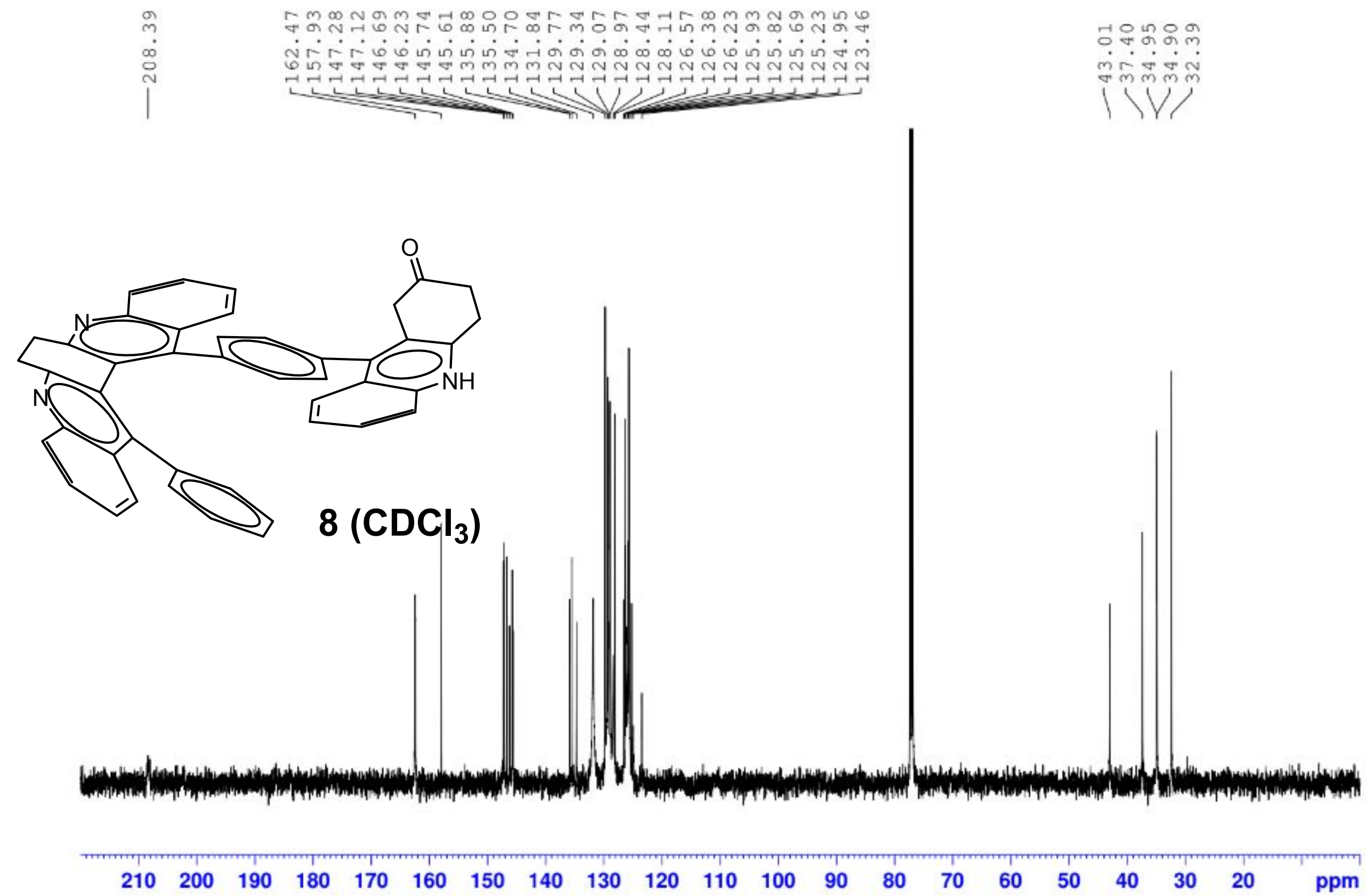

^{13}C NMR spectrum (125 MHz) of compound **8**

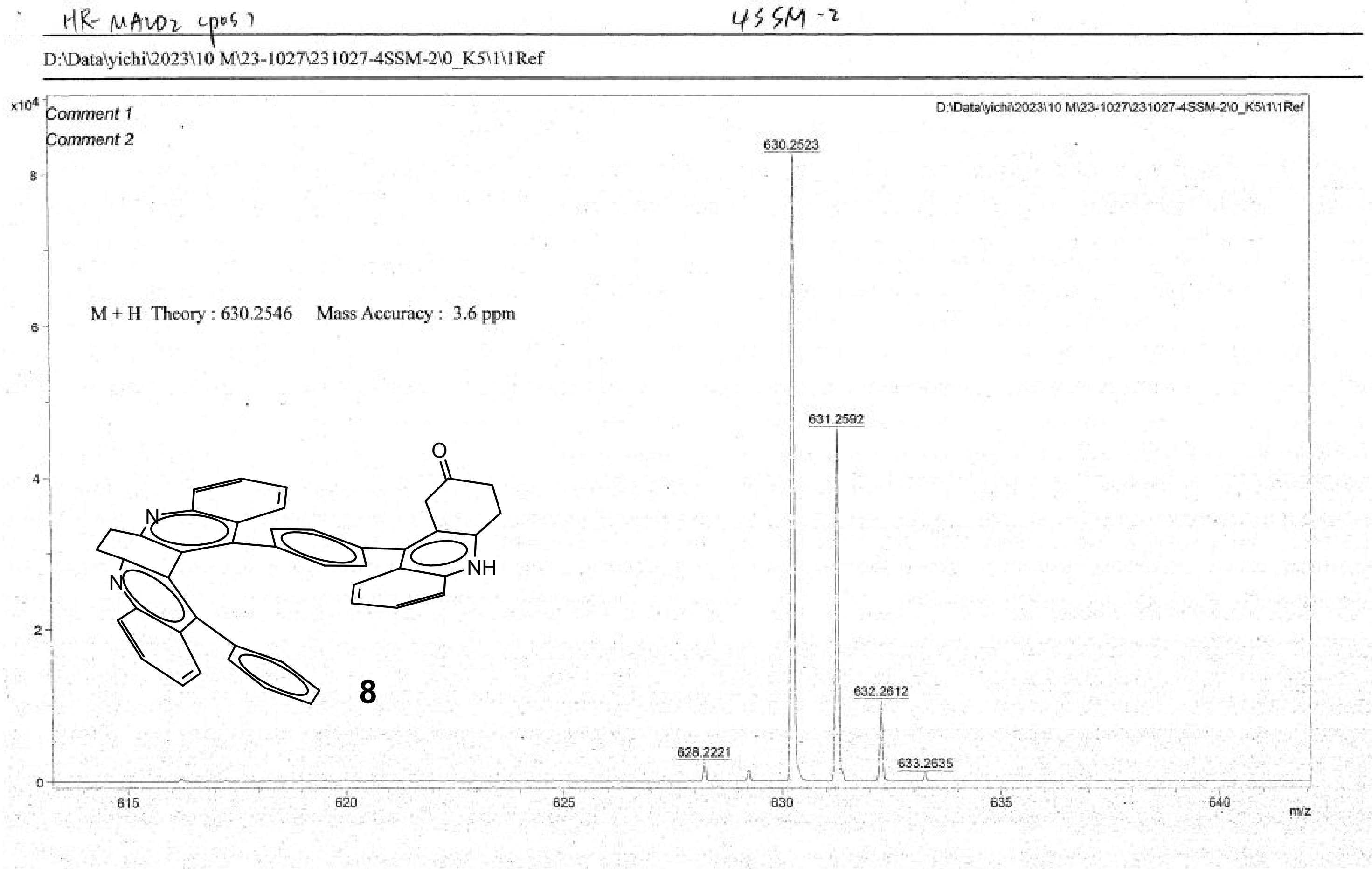

HRMS data of compound **8**

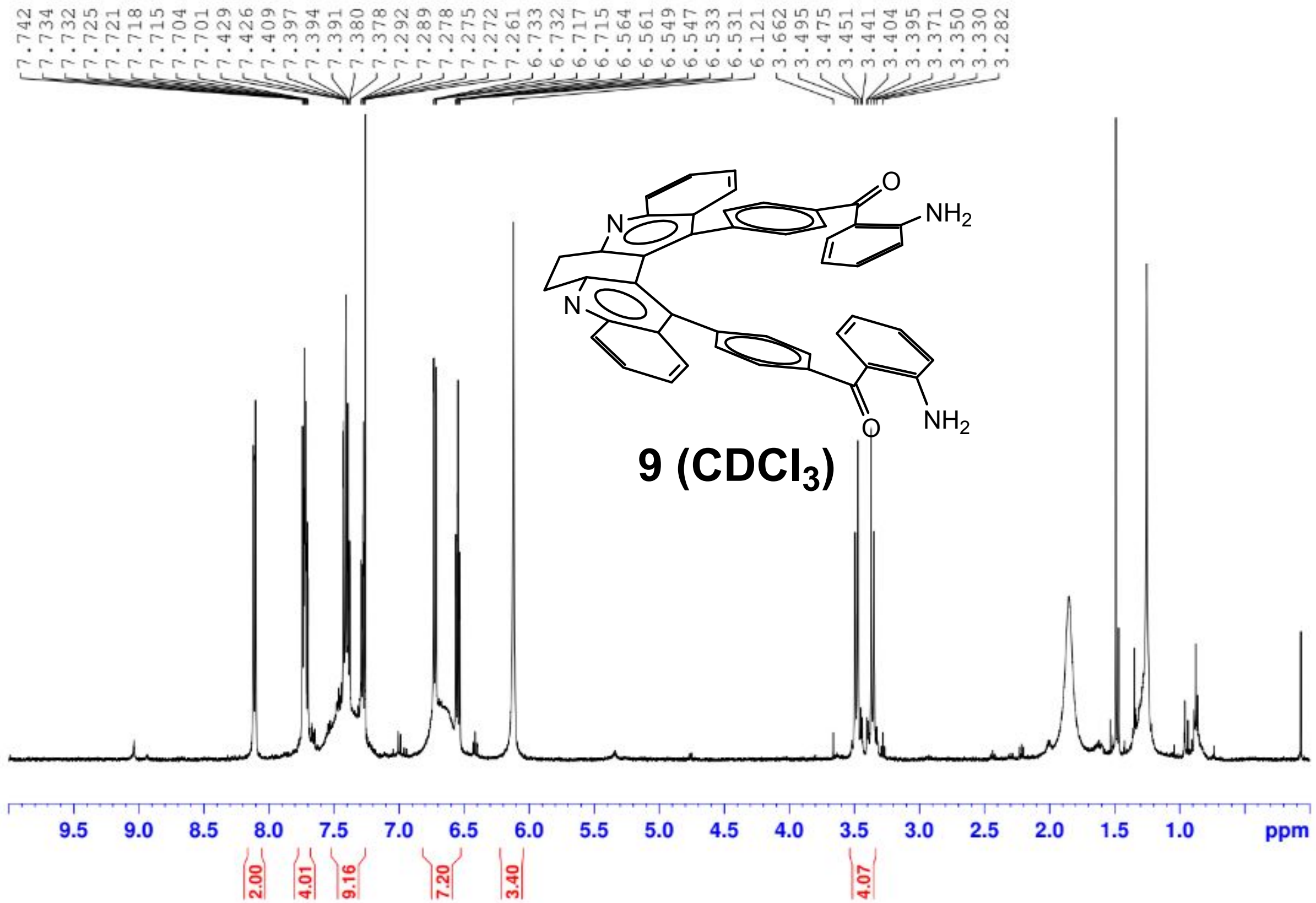

^{1}H NMR spectrum (500 MHz) of compound **9**

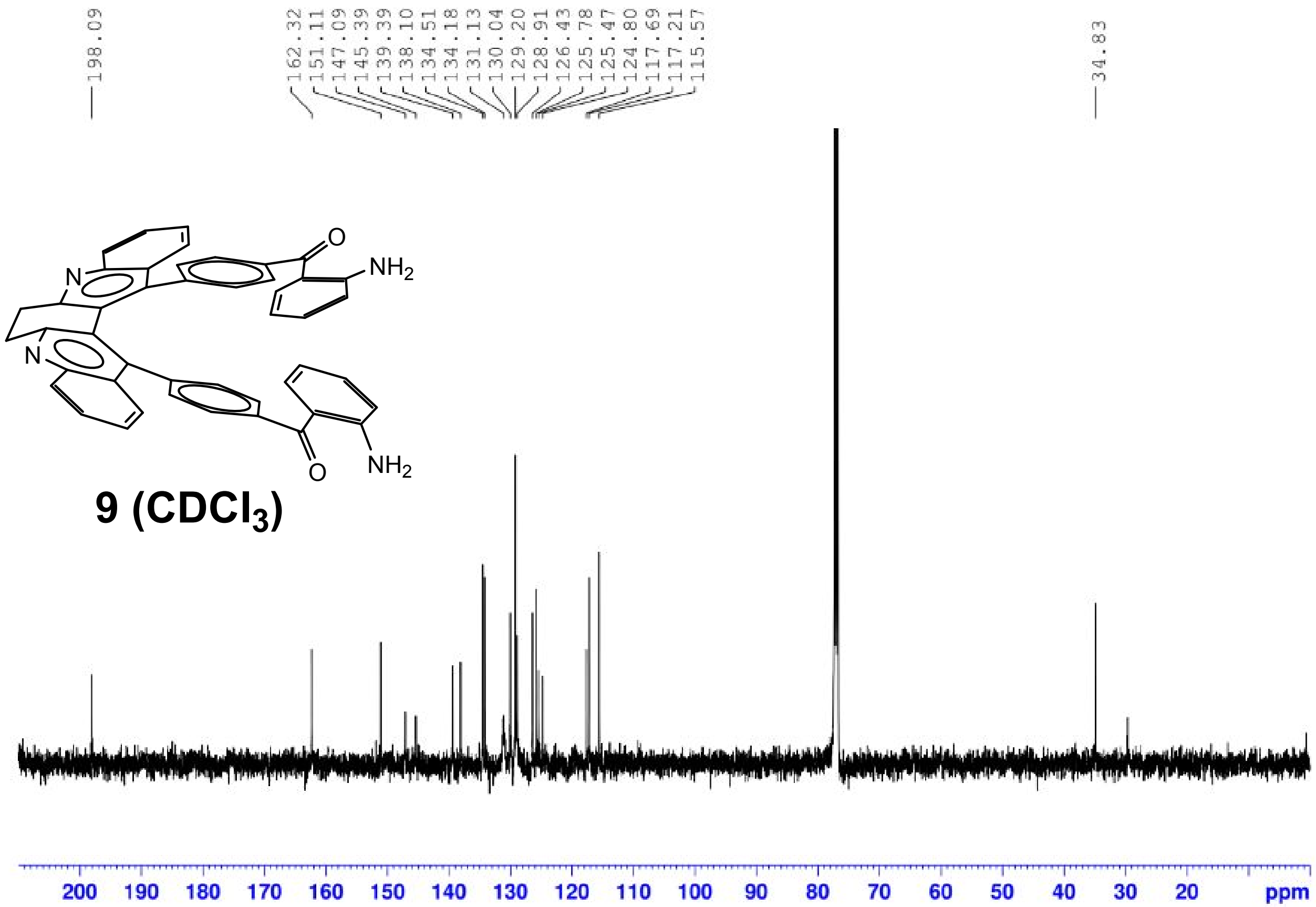

^{13}C NMR spectrum (125 MHz) of compound **9**

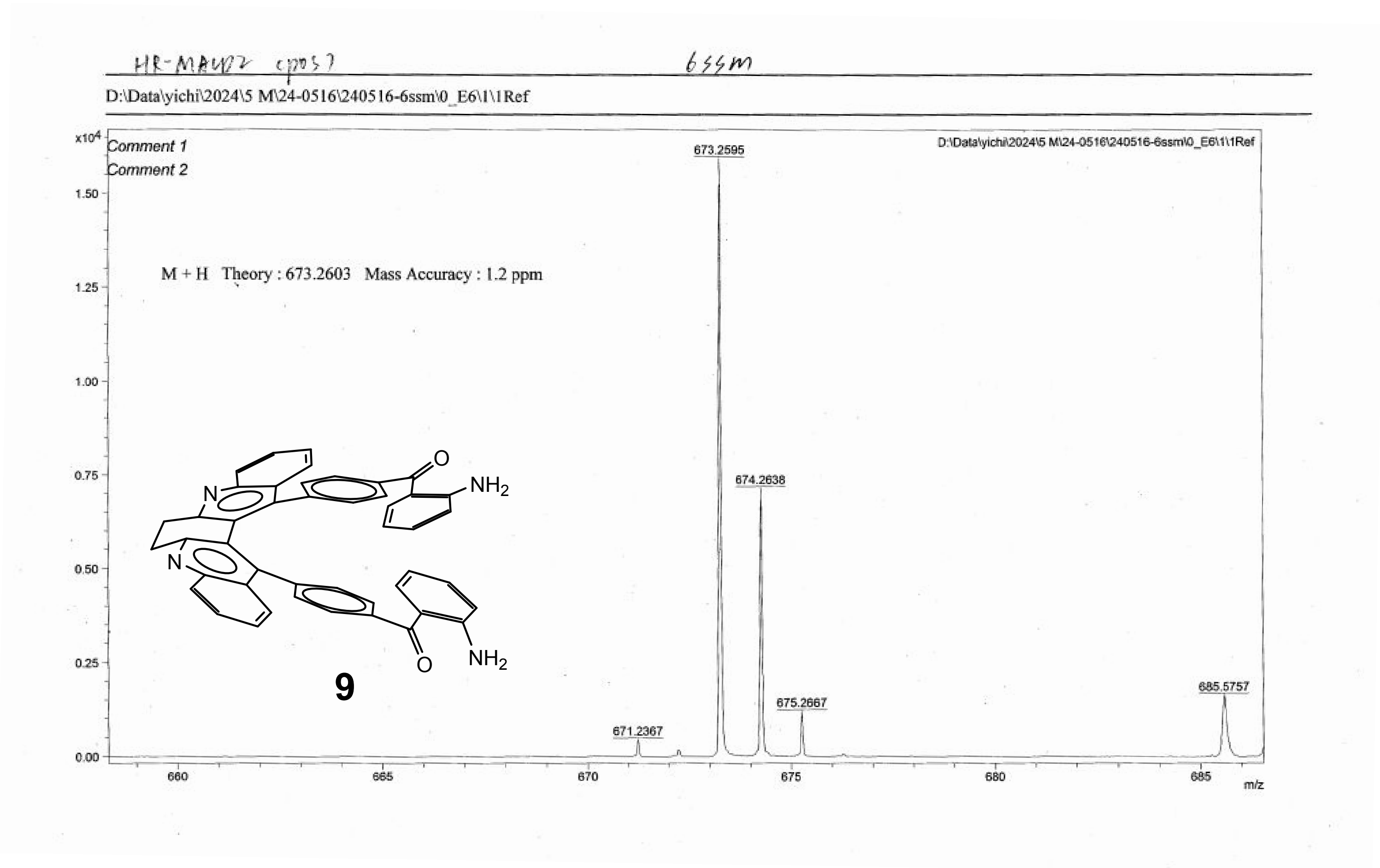

HRMS data of compound **9**

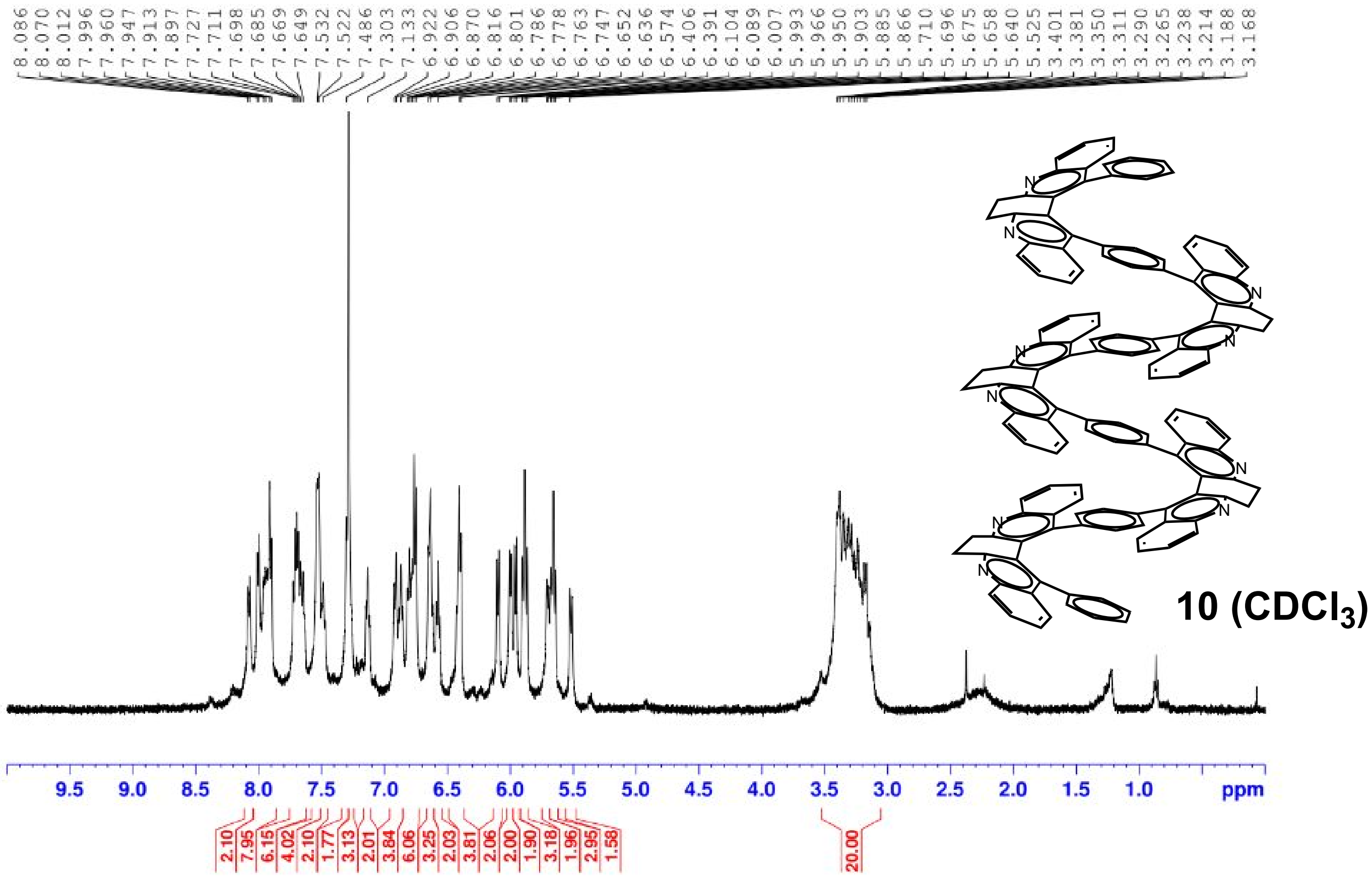

^{1}H NMR spectrum (500 MHz) of compound **10**

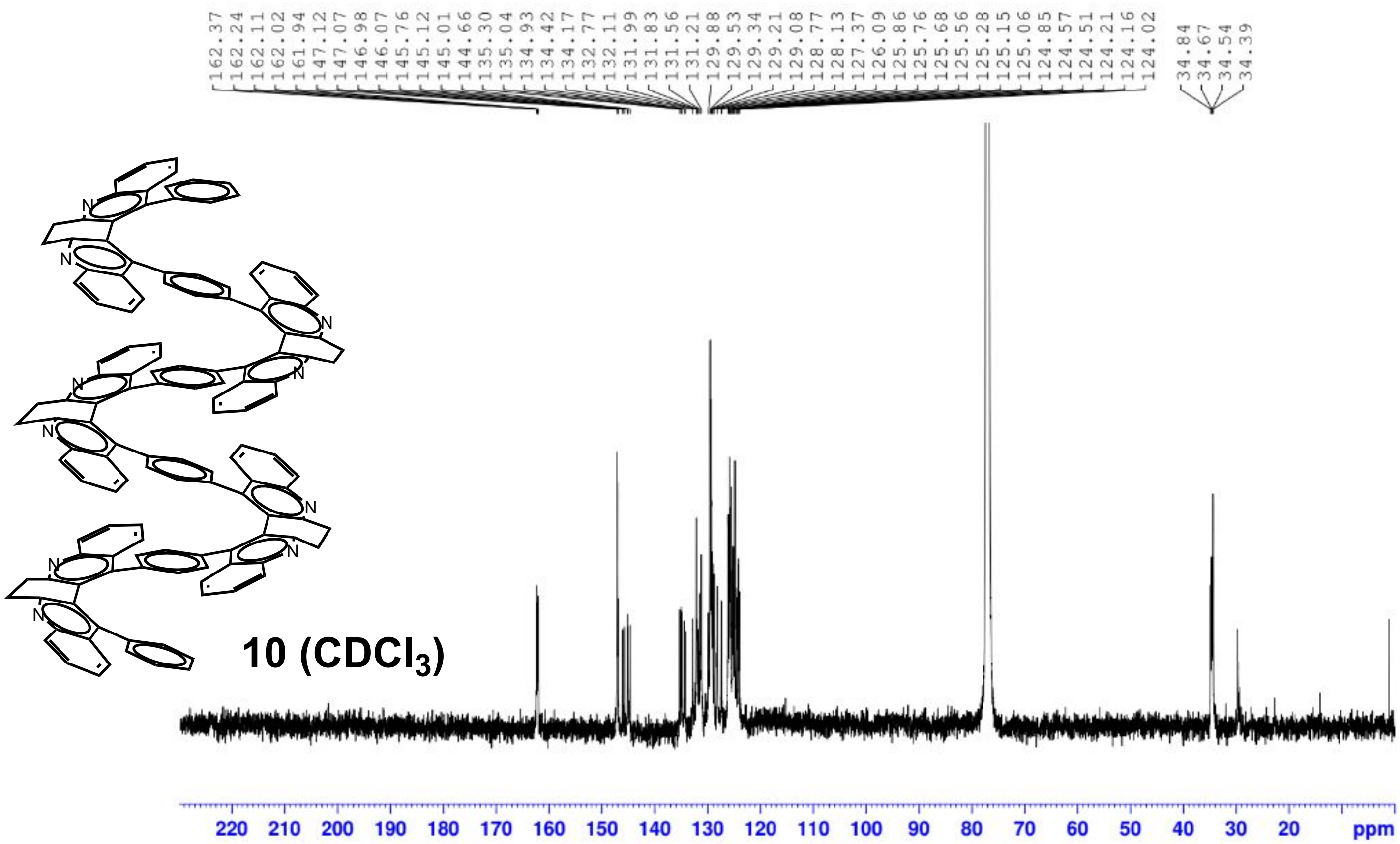

^{13}C NMR spectrum (125 MHz) of compound **10**

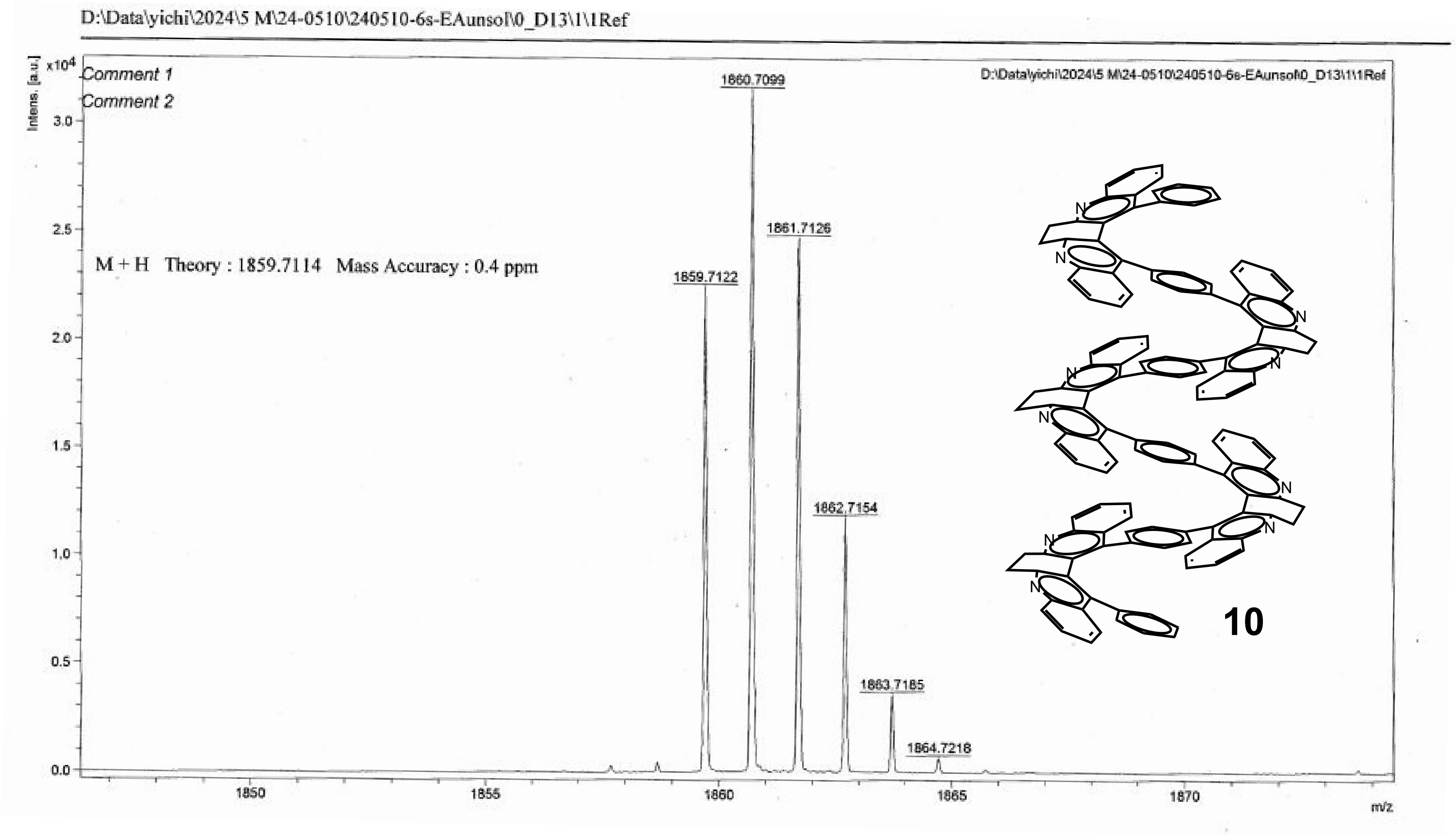

HRMS data of compound **10**

	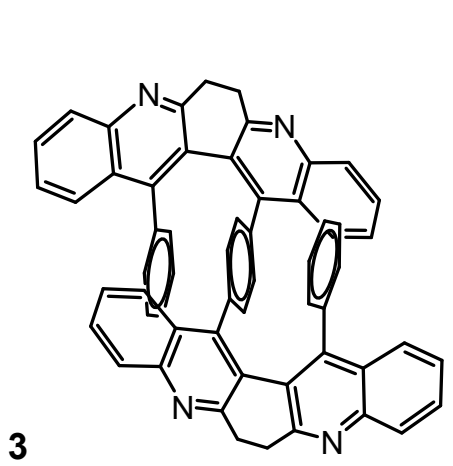 3	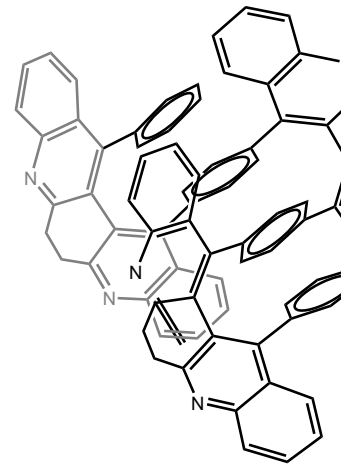 5	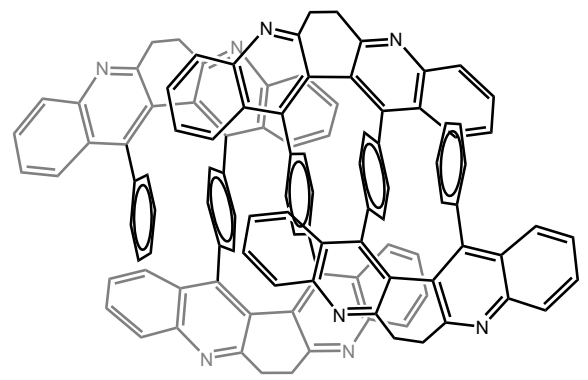 7	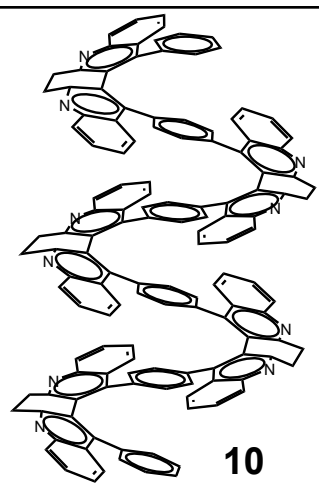 10
**Expected numbers of ^{1}H NMR signals from static phenylene units**	**2**	**4**	**6**	**8**
**Observed**	6.4, 6.0 ppm	5.8, 6.0, 6.2, 6.3 ppm	5.75, 5.78–5.85 (×2)[a], 6.04 (×2)[a], 6.15 ppm	5.51, 5.64–5.68 (×2)[a], 5.70, 5.87–5.90 (×2)[a], 5.96, 6.00 ppm
**Expected numbers of ^{13}C NMR signals from quinoline units**	**2**	**3**	**4**	**5**
**Observed**	162.38, 162.32 ppm	162.39, 162.26, 162.20 ppm	162.36, 162.25, 162.12, 161.99 ppm	162.37, 162.24, 162.11, 162.02, 161.94 ppm
**Expected numbers of ^{13}C NMR signals from ethylene bridges**	**2**	**3**	**4**	**5**
**Observed**	34.8 ppm (×2)[a]	34.82, 34.69, 34.56 ppm	34.85, 34.68, 34.52, 34.42 ppm	34.84, 34.67, 34.54, 34.39 (×2)[a] ppm

Table S1. Fine spectral features of multilayer phenylene compounds **3, 5, 7**, and **10**

a. two overlapping signals

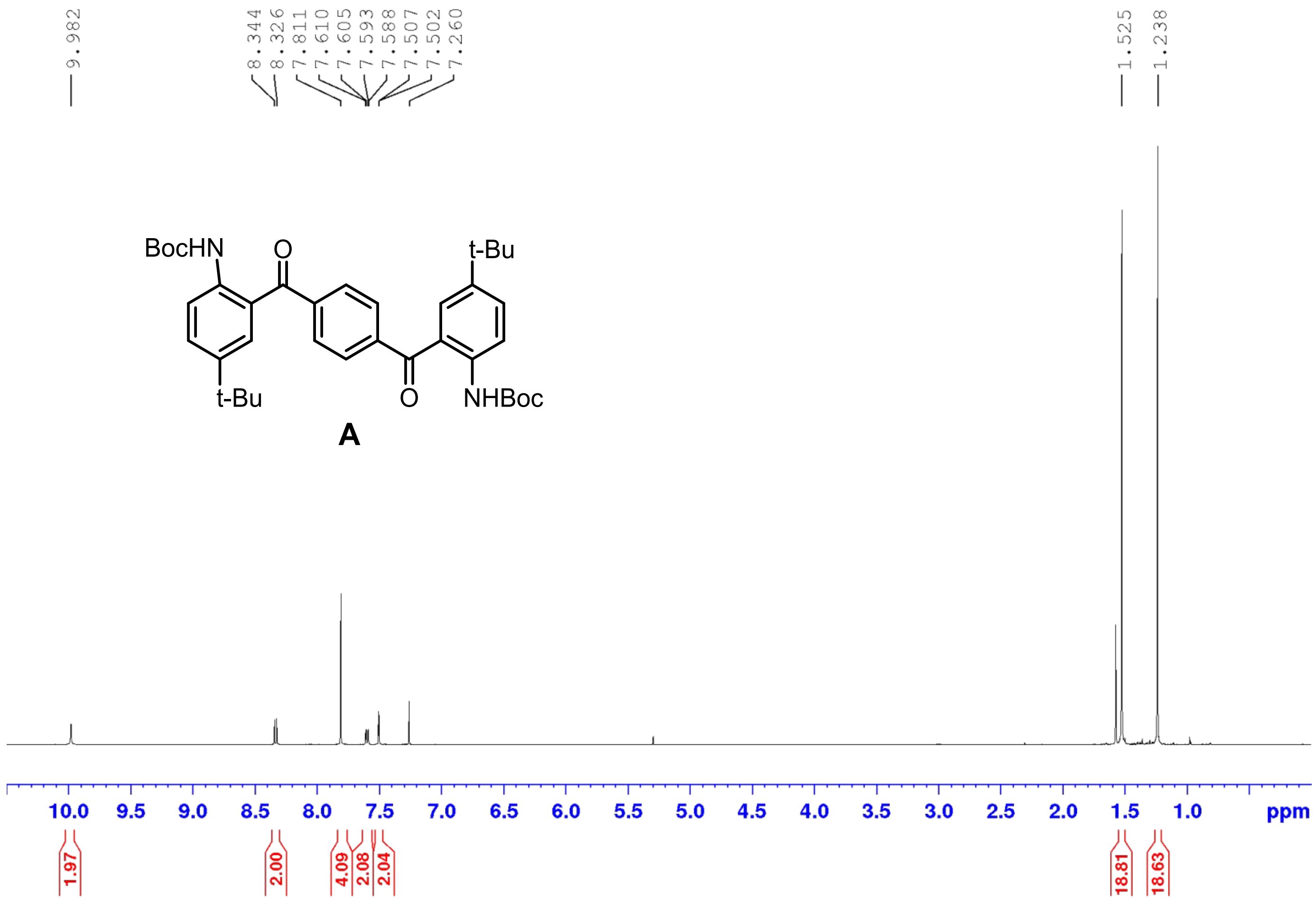

^{1}H NMR spectrum (400 MHz) of compound **A**

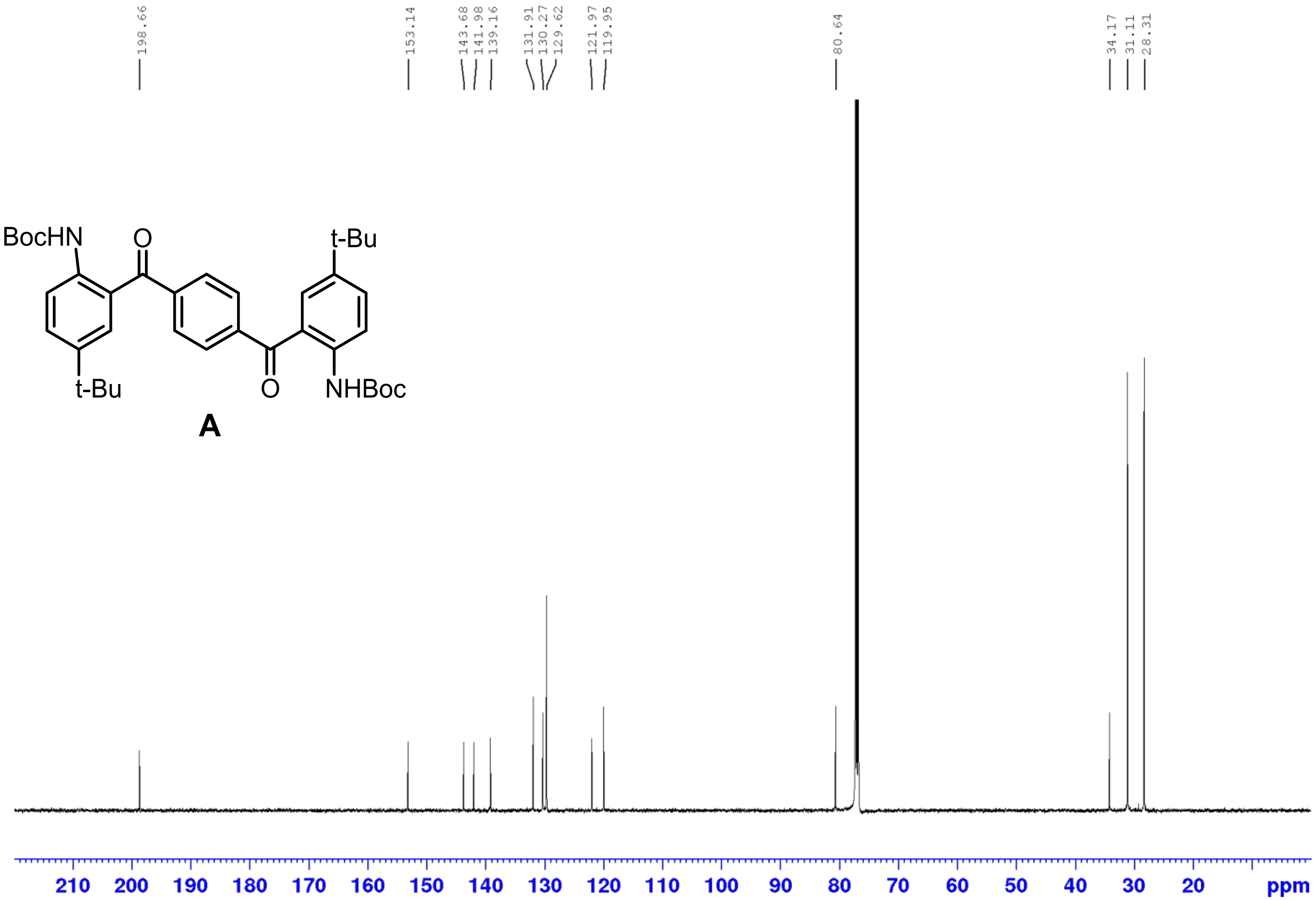

^{13}C NMR spectrum (125 MHz) of compound **A**

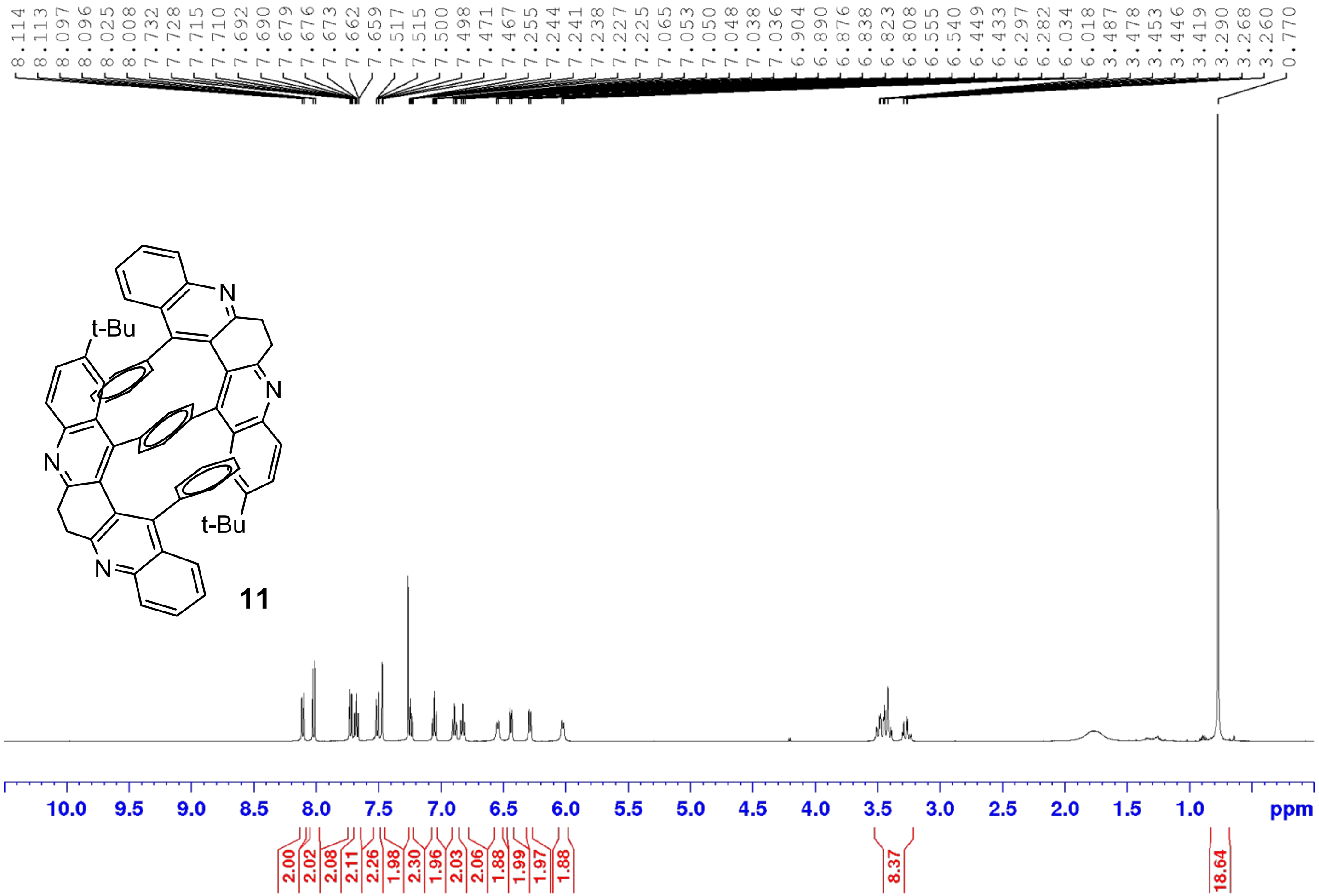

^{1}H NMR spectrum (400 MHz) of compound **11**

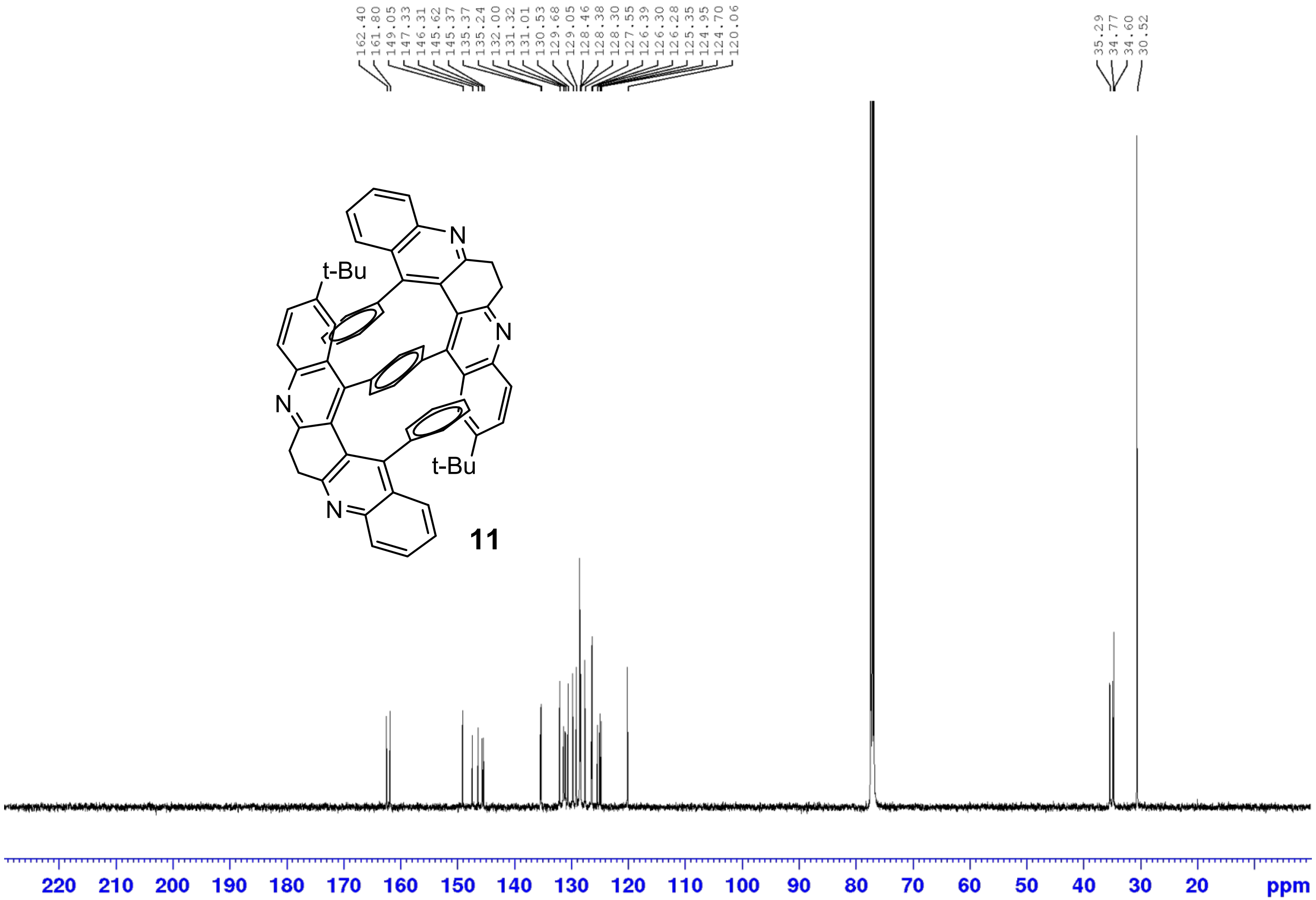

^{13}C NMR spectrum (125 MHz) of compound **11**

t-bu 4ssm new (HR-FAB)

[Mass Spectrum]
Data : 20250826_t-bu 4ssm new-HR-002   Date : 26-Aug-2025 11:31
Sample : t-bu 4ssm new
Note : NBA
Ion Mode : FAB+
RT : 0.00 min   Scan# : (1,2)
Elements : C 1000/0, H 1000/0, N 3/3, O 1/1
Mass Tolerance   : 50mmu
Unsaturation (U.S.) : -0.5 - 10000.0

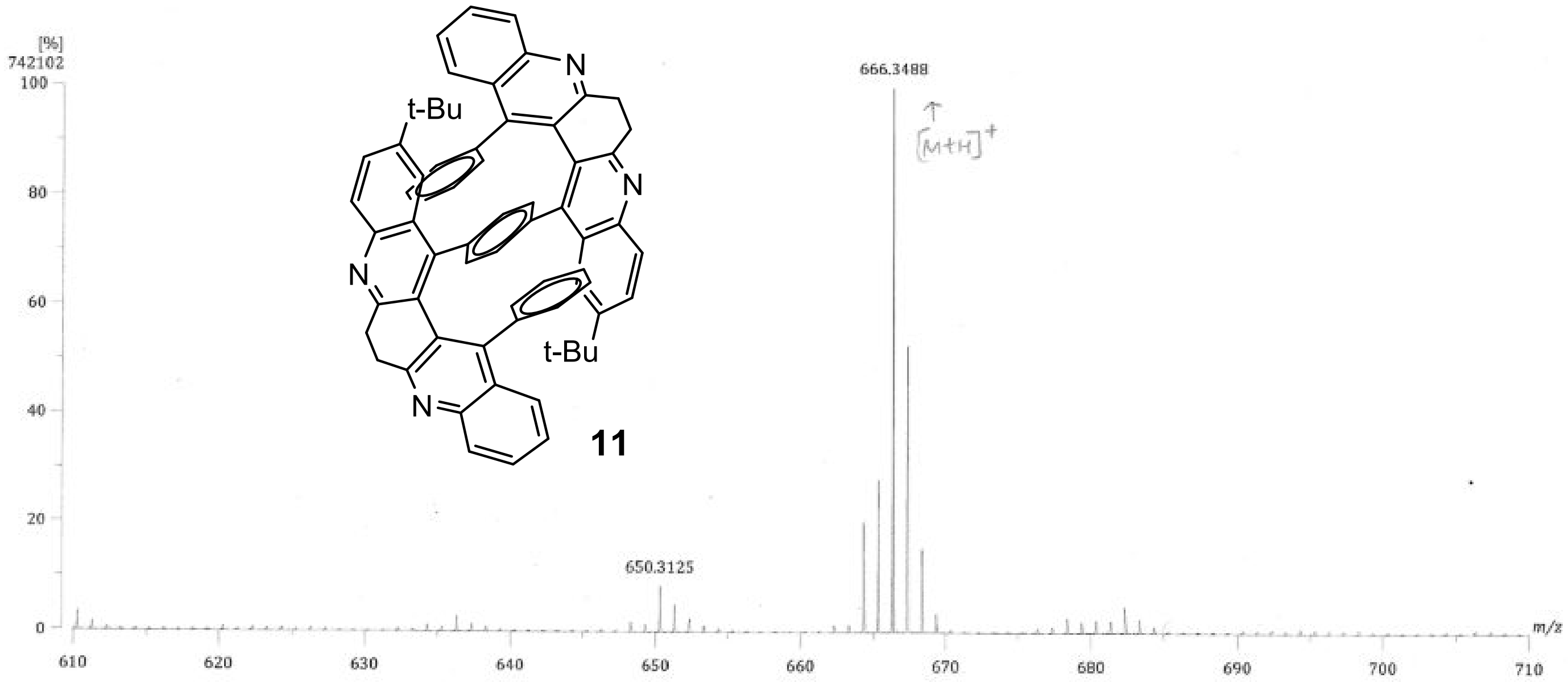

	Observed m/z	Int%					
	666.3488	100.00					
	Estimated m/z	Err[ppm / mmu]	U.S.	C	H	N	O
1	666.3484	+0.5 / +0.4	27.5	47	44	3	1

HRMS data of compound **11**

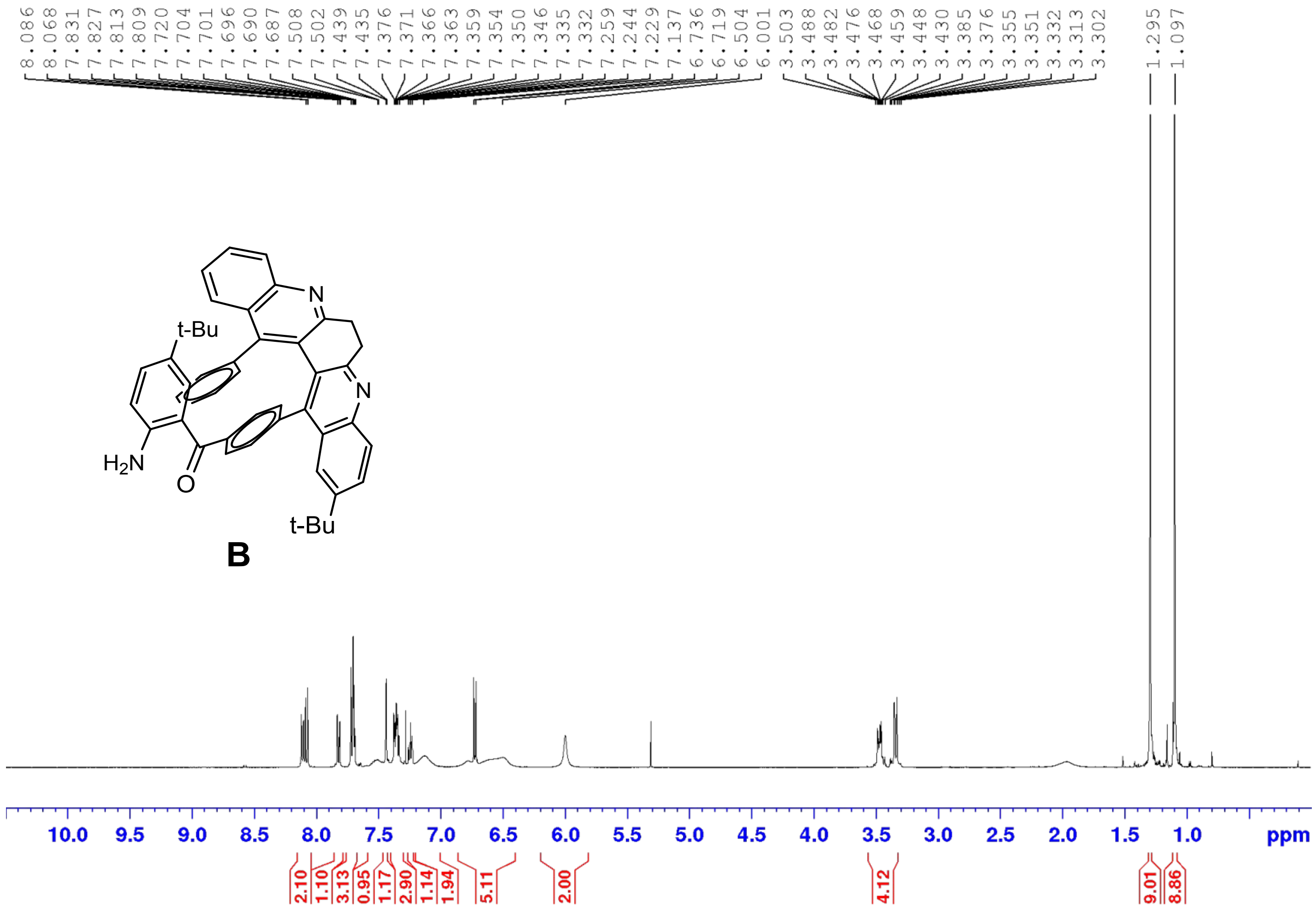

^{1}H NMR spectrum (400 MHz) of compound **B**

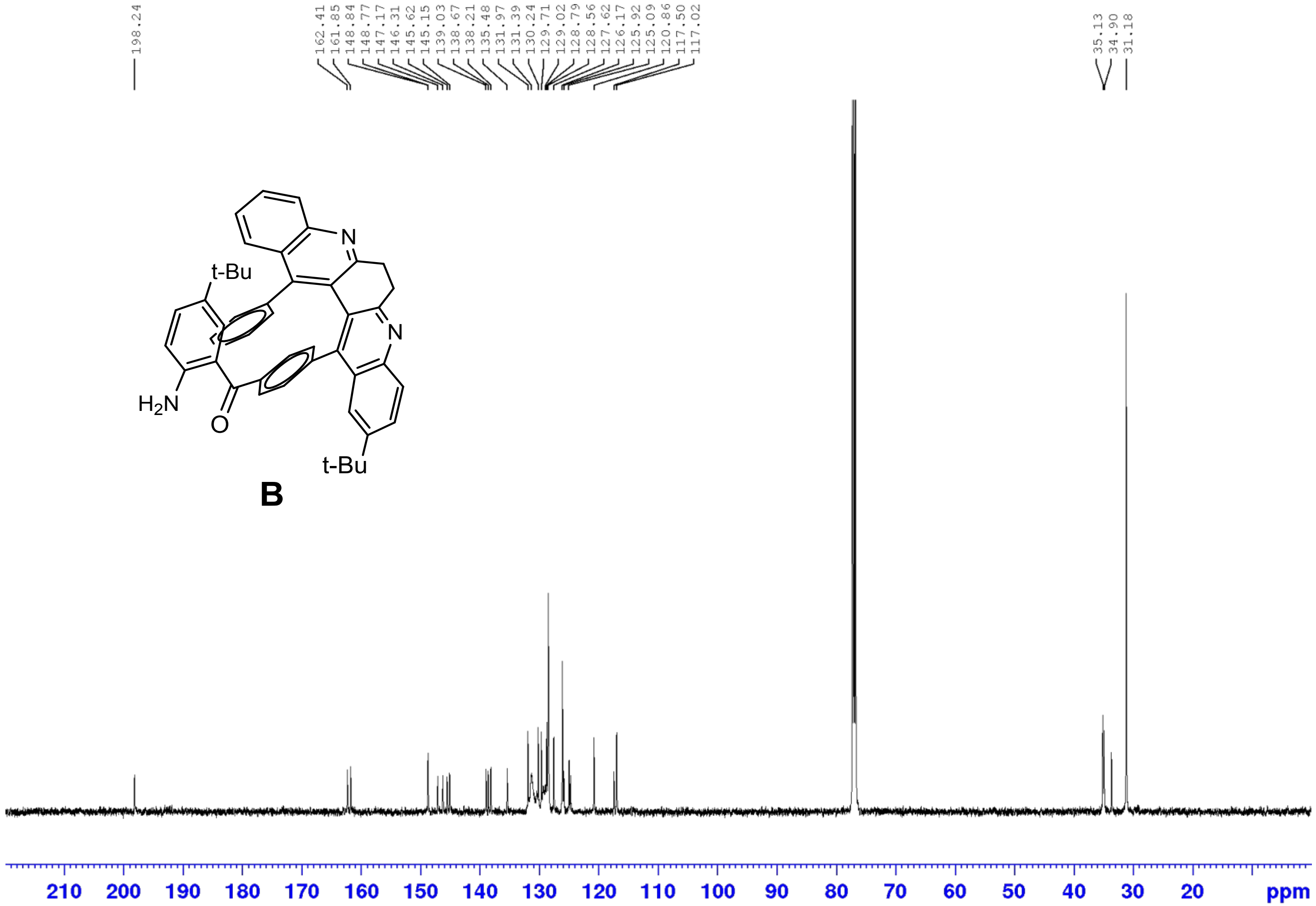

^{13}C NMR spectrum (125 MHz) of compound **B**

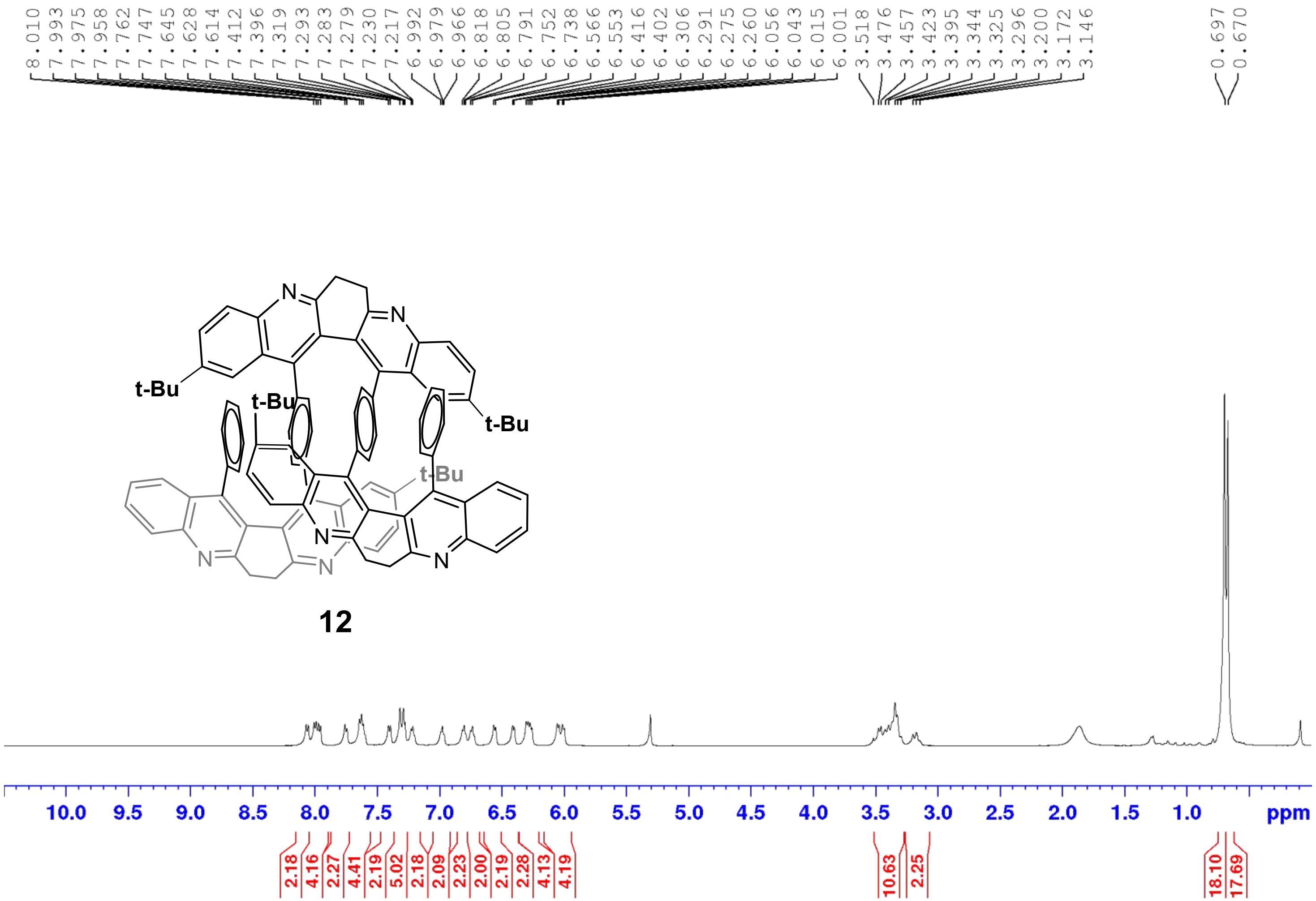

^{1}H NMR spectrum (400 MHz) of compound **12**

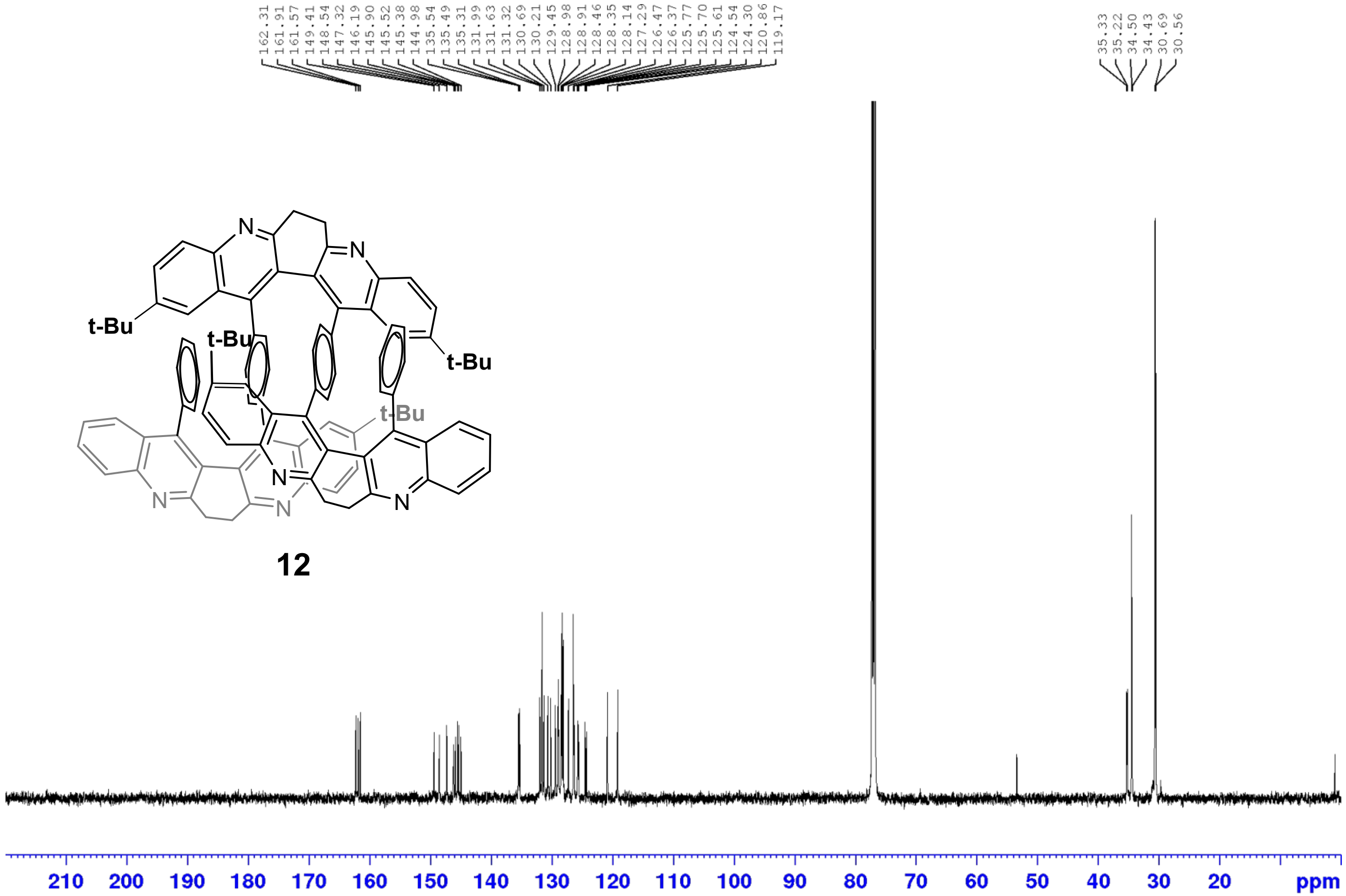

^{13}C NMR spectrum (125 MHz) of compound **12**

[Mass Spectrum]
Data : 20250905_t-Bu 4s-HR-002   Date : 05-Sep-2025 10:37
Sample : t-Bu 4s
Note : NBA
Ion Mode : FAB+
RT : 0.14 min   Scan# : (7,11)
Elements : C 1000/0, H 1000/0, N 6/6
Mass Tolerance   : 50mmu
Unsaturation (U.S.) : -0.5 - 10000.0

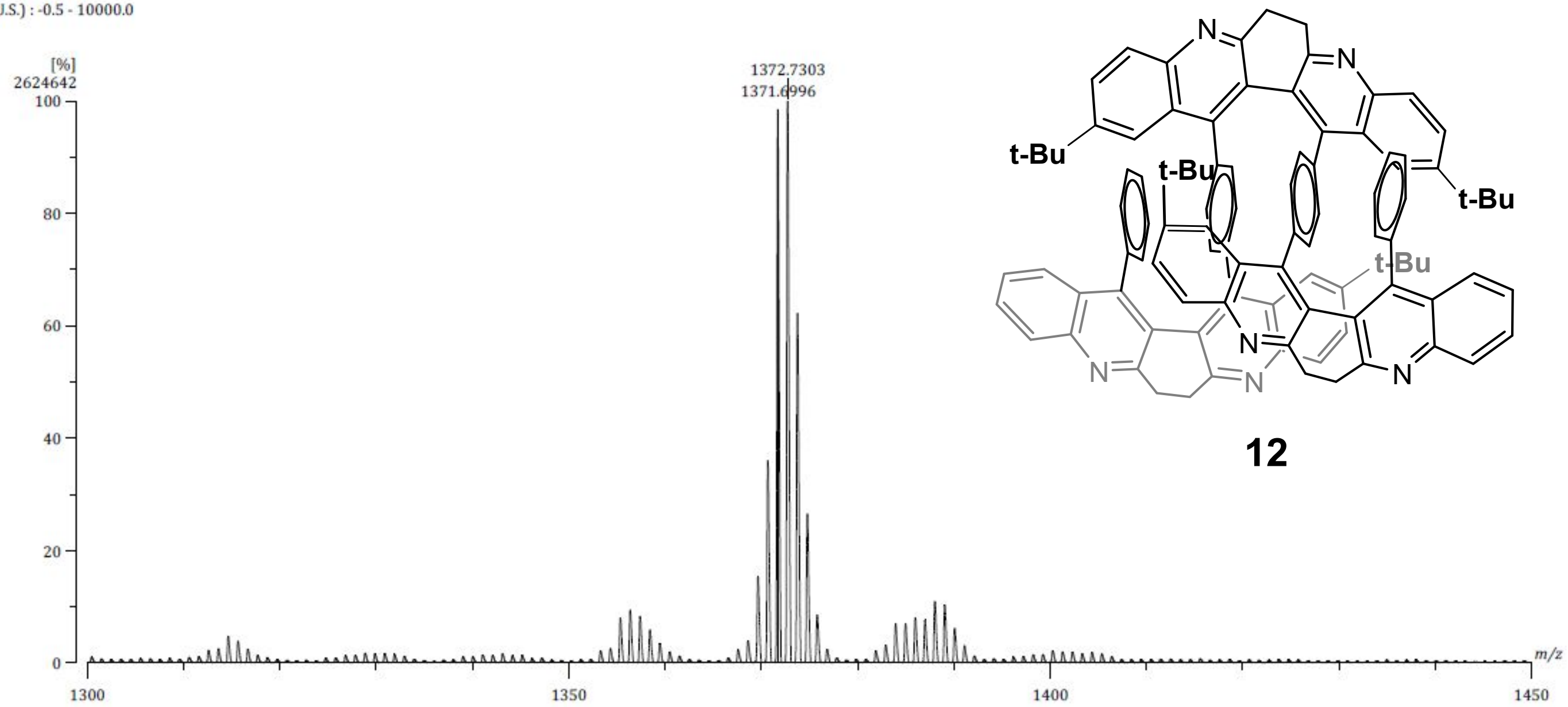

Observed m/z	Int%				
1371.6996	98.52				
Estimated m/z	Err[ppm / mmu]	U.S.	C	H	N
1 1371.6992	+0.3 / +0.4	60.5	100	87	6

HRMS data of compound **12**

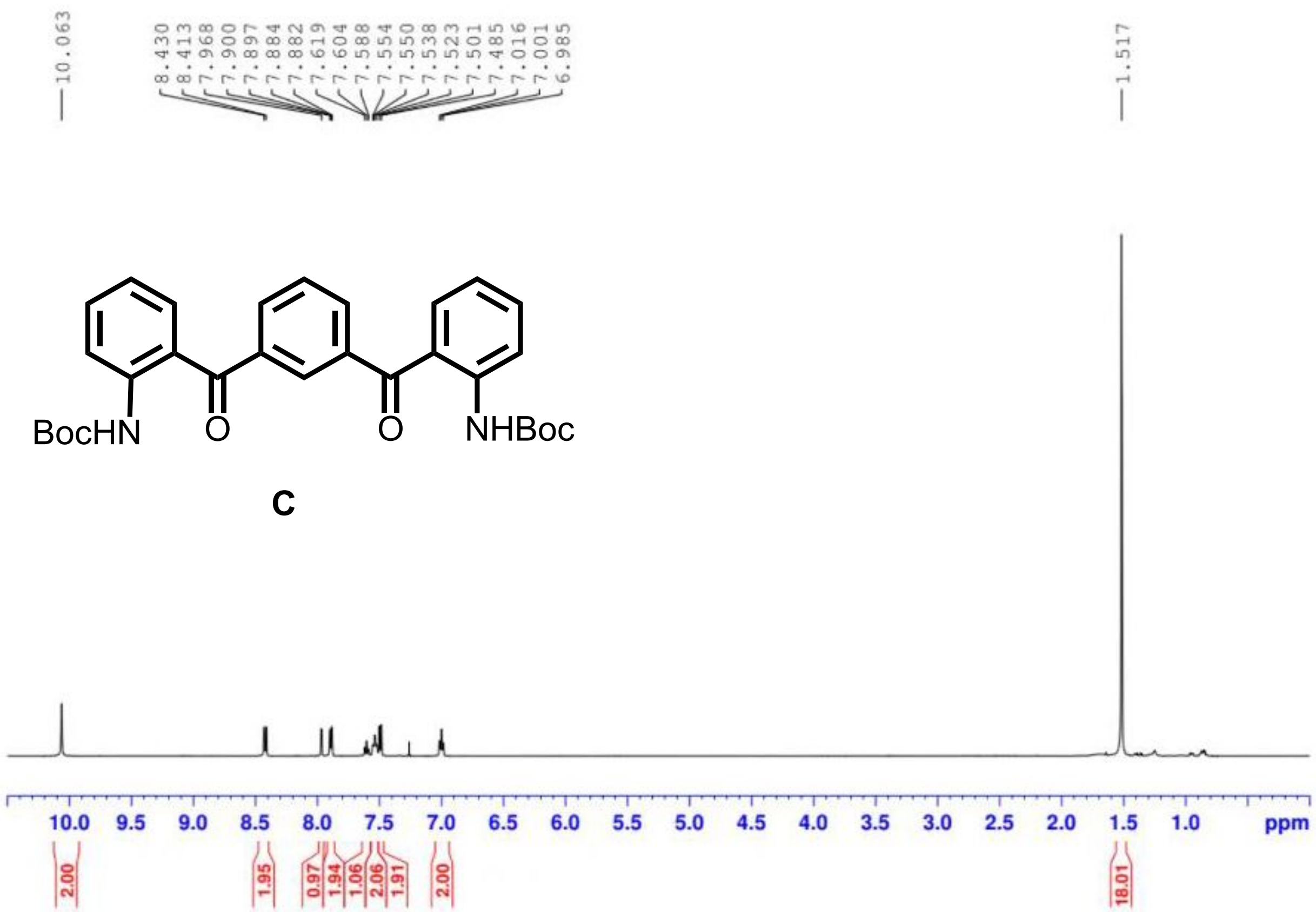

^{1}H NMR spectrum (500 MHz) of compound **C**

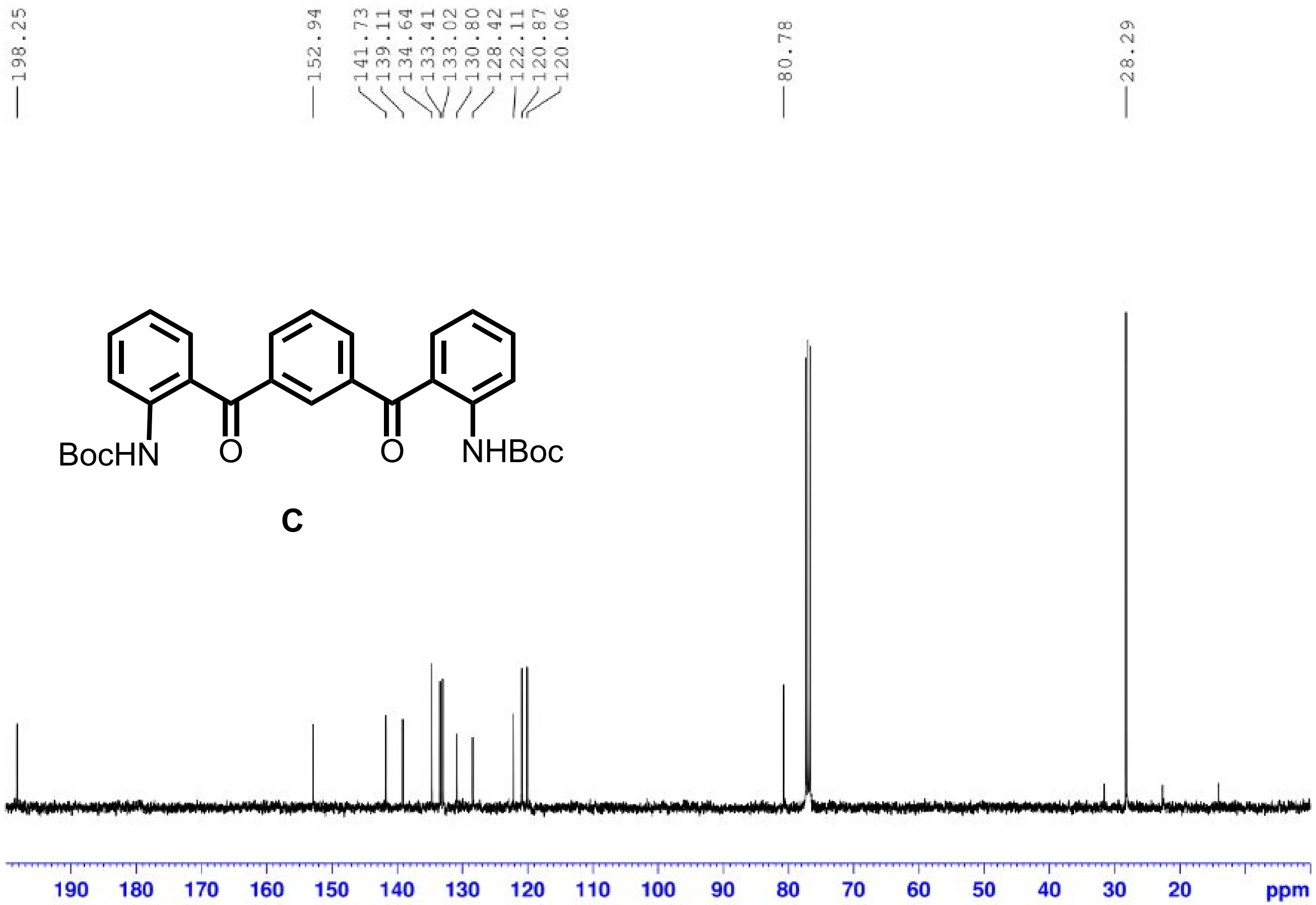

^{13}C NMR spectrum (125 MHz) of compound **C**

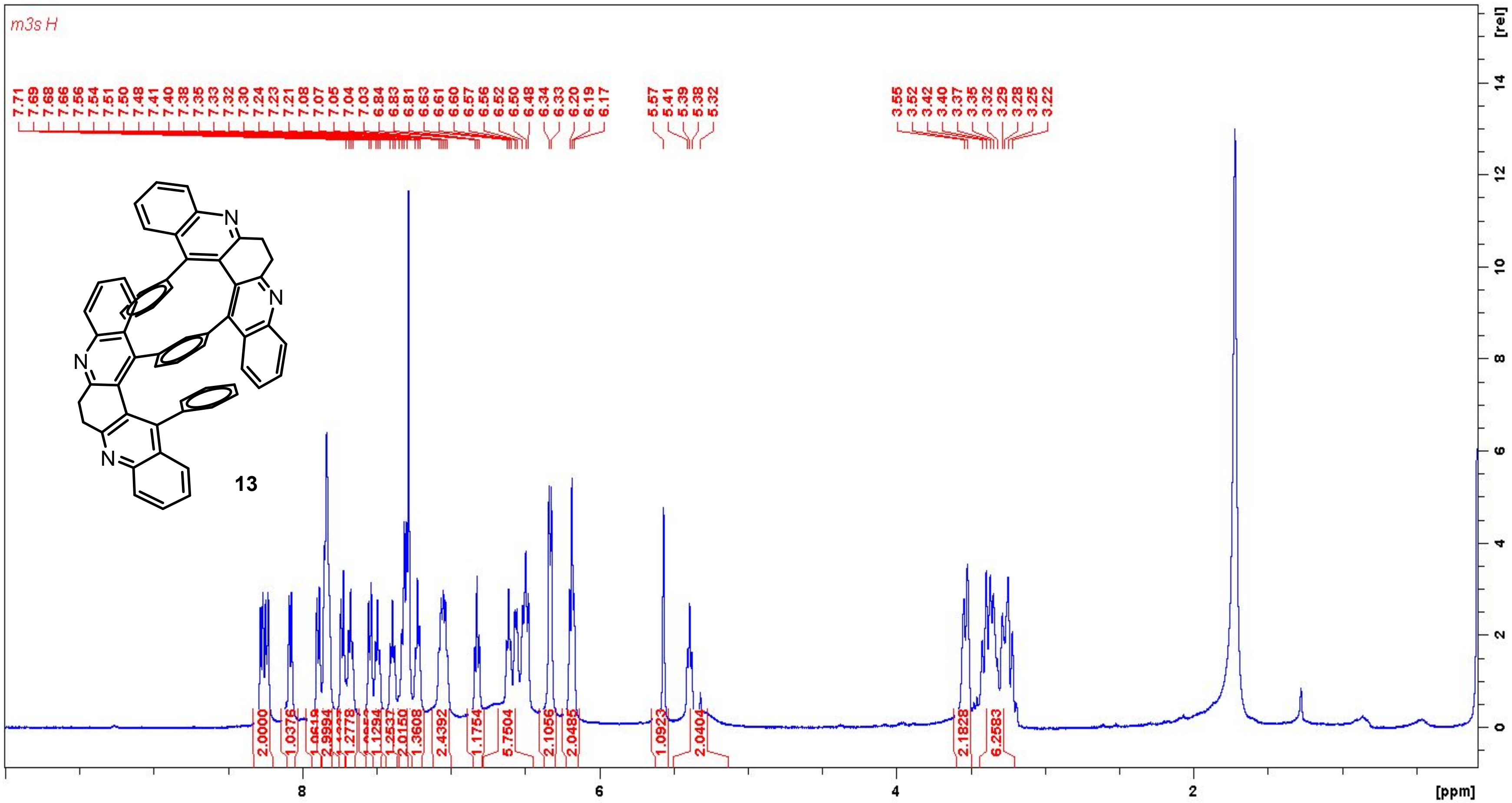

^{1}H NMR spectrum (500 MHz) of compound **13**

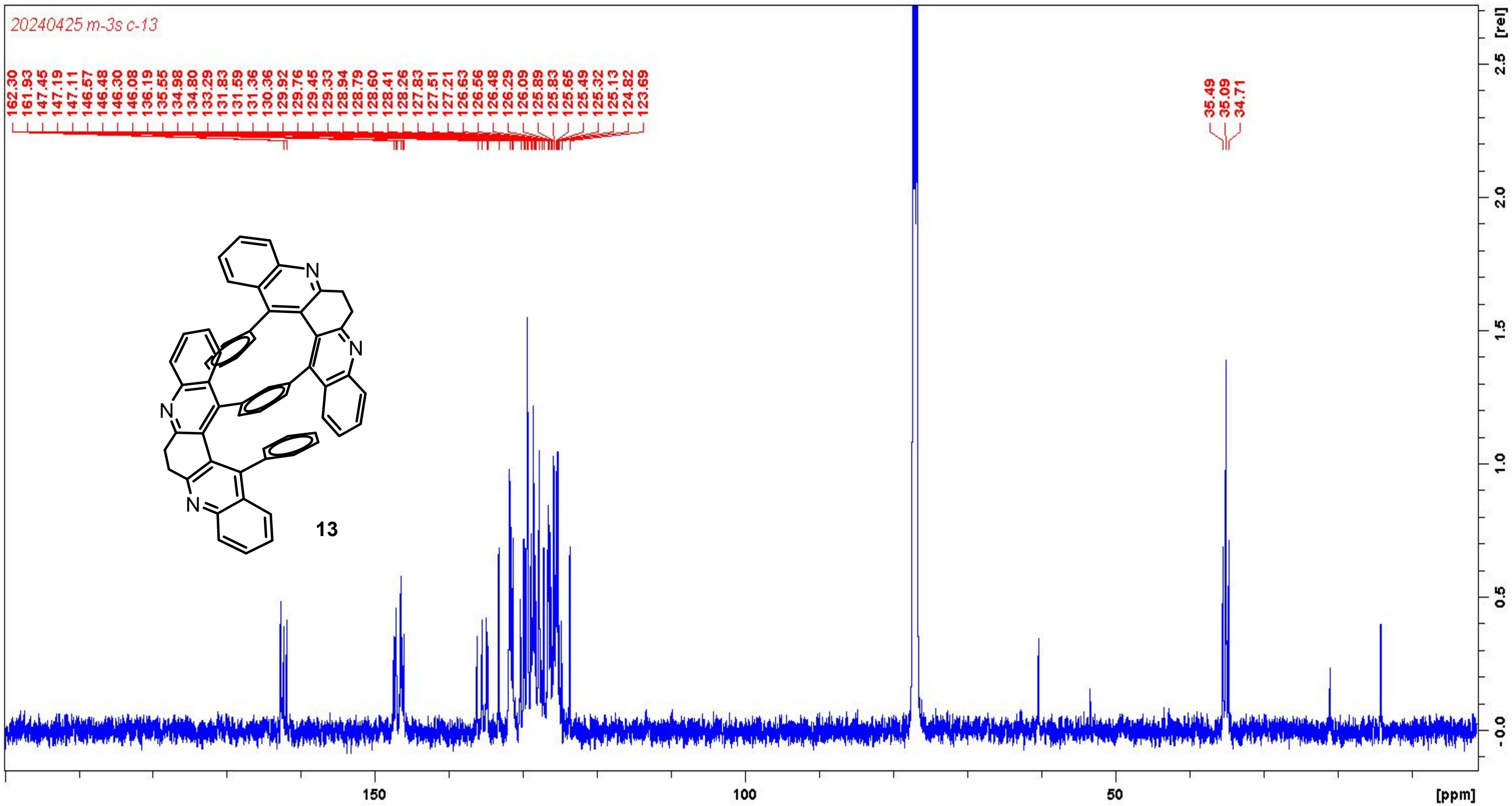

^{13}C NMR spectrum (125 MHz) of compound **13**

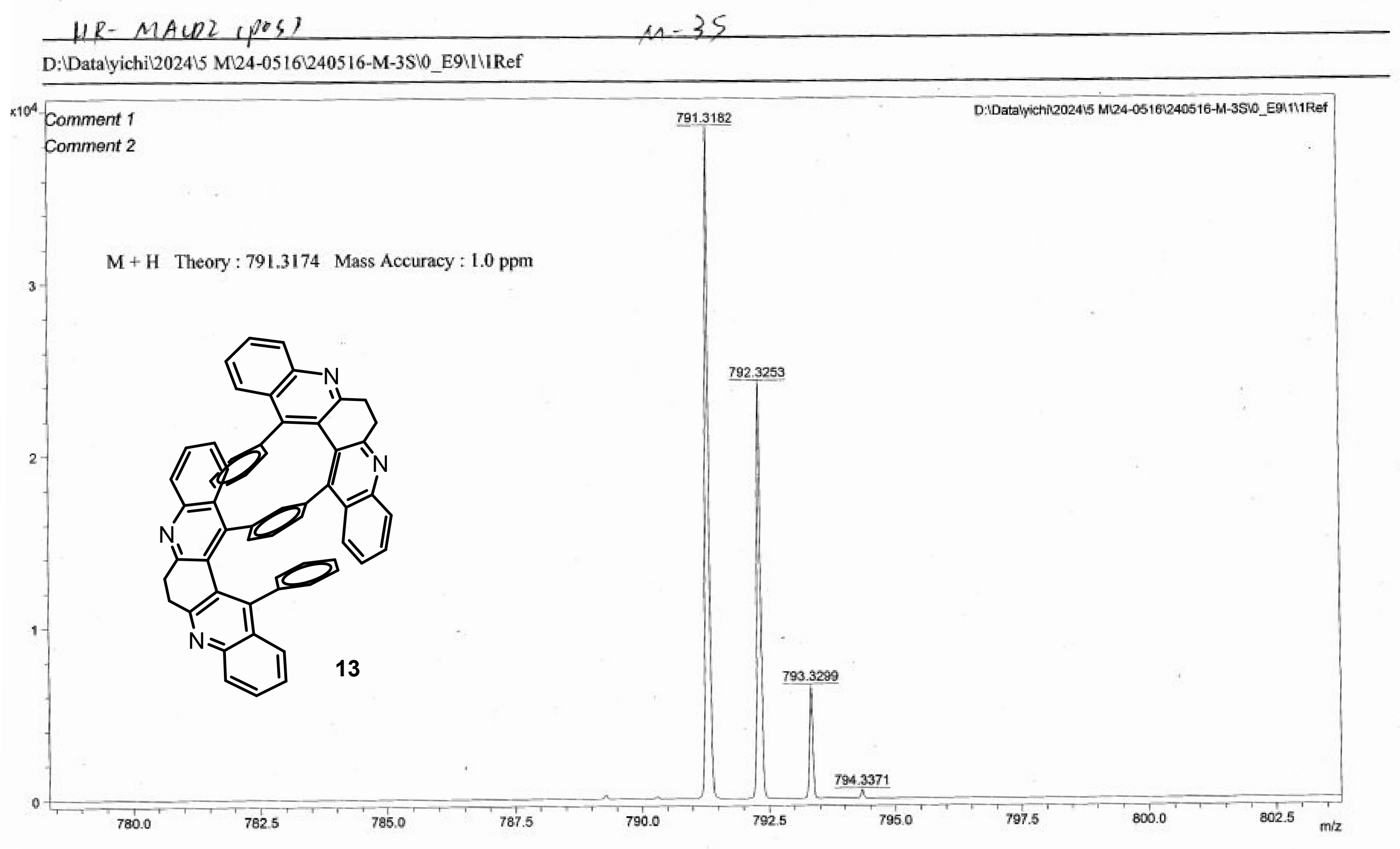

HRMS data of compound **13**

Variable-temperature experiments of 13,14-diphenyl-DHDBPT

(two-layer phenylene stack)

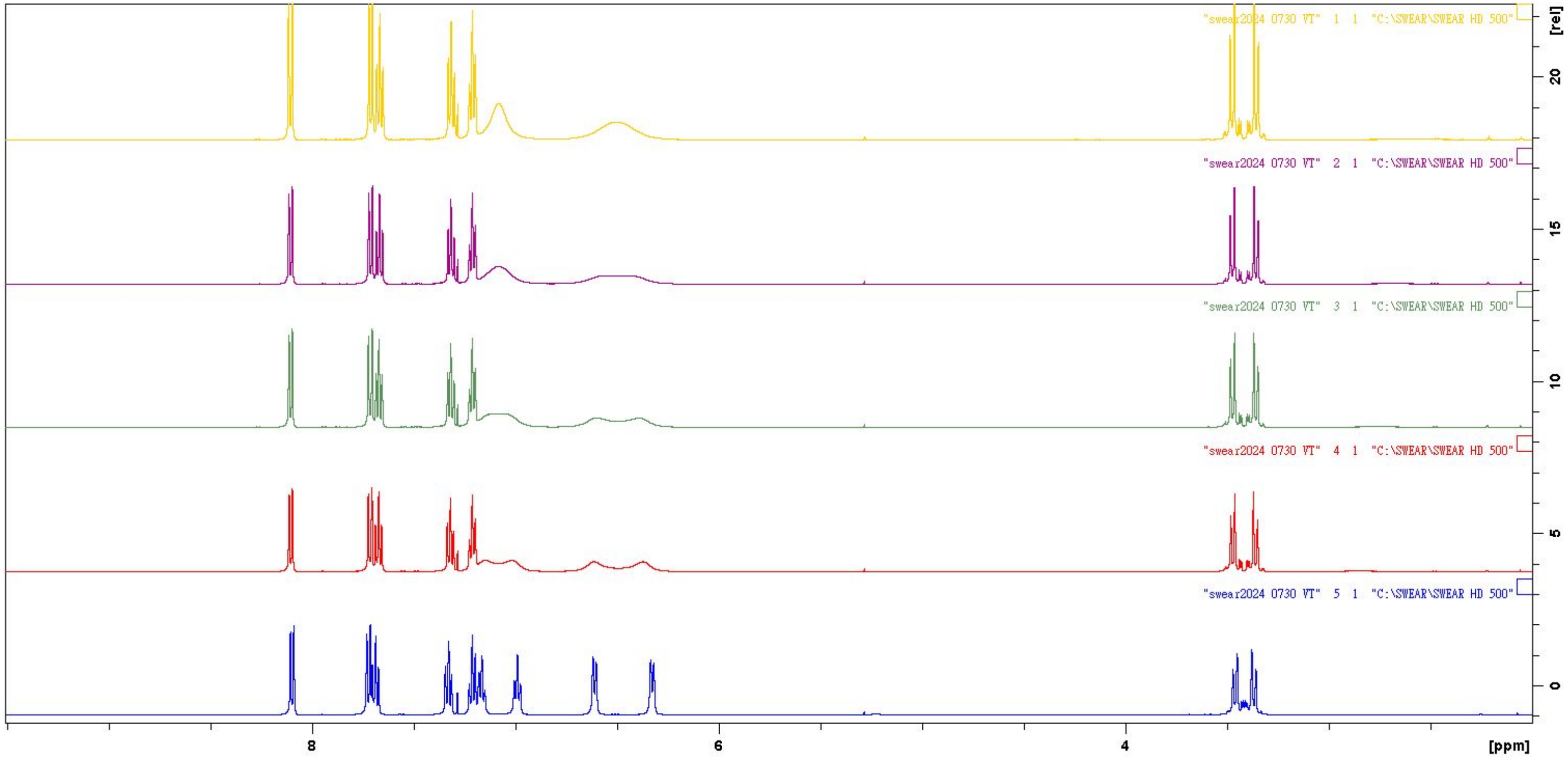

Variable-temperature experiments of compound **3**

(three-layer phenylene stack)

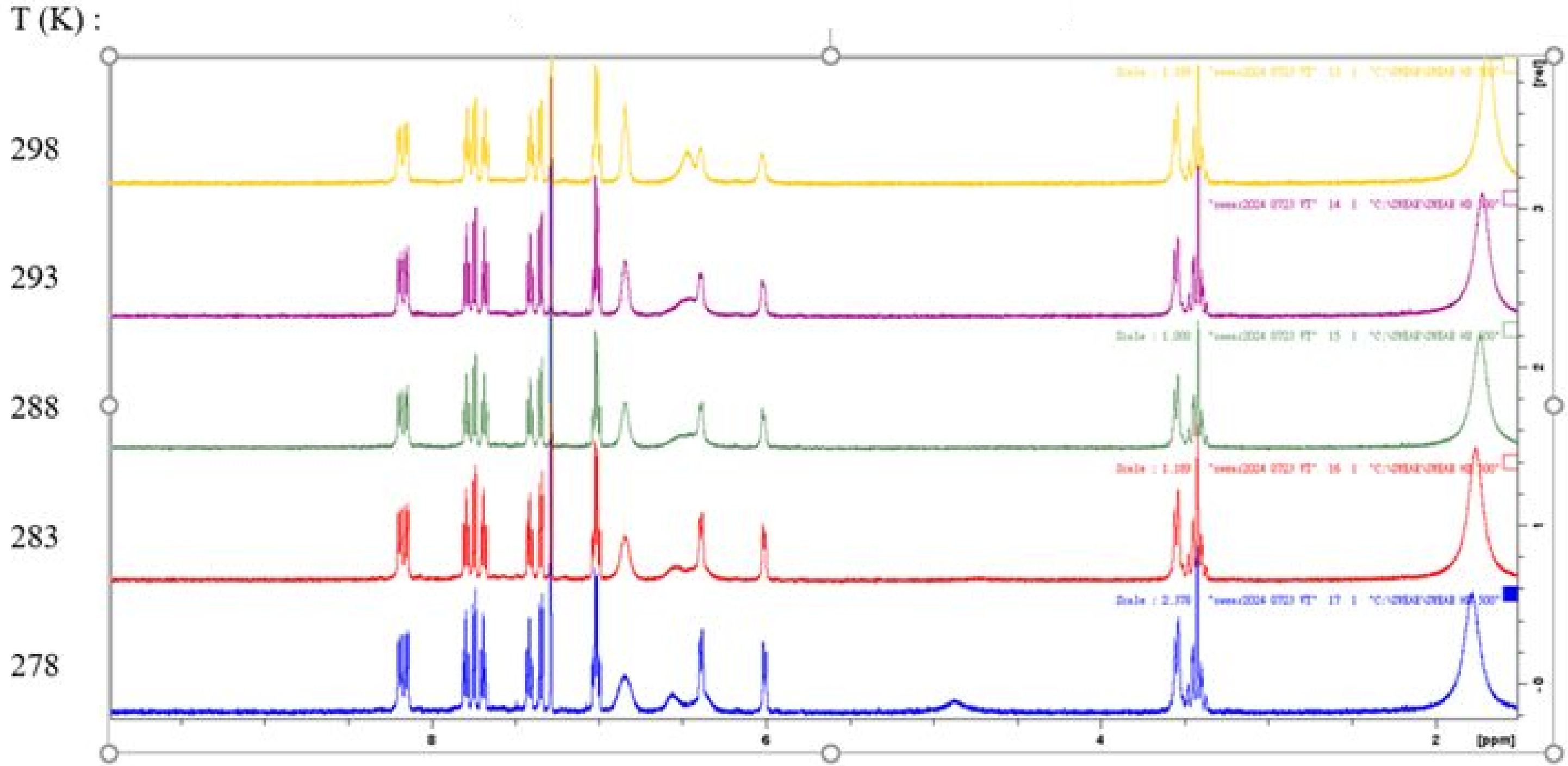

Variable-temperature experiments of compound **5**

(four-layer phenylene stack)

298 K

293 K

288 K

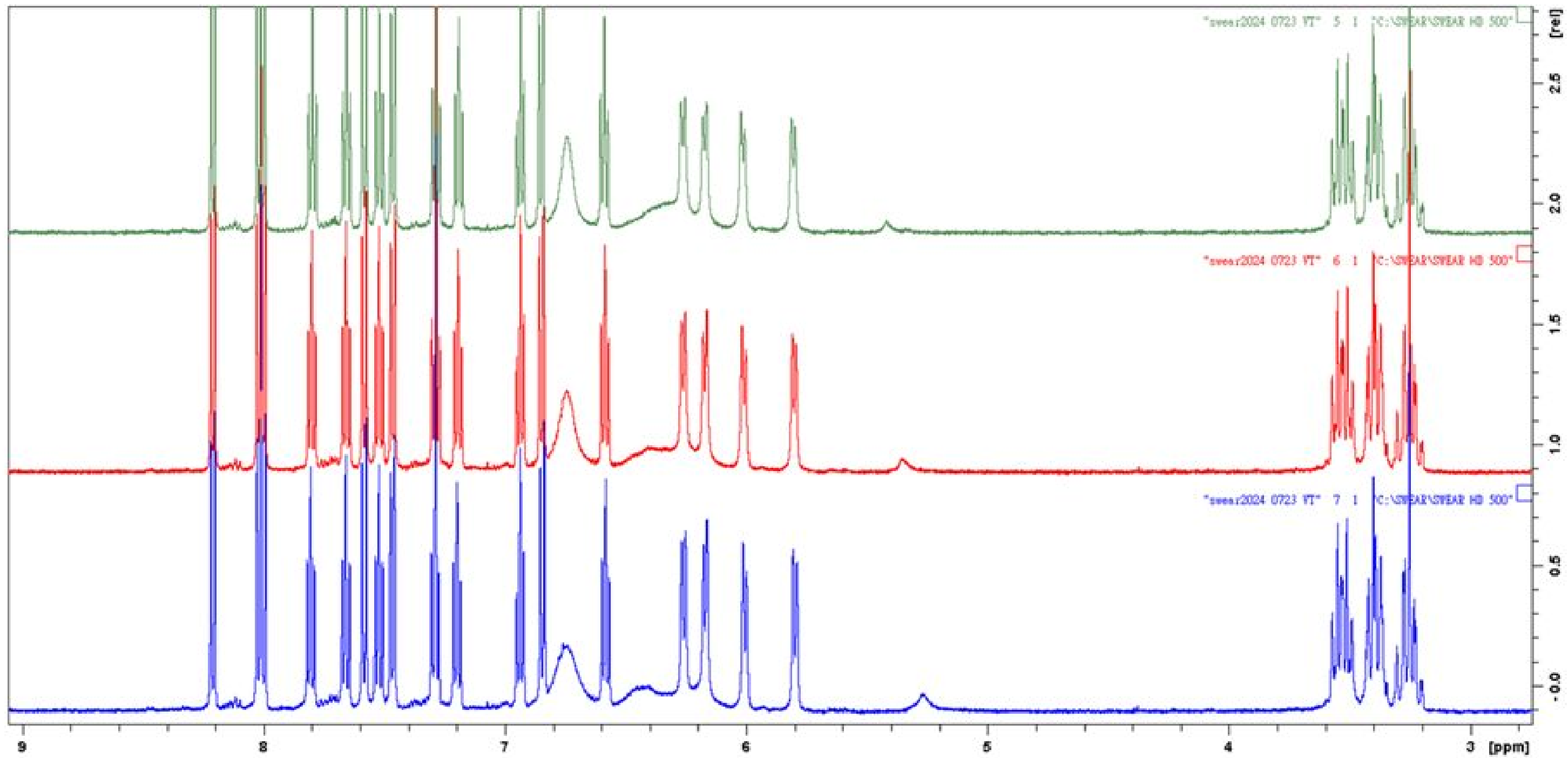

Variable-temperature experiments of compound **7**

(five-layer phenylene stack)

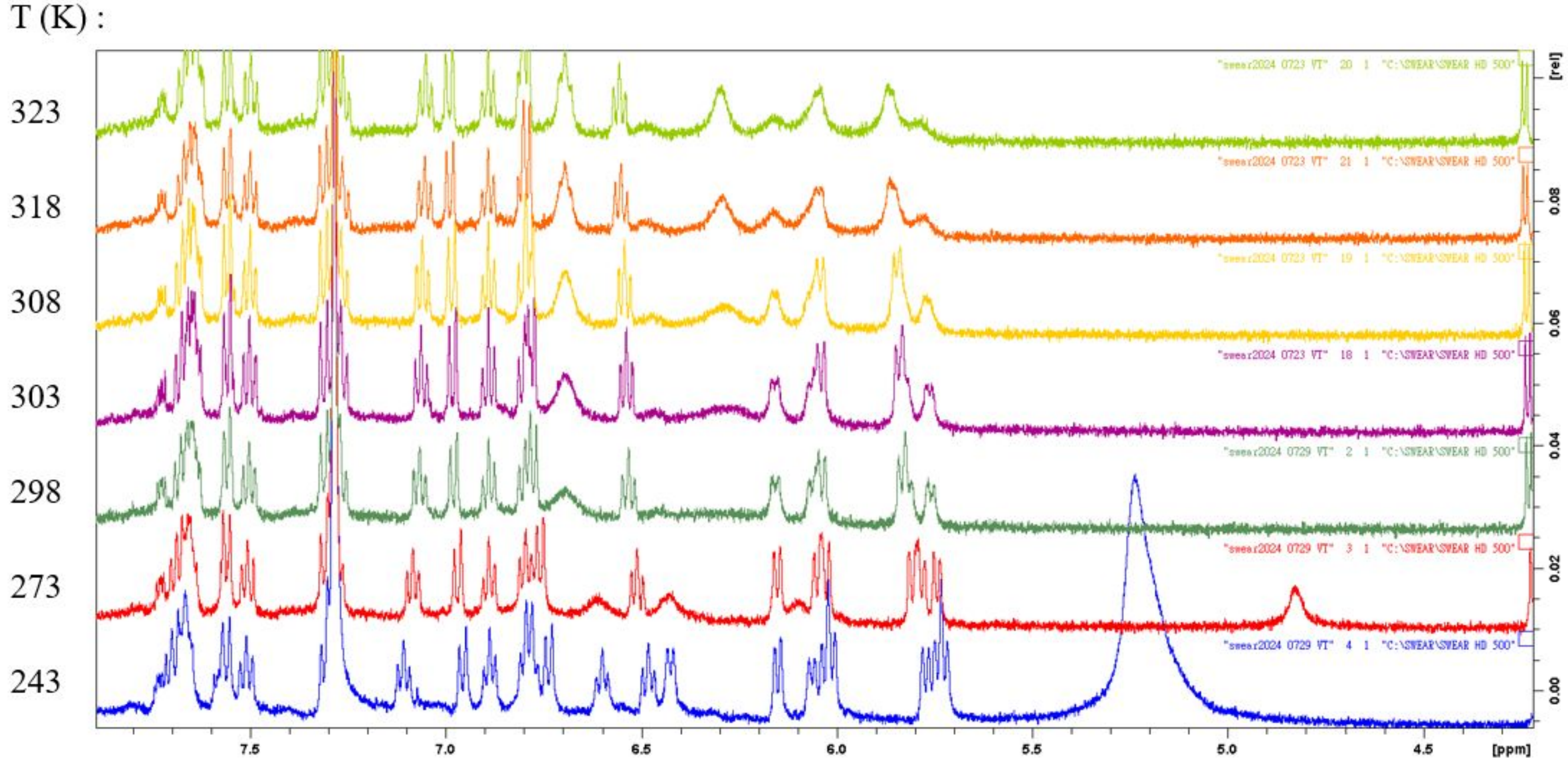

The activation free energies were calculated with the Eyring equation (1) and the relationship between the exchange rate at coalescence temperatures and frequency separation of the exchanging signals (2).

$$k = e^{-\Delta G^*/RT} \times k_b T/ h \text{.........(1)}, \qquad K = \pi\Delta\nu/\sqrt{2} \text{...........(2)}$$

k = rate constant, k_b Boltzmann constant, T = temperature, h = Planck constant

R= gas constant, ΔG^*= activation free energy, $\Delta\nu$ = chemical shift difference between coalescing signals

$$\Delta G^* = RT\,[\ln(k_b T/ h) - \ln(\pi\Delta\nu/\sqrt{2})] \text{................(3)}$$

	**13,14-diphenyl-DHDBPT**	**3**	**5**	**7**
**Coalescence temperature**	**293 K**	**288 K**	**293 K**	**303 K**
$\Delta\nu$	**120.37 Hz**	**122.37 Hz**	**174.82 Hz**	**182.31 Hz**
**Calculated rotational free energy barrier**	**13.4 kcal mol^{-1}**	**13.6 kcal mol^{-1}**	**13.7 kcal mol^{-1}**	**14.1 kcal mol^{-1}**

Table S2: Rotational barriers of outer phenyl groups for two-layer to five-layer systems

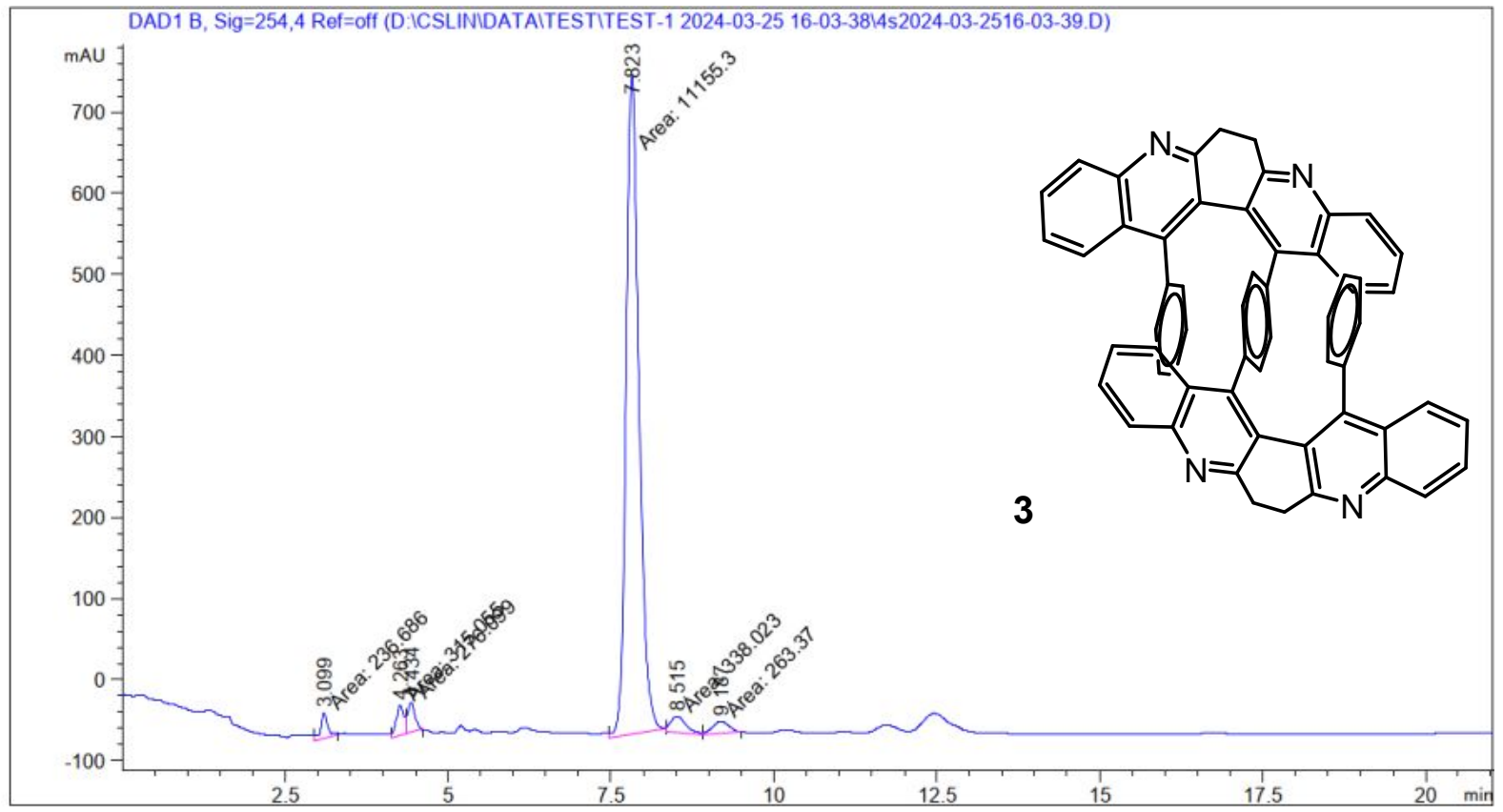

Figure S1. Chiral HPLC chromatogram of compound **3**

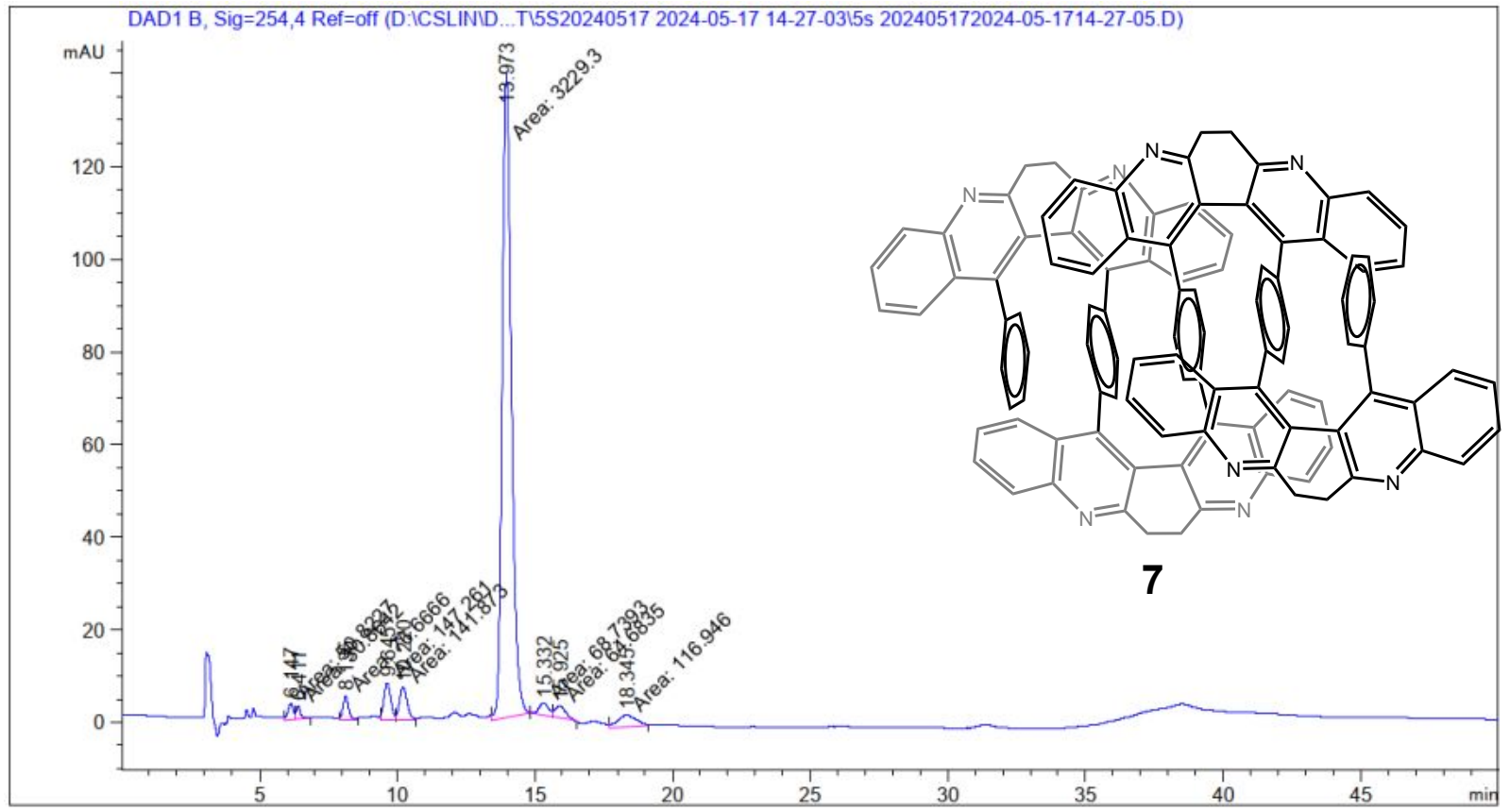

Figure S2. Chiral HPLC chromatogram of compound **7**

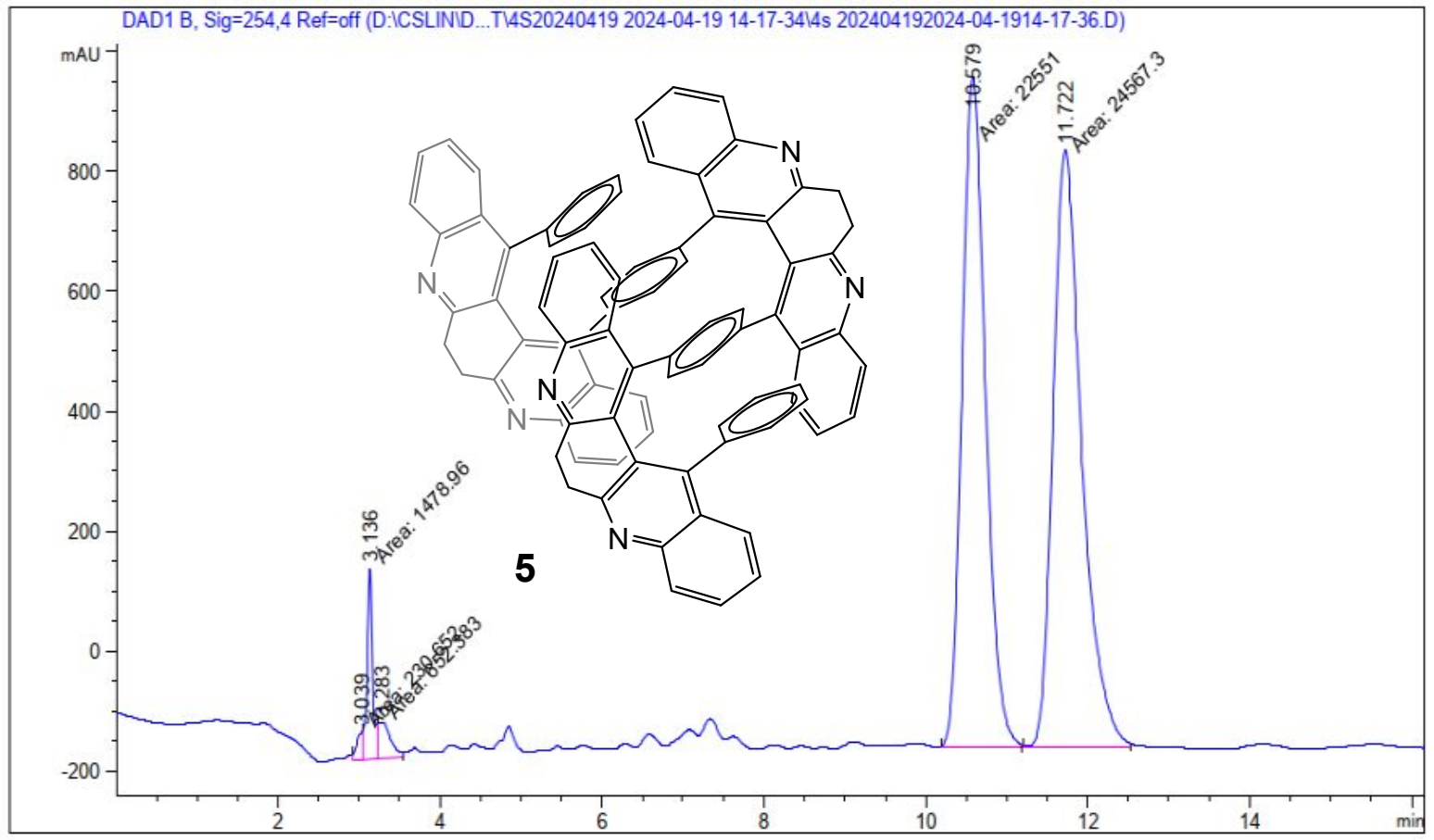

Figure S3. Chiral HPLC chromatogram of compound **5**

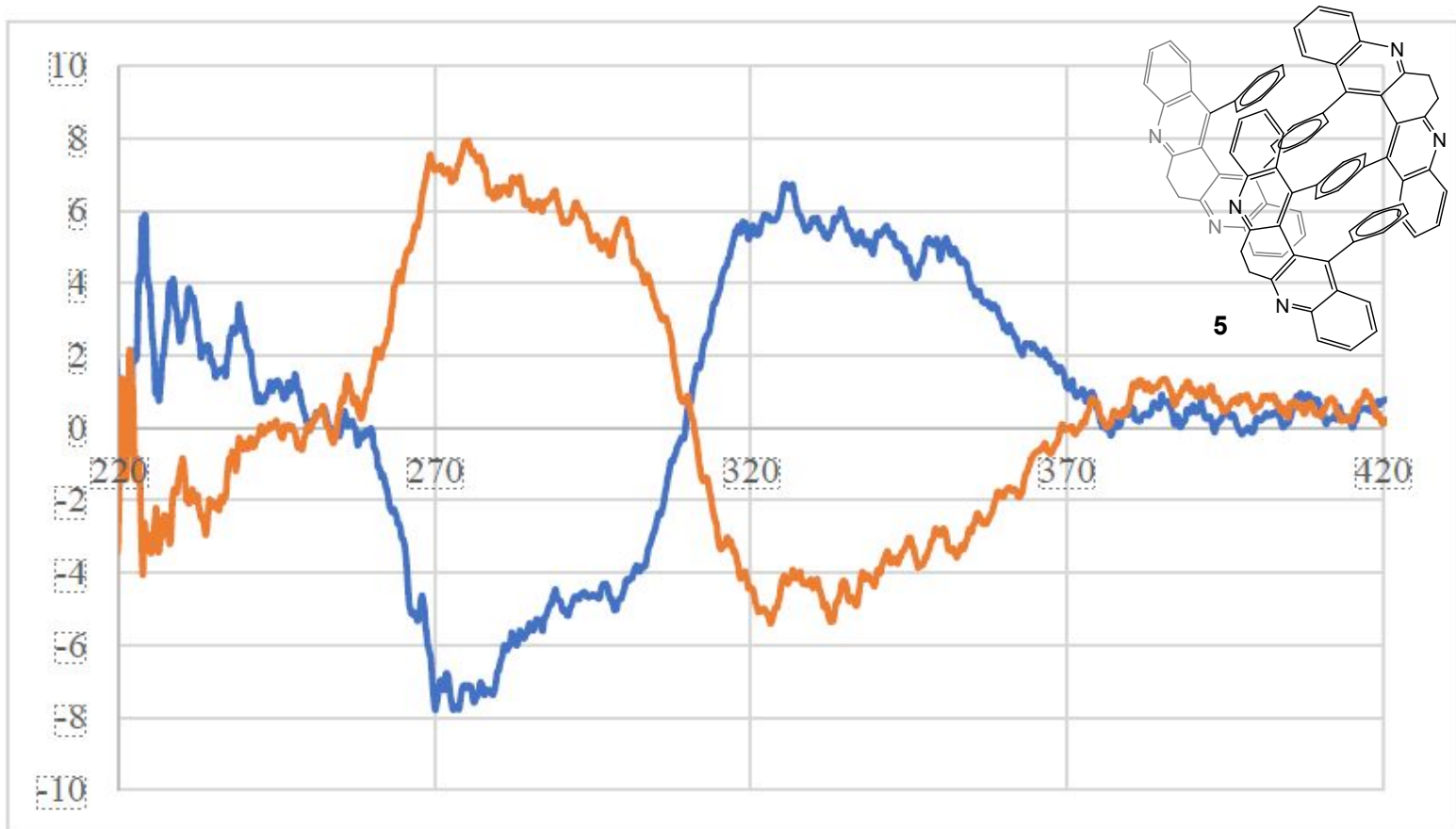

Figure S4. Circular dichroism spectra of both enantiomers of **5**

Compound **11**

Empirical formula	$C_{35} H_{31} Cl_2 N_2$
Formula weight	550.52
Temperature	100.0(2) K
Wavelength	1.54178 Å
Crystal system	Triclinic
Space group	P -1
Unit cell dimensions	a = 11.0423(4) Å α= 100.7920(10)°.
	b = 11.5293(4) Å β= 104.5830(10)°.
	c = 13.0441(5) Å γ = 111.4910(10)°.
Volume	1421.56(9) Å3
Z	2
Density (calculated)	1.286 Mg/m3
Absorption coefficient	2.250 mm-1
F(000)	578
Crystal size	0.311 x 0.140 x 0.126 mm3
Theta range for data collection	4.739 to 68.371°.
Index ranges	-13<=h<=13, -13<=k<=13, -15<=l<=15
Reflections collected	41953
Independent reflections	5190 [R(int) = 0.1026]
Completeness to theta =	67.679° 99.7 %
Absorption correction	Numerical
Max. and min. transmission	1 and 0.7787
Refinement method	Full-matrix least-squares on F2
Data / restraints / parameters	5190 / 311 / 355
Goodness-of-fit on F2	1.053
Final R indices [I>2sigma(I)]	R1 = 0.0571, wR2 = 0.1475
R indices (all data)	R1 = 0.0576, wR2 = 0.1481
Extinction coefficient	n/a
Largest diff. peak and hole	0.497 and -0.650 e.Å-3

Compound **12**

Empirical formula	$C_{101} H_{87} Cl N_6$
Formula weight	1420.21
Temperature	100(2) K
Wavelength	1.54178 Å
Crystal system	Monoclinic
Space group	P 21/n
Unit cell dimensions	a = 20.5060(8) Å α= 90°.
	b = 17.8213(8) Å β= 96.670(3)°.
	c = 23.5116(11) Å γ = 90°.
Volume	8534.0(6) Å3
Z	4
Density (calculated)	1.105 Mg/m3
Absorption coefficient	0.769 mm-1
F(000)	3008
Crystal size	0.107 x 0.047 x 0.029 mm3
Theta range for data collection	3.040 to 67.495°.
Index ranges	-24<=h<=22, -21<=k<=18, -28<=l<=28
Reflections collected	72990
Independent reflections	15357 [R(int) = 0.1474]
Completeness to thet	a = 67.495° 99.7 %
Absorption correction	Semi-empirical from equivalents
Max. and min. transmission	0.9583 and 0.5102
Refinement method	Full-matrix least-squares on F2
Data / restraints / parameters	15357 / 720 / 985
Goodness-of-fit on F2	1.030
Final R indices [I>2sigma(I)]	R1 = 0.1055, wR2 = 0.2796
R indices (all data)	R1 = 0.1666, wR2 = 0.3293
Extinction coefficient	n/a
Largest diff. peak and hole	0.836 and -0.900 e.Å-3

Compound **13**

Empirical formula	$C_{60} H_{40} Cl_6 N_4$	
Formula weight	1029.66	
Temperature	100.0(2) K	
Wavelength	1.54178 Å	
Crystal system	Monoclinic	
Space group	P 21/n	
Unit cell dimensions	a = 10.8921(9) Å	α= 90°.
	b = 24.2682(19) Å	β= 97.490(3)°.
	c = 18.7320(15) Å	γ = 90°.
Volume	4909.2(7) Å3	
Z	4	
Density (calculated)	1.393 Mg/m3	
Absorption coefficient	3.549 mm-1	
F(000)	2120	
Crystal size	0.310 x 0.289 x 0.268 mm3	
Theta range for data collection	2.996 to 68.600°.	
Index ranges	-13<=h<=13, -29<=k<=29, -22<=l<=22	
Reflections collected	98190	
Independent reflections	9002 [R(int) = 0.0565]	
Completeness to theta =	67.679° 99.9 %	
Absorption correction	Numerical	
Max. and min. transmission	1 and 0.4474	
Refinement method	Full-matrix least-squares on F2	
Data / restraints / parameters	9002 / 648 / 668	
Goodness-of-fit on F2	1.087	
Final R indices [I>2sigma(I)]	R1 = 0.0548, wR2 = 0.1372	
R indices (all data) R1 =	0.0553, wR2 = 0.1375	
Extinction coefficient	0.00166(13)	
Largest diff. peak and hole	1.002 and -1.352 e.Å-3	

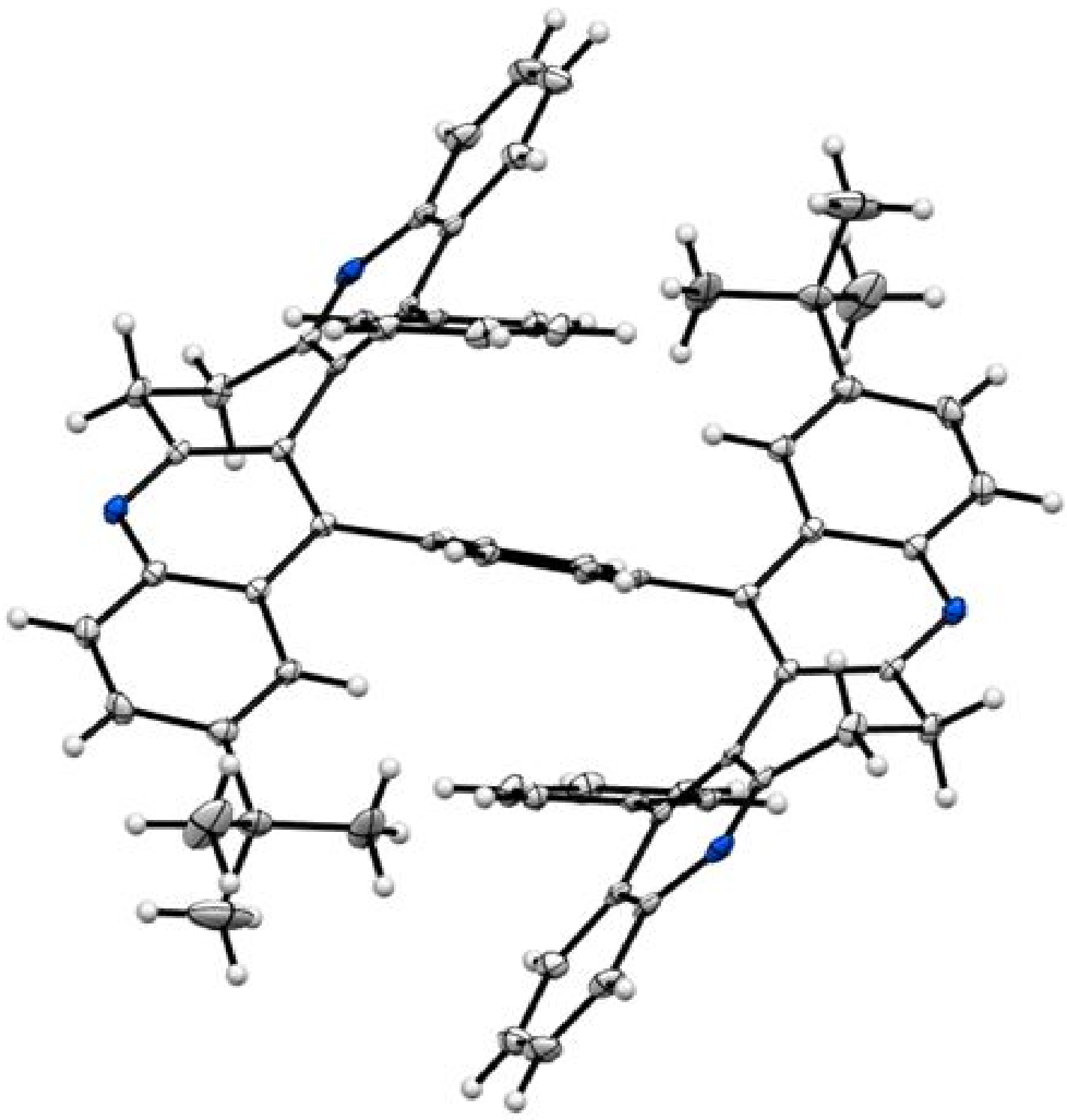

Figure S5: ORTEP graph of compound **11** (thermal ellipsoids at 50% probability)

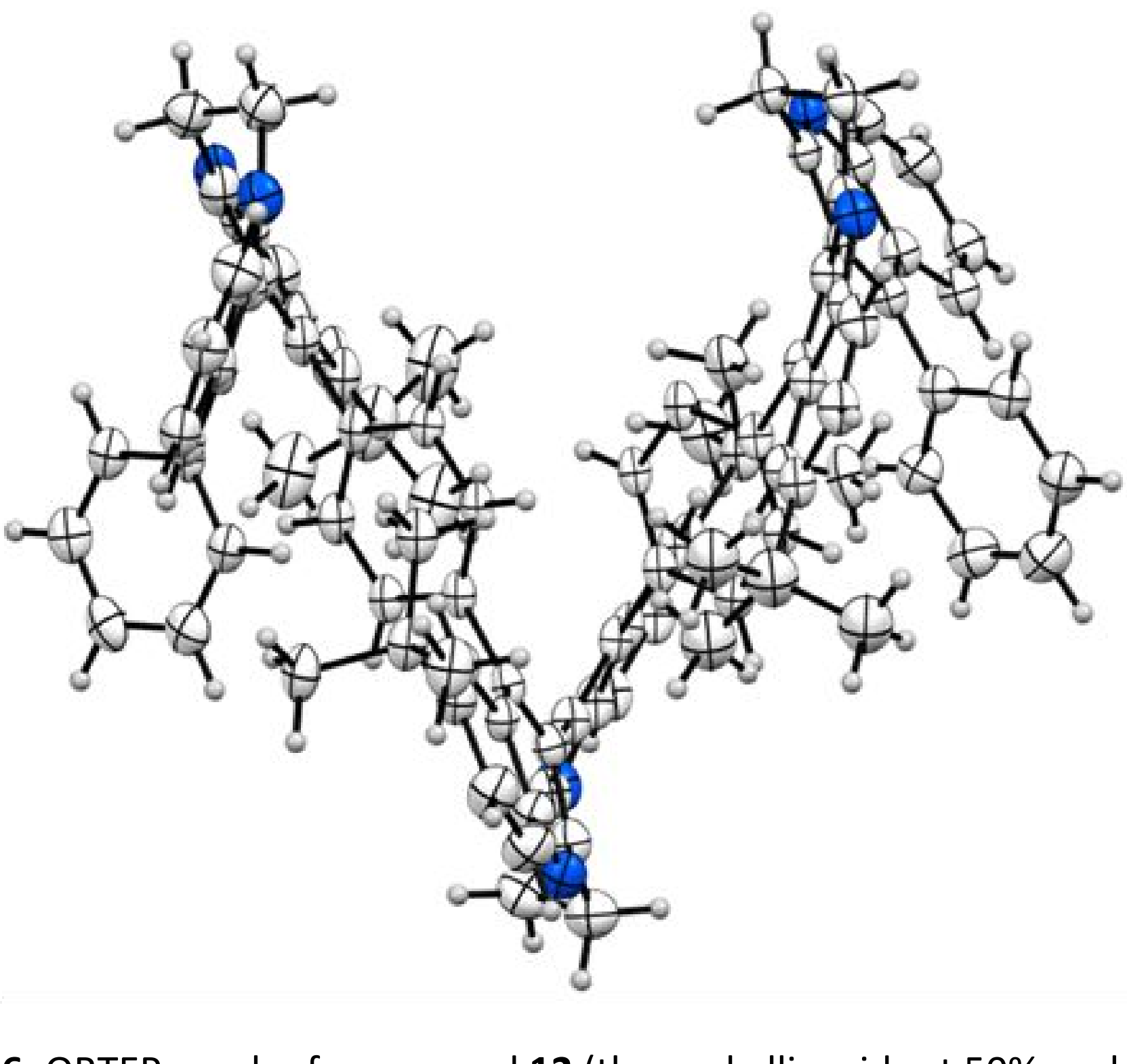

Figure S6: ORTEP graph of compound **12** (thermal ellipsoids at 50% probability)

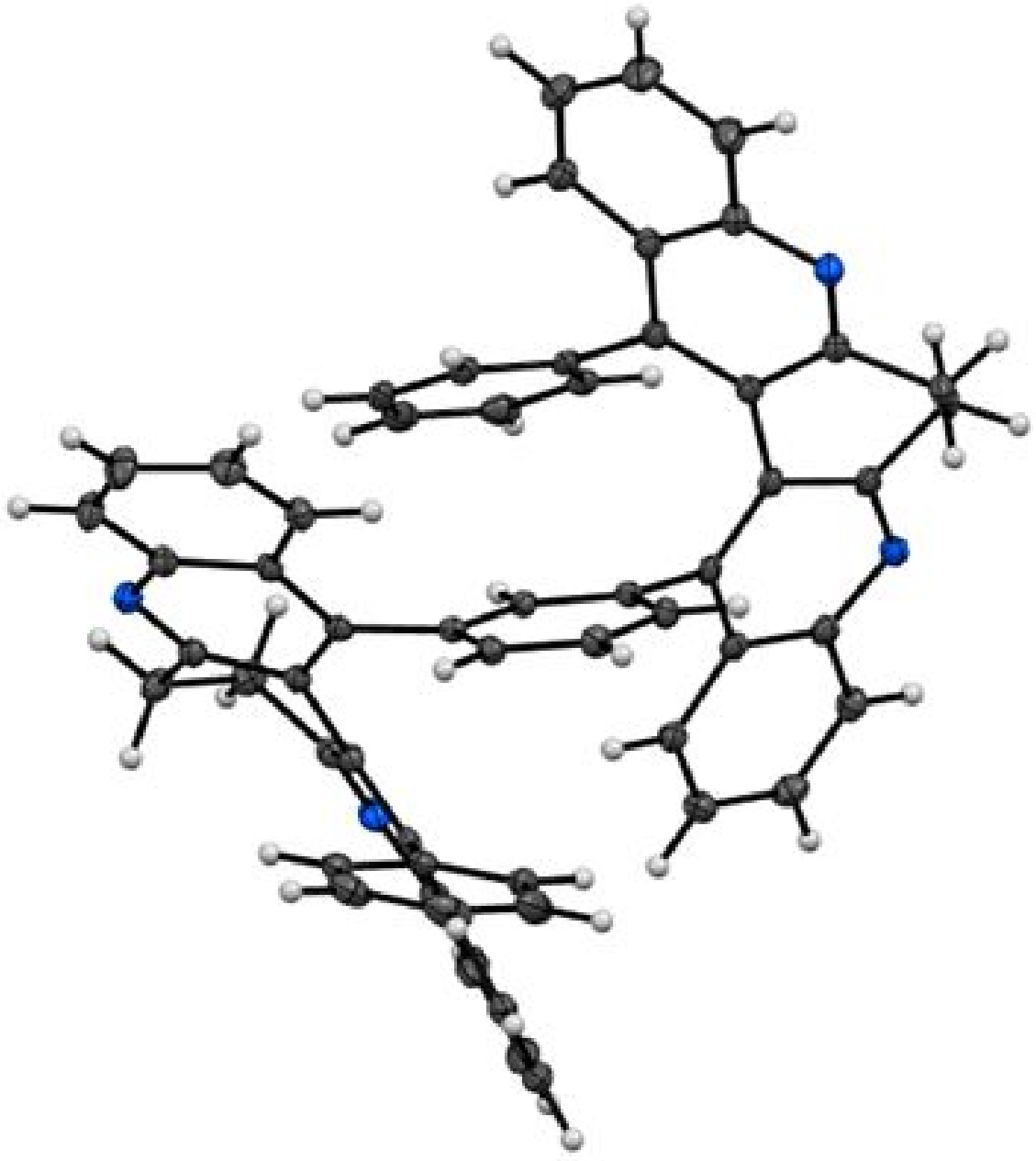

Figure S7: ORTEP graph of compound **13** (thermal ellipsoids at 50% probability)
